# The Peptide Microarray “ChloroPhos1.0” Identifies New Phosphorylation Targets of Plastid Casein Kinase II (pCKII) in *Arabidopsis thaliana*

**DOI:** 10.1371/journal.pone.0108344

**Published:** 2014-10-08

**Authors:** Anna Schönberg, Elena Bergner, Stefan Helm, Birgit Agne, Beatrix Dünschede, Danja Schünemann, Mike Schutkowski, Sacha Baginsky

**Affiliations:** 1 Institute of Biochemistry and Biotechnology, Martin-Luther-University Halle-Wittenberg, Halle (Saale), Germany; 2 Molecular Biology of Plant Organelles, Ruhr-University Bochum, Bochum, Germany; 3 Steinbeis-Forschungszentrum, Peptide Microarrays, Halle (Saale), Germany; Institut de Génétique et Développement de Rennes, France

## Abstract

We report the development of a peptide microarray based on previously determined phosphorylation sites in chloroplast proteins. Altogether, 905 peptides were spotted as 15mers in nine replicates onto glass slides. We used the microarray for *in vitro* phosphorylation experiments and specifically assessed the peptide substrate spectrum of chloroplast casein kinase II (pCKII). To this end, native pCKII from *Arabidopsis thaliana* and *Sinapis alba* chloroplasts was enriched by Heparin-Sepharose chromatography and its activity on the microarray was compared to the activity of a recombinant Arabidopsis pCKII. All three kinase preparations phosphorylated a similar set of peptides that were clearly distinct from those phosphorylated by bovine heart protein kinase A (PKA) in control experiments. The majority of the pCKII phosphorylation targets are involved in plastid gene expression, supporting the earlier denomination of pCKII as plastid transcription kinase (PTK). In addition we identified Alb3 as pCKII substrate that is essential for the integration of light-harvesting complex subunits (LHC) into the thylakoid membrane. Plastid CKII phosphorylation activity was characterized in greater detail *in vitro* with recombinant wildtype Alb3 and phosphorylation site mutants as substrates, establishing S424 as the pCKII phosphorylation site. Our data show that the peptide microarray ChloroPhos1.0 is a suitable tool for the identification of new kinase downstream targets *in vitro* that can be validated subsequently by *in vivo* experiments.

## Introduction

Chloroplasts have to respond quickly to changes in light quality and quantity in order to balance the excitation pressure between the two photosystems. Important aspects of this fast responding system are controlled by posttranslational modifications of resident proteins, e.g. phosphorylation (reviewed in [1]–[3]). Several chloroplast protein kinases are known that catalyze light-triggered acclimation responses, but their complete set of targets and their cross talk in a phosphorylation network are only partially understood. In Arabidopsis, the STN7 and STN8 kinases catalyze the phosphorylation of thylakoid membrane proteins. In addition to its role in controlling state-transitions, STN7 is also involved in the regulation of long-term acclimation processes that entail changes in gene expression of both nuclear and chloroplast encoded genes [4]. Although the STN7-triggered long-term response has been characterized in detail, the exact mechanisms of signal transduction and the mediators involved are still largely unknown. Comparative phosphoproteomics with *stn7* mutants provided no indication for the STN7-dependent phosphorylation of proteins with a function in gene expression [5]. It therefore appears likely that a direct control of plastid gene expression is exerted by other kinases such as the chloroplast sensor kinase CSK or casein kinase II [2].

Plastid casein kinase II (pCKII, formerly also termed CKA4 [6]) activity was originally identified in the 1990's when phosphorylation of an RNA binding protein and the plastid RNA polymerase were reported [7]–[9]. The classification of this phosphorylation activity into the GMGC-group of kinases was based on biochemical properties such as the phosphorylation of serine residues, the ability to utilize GTP as phosphate donor and its specific inhibition by heparin. pCKII activity is associated with the plastid RNA polymerase suggesting a function in the regulation of gene expression by phosphorylation of RNA polymerase subunits and sigma factors [9]–[11]. Indeed, sigma factor phosphorylation influences the efficiency of RNA polymerase promoter binding, supporting a function of pCKII in transcriptional regulation. Henceforth, pCKII was named plastid transcription kinase (PTK). More recently, the gene for pCKII was identified. The gene model AT2g23070 encodes for a CKII alpha subunit that harbors the catalytic activity, possesses a canonical kinase domain and a functional plastid transit peptide [12]. Recent large-scale phosphoproteomics screens identified many proteins that are phosphorylated at characteristic acidic CKII motifs and a statistical evaluation of motif utilization suggested pCKII as the major phosphorylation activity in chloroplasts [13]. However, despite its many putative substrates, pCKII is of very low abundance in photosynthetic chloroplasts, while it accumulates to higher levels in non-photosynthetic organs such as roots [14].

The identification of pCKII substrates is an important first step to understand the function of this kinase. Several distinct methods are available and were already used for the identification of kinase targets. For example, *in vivo* targets for the STN7 and STN8 kinases were identified by quantitative comparative phosphoproteomics analyses between kinase mutant and wildtype [5], [15]. The *in vitro* identification of kinase targets is based on the analysis of phosphorylation activity of a purified kinase or an extract on peptides or proteins. When performed at large-scale, such assays are mostly performed on protein or peptide arrays. Protein arrays are similar to a standard kinase assay in which γ-^32/33^P-ATP is used as a substrate to monitor the phosphate transfer from ATP onto a protein *in vitro* with several protein substrates in parallel [16], [17]. Peptide Arrays are similar to the above assays but instead of proteins, synthetic peptides with a length of around 12–18 amino acids are used as kinase substrates. Such peptides are offered to the kinase either in solution as in the “Kinase Client (KIC)-assay” [18] in which phosphorylated peptides are identified and quantified by mass spectrometry, or immobilized on a glass slide [19], [20]. In both instances, *in vitro* phosphorylation reactions are performed with a purified kinase or a kinase-enriched protein extract.

Here we report the creation of the first chloroplast phosphopeptide microarray ChloroPhos1.0. The peptide microarray was specifically designed for the analysis of chloroplast protein kinases, using plastid phosphoproteomics data for peptide selection and phosphorylation site positioning (http://phosphat.uni-hohenheim.de/) [21]. By selecting peptides that were previously identified in phosphoproteomics experiments, we ascertain that all peptides on the microarrays are true *in vivo* kinase targets. We used the microarray to identify the substrates of pCKII. Our analysis shows that ChloroPhos1.0 is a suitable screening tool to establish currently unknown kinase/substrate relationships in the chloroplast phosphoproteome network. It allows extracting preferred motifs of chloroplast kinases thus facilitating the prediction of additional targets that may have escaped mass spectrometric detection.

## Materials and Methods

### Preparation of the peptide microarray ChloroPhos1.0

We extracted plastid phosphoproteins and phosphopeptides from previously published phosphoproteomics data (Table S1 in File S1) available in PhosphAT 3.0 (http://phosphat.uni-hohenheim.de/) using a chloroplast protein reference table (for details see Results [22]). Peptides were synthesized with a linker at their N-terminus (N-(3-(2-(2-(3- amino-propoxy)-ethoxy)-ethoxy)-propyl)-succinamic acid). Peptide derivatives were synthesized via glycine ester linkage on cellulose membranes in a parallel manner using SPOT synthesis technology [23], [24]. After each coupling, remaining amino functions were acetylated using acetic anhydride in dimethylformamide in the presence of diisopropylethylamine to prevent deletion sequences. Subsequent to removal of the last Fmoc-protecting group with 20% piperidine in dimethylformamide, anthranilic acid was attached to the N-terminus. This coupling reaction transformed desired full-length peptides only, but not acetylated side products resulting from incomplete couplings during peptide synthesis, into 2-aminobenzoyl-derivatives. Following trifluoroacetic acid-mediated side chain deprotection the cellulose bound peptide esters were transferred into 96 well microtiter filtration plates (Millipore, Bedford, Massachusetts, USA) and treated with 200 µL of aqueous triethylamine (2.5% by vol) in order to cleave the peptides from the cellulose. Peptide-containing triethylamine solution was filtered off, and quality controlled by LC-MS, and solvent was removed by evaporation under reduced pressure. Resulting peptide derivatives (50 nmol) were re-dissolved in 25 µL of printing solution (70% DMSO, 25% 0.2 M sodium acetate pH 4.5, 5% glycerol (v/v).) and transferred into 384-well microtiterplates. Peptide derivatives were deposited onto epoxy-functionalized glass slides (PolyAn GmbH, Berlin, 3D-Epoxy, 25×75×1 mm) using the contact printer OmniGrid 300 equipped with 16 SMP2 pins (Telechem/ArrayIt). Our buffer conditions resulted in selective covalent bond formation between epoxy-functions and amino groups of 2-amino-benzoyl-derivatives. Printed peptide microarrays were kept at room temperature for 5 hours. Each microarray with dried deposited spots was analysed using an Axon 4000B microarray scanner. Microarrays which passed this quality control were quenched for 1 hour with sodium citrate buffered 1% BSA solution at 42°C, washed extensively with water followed by ethanol, resulting in ‘purified peptide’ spots, essentially free of deletion sequences (due to acetylation steps during synthesis) and truncated sequences (due to chemoselective immobilization). Printed microarrays were dried using a microarray centrifuge and stored at 4°C.

### Cloning and expression of MBP-tagged pCKII

For bacterial overexpression of *Arabidopsis* cpCK2, pMALc5× (Amp^+^) expression vector was transformed into competent BL 21 DE3 *E.coli* cells. The advantage of this system is the N-terminal MBP (maltose binding protein)-tag on the protein, which enables fast purification with commercially available kits. The cloning of CK2 without transit peptide was done with the following primers NotI_FW, 5′ GTA TAG CGG CCG CGC TTC TCT TTA CCG TCA AC 3′ and BamHI_Rv, 5′ GTA TAG GAT CCT CAC TGG CTG CGC GGC G 3′. At an optical density of 0.5–0.8 at 595 nm, the protein expression was induced by the addition of 1 mM IPTG. The cells were shaken for 3 h at 250 rpm at room temperature and collected. Cell lysis and purification was done according to the NEB pMALc5× purification protocol. After the elution of the purified protein, some contaminating proteins were visible, which we deleted in the second purification step with XK 16/40 Sephacryl S100. A ∼85 kDa band (recombinant pCKII with MBP-tag) was obtained. The fractions were collected and concentrated. Kinase activity was tested with [γ-^33^P] ATP as described below.

### Heparin Sepharose chromatography with *Sinapis alba* and *Arabidopsis thaliana* chloroplast extracts

*Sinapis alba* seedlings were grown on soil under steady light conditions in a controlled environment chamber (24 h light, 150 µE·m^−2^·s^−1^). *Arabidopsis thaliana* Col 0 seedlings were grown on soil under long-day conditions in a controlled environment chamber (16 h light/8 h dark, 150 µE·m−2·s−1). Plants were harvested after 4 days *(S. alba)* or after 17 days *(A. thaliana)*. Chloroplasts were isolated according to established protocols [25], [26]. The isolated chloroplasts were solubilized on ice in a hypotonic buffer (50 mM TRIS-HCl pH 7.6, 4 mM EDTA, 20 mM DTT, 25% glycerol, 0.1% protease inhibitor cocktail (Sigma P9599), 1% Triton ×100). The lysate was adjusted to 200 mM (NH_4_)_2_SO_4_ and applied on a 2-ml Heparin-Sepharose CL-6B column (GE healthcare) equilibrated in buffer E (50 mM TRIS-HCl pH 7.6, 0.1 mM EDTA, 5 mM DTT, 10% glycerol, 0.1% protease inhibitor cocktail (Sigma P9599), 0.1% Triton ×100 and 200 mM (NH_4_)_2_SO_4_). After 2 washing steps with equilibration buffer, the bound proteins were eluted in a single step with 1 M (NH_4_)_2_SO_4_ at a flow rate of 0.4 ml/min. Dialysis was performed overnight at 4°C in buffer E with 20% glycerol and without (NH_4_)_2_SO_4_ [8].

### Kinase activity assays

All fractions were tested for kinase activity as described in [9] with minor modifications. 10 µg proteins were incubated with 5 µg dephosphorylated and heat inactivated stromal proteins as a complex substrate mixture at 30°C for 30 min in kinase activity buffer A (50 mM Tris-HCl pH 7.5, 50 mM NaCl, 10 mM MgCl_2_, 1× PhosStop (Roche) and 0.1% protease inhibitor cocktail (Sigma P9599), 5 µM ATP, 60 nM [γ-^33^P] ATP). In case of recombinant pCKII we used 0.4 µg enzyme for the activity assays. Several controls were performed, in which the indicated ingredient was added or omitted from the reactions. These included omission of Mg^2+^, addition of EDTA, addition of non-radioactive GTP and addition of the CKII inhibitor heparin at a concentration of 15 µg/ml. Following incubation, the reactions were stopped by the addition of SDS sample buffer and the proteins therein were separated by SDS-PAGE [27] and exposed overnight on an autoradiography screen.

### MS analysis of the Heparin Sepharose eluates

Eluted proteins were precipitated in 90% acetone at −80°C and sedimented at 22.000xg for 15 minutes. The protein pellet was dissolved in 25 mM (NH_4_) HCO_3_ and 0.1% RapiGest (Waters) to a final protein concentration of 1.6 µg/µl. In the presence of 10 mM DTT the protein mixture was reduced for 10 min at 60°C and alkylated with 30 mM iodacetamide for 30 min at room temperature in the dark. The tryptic digest was performed overnight at 37°C with a trypsin/protein ratio of 1/100. The digest reaction was acidified by HCl (pH<2) in order to stop tryptic activity and centrifuged for 10 min at 13000 g. The supernatant was analyzed by LC-MS using an ACQUITY UPLC System coupled to a Synapt G2-S mass spectrometer (Waters, Eschborn, Germany).

### Nano-LC separation, HD-MS^E^ data acquisition and protein identification/quantification

LC separation (140 min gradient) and HD-MS^E^ data acquisition was performed using 1 µl of the digested sample on a ACQUITY UPLC System coupled to a Synapt G2-S mass spectrometer (Waters, Eschborn, Germany). MS acquisition was set to 50–2000 Da. Data analysis was carried out by ProteinLynx Global Server (PLGS 3.0.1, Apex3D algorithm v. 2.128.5.0, 64 bit, Waters, Eschborn, Germany) with automated determination of chromatographic peak width as well as MS TOF resolution. Lock mass value for charge state 2 was defined as 785.8426 Da/e and the lock mass window was set to 0.25 Da. Low/high energy threshold was set to 180/15 counts, respectively. Elution start time was 5 min, intensity threshold was set to 750 counts. Databank search query (PLGS workflow) was carried out as follows: Peptide and fragment tolerances was set to automatic, two fragment ion matches per peptide, five fragment ions for protein identification, and two peptides per protein. Maximum protein mass was set to 250 kDa. Primary digest reagent was trypsin with one missed cleavage allowed. According to the digestion protocol fixed (carbamidomethyl on Cys) as well as variable (oxidation on Met) modifications were set. The false discovery rate (FDR) was set to 4% at the protein level. MS^E^ data were searched against the modified *A. thaliana* database (TAIR10, ftp://ftp.arabidopsis.org) containing common contaminants such as keratin and rabbit glycogen phosphorylase B (P00489) was used as internal quantification standard. Quantification was performed based on the intensity of the three most abundant proteotypic peptides. The mass spectrometry proteomics data have been deposited to the ProteomeXchange Consortium (http://proteomecentral.proteomexchange.org) via the PRIDE partner repository [28] with the dataset identifier PXD000981.

### Kinase activity assays on the microarray ChloroPhos1.0

The active kinase preparations were incubated on the microarrays for 2 h at 25°C in kinase activity buffer B (50 mM Tris-HCl pH 7.5, 50 mM NaCl, 10 mM MgCl_2_, 1× PhosStop (Roche) and 0.1% protease inhibitor cocktail (Sigma P9599), 2 µM ATP, 240 nM [γ-^33^P] ATP). These incubations included 25 µg of the recombinant MBP-tagged pCKII, 90 µg of the Heparin Sepharose eluates or 20 units of commercially available bovine heart protein kinase A (PKA, Sigma-Aldrich). The microarrays were washed in TBS pH7.5 to remove remaining substances of the incubation mixture, subsequently by H_3_PO_4_ (pH 2), to get rid of ATP bound to peptides and finally in H_2_O, to neutralize the microarray surface. The dried microarrays were exposed on a Phosphorimager Screen for 1 week and scanned by a BAS-1800 reader (Fujifilm) with 50 µm pixel size. Analysis was performed with the GenePixPro 6.1 software (Molecular devices). The grayscale intensity median of all spot pixels were determined after background subtraction and averaged over all 9 spots/peptide. These signals were classified into 4 categories, which were defined as 3 – strong (>50% over background intensity [BI]); 2 – medium (<50%–>20% BI); 1 – weak (<20%–>10% BI); 0 – none (<10% BI). Due to the possible occurrence of signal artefacts such as dust particles, we included an optical control to exclude false positives.

### Expression of recombinant Alb3 constructs and phosphorylation assays

His-tagged Alb3 wildtype and mutant constructs were expressed and purified as described previously [29]. Site-directed mutagenesis constructs were generated using the QuikChange XL site-directed mutagenesis kit (Agilent Technologies) according to the manufacturer's protocol. Plastid CKII was expressed as His-tagged protein in *E. coli*. The mature sequence of pCKII corresponding to amino acids 56–433 was cloned into the pETDuet1 vector using the BamHI/SalI restriction sites. The plasmid encoding the N-terminally his-tagged pCKII was transformed into *E. coli* Rosetta2 (DE3) (Novagen) and shaken at 37°C until an optical density (600 nm) of 0.6–0.7 was reached. Protein expression was induced by the addition of 1 mM IPTG and continued for 1 h at 30°C. Afterwards the cells were harvested by centrifugation and immediately frozen to −20°C until use. His-pCKII was purified under native conditions using Ni-NTA resin (Qiagen). Protein purification was carried out as the manufacturer suggested using the following buffers: lysis/washing buffer 20 mM Na_2_HPO_4_/NaH_2_PO_4_ pH 8.0, 300 mM NaCl, 5 mM MgCl_2_ and 20 mM imidazole; elution buffer 20 mM Na_2_HPO_4_/NaH_2_PO_4_ pH 8.0, 300 mM NaCl, 5 mM MgCl_2_, 1 mM ATP, 5% (v/v) glycerol and 250 mM imidazole. Alb3 phosphorylation assays for the determination of the phosphorylation site contained 10 µg of His-tagged Alb3 WT or mutant protein and 10 µg of His-pCKII in 20 mM Tris-HCl pH 8.0, 300 mM NaCl, 5 mM MgCl_2_ and 100 µM [γ-^32^P] ATP (1 mM stock solution of non-radioactive ATP spiked with [γ-^32^P]ATP in a final concentration of 0.6 µCi/µl). After incubation for 30 minutes at 30°C the phosphorylation reactions were stopped by the addition of SDS sample buffer and heating. Samples equal to 3 µg of each protein were separated by SDS-PAGE and the dried gels were subjected to autoradiography.

### *In vitro* Phosphorylation kinetics of Alb3 phosphorylation by pCKII

For kinetic studies, we phosphorylated increasing amounts of wildtype Alb3 in two replicates for 2 and 4 minutes at 25°C using 0.3 µM recombinant pCKII. The Alb3 concentration was varied in a range between 50 nM and 5 µM. The reaction was performed in kinase activity buffer (20 mM Tris-HCl pH8; 300 mM NaCl, 10 mM MgCl_2_, 1× PhosStop (Roche) and 0.1% protease inhibitor cocktail (Sigma P9599), 5 µM ATP, 25 nM [γ-^33^P] ATP). All reaction mixtures were separated by SDS-PAGE and analyzed via an autoradiography screen. In parallel a [γ-^33^P] ATP dilution series was spotted onto a dried SDS-gel and exposed on an autoradiography screen in the same way as the phosphorylation reactions. The autoradiograms were analyzed and quantified by the BAS Reader V2.26 and the TINA 2.09 software. The grayscale intensities of the pixels were quantified after background subtraction. Pixel intensities of the ATP-dilution were compared with the Alb3 phosphorylation signal. The resulting PSL values were correlated to the concentration of incorporated [^33^P] thus resulting in quantitative values for phosphate incorporation into Alb3.

## Results

### Design of ChloroPhos1.0: Selection of peptides and positioning of the phosphorylation site

**Figure 1:**
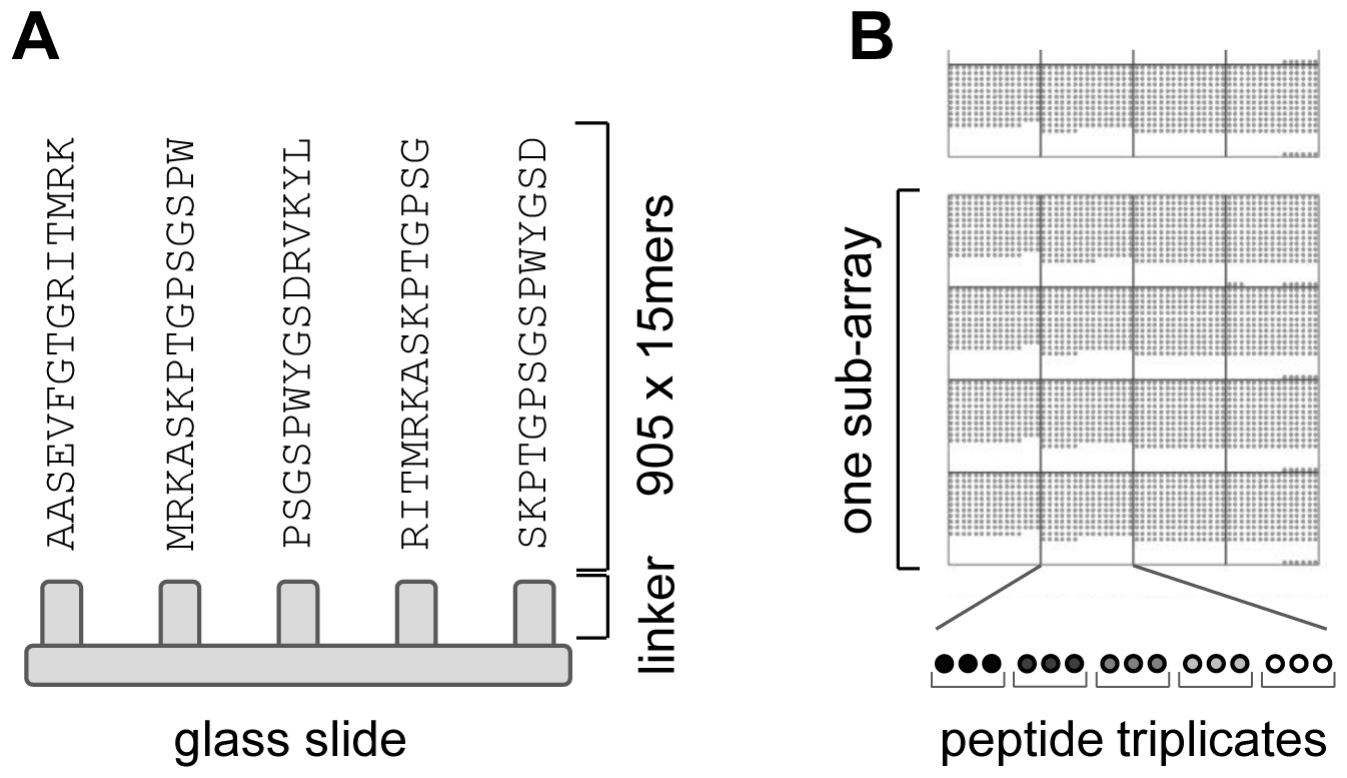
Design of the peptide chip ChloroPhos1.0. (A) Phosphopeptides for chloroplast proteins were extracted from published datasets by searching phosphoproteomics data with a chloroplast proteome reference table. Selected peptides were covalently connected to epoxy- functionalized glass slides via an N-(3-(2-(2-(3- amino-propoxy)-ethoxy)-ethoxy)-propyl)-succinamic acid linker at their N- terminus. The phosphorylation site as determined by mass spectrometry is located at the central amino acid residue in the 15 mer peptide. (B) Organization of peptides on the microarray. The microarray consists of 3 identical subarrays on which 905 chloroplast peptides plus additional control peptides are printed. In each sub-array, every peptide is spotted in triplicates. One peptide spot consists of ∼282 million single peptide molecules, with a spot diameter of 200 µm and an inter-spot separation of 300 µm.

We extracted information on *Arabidopsis thaliana* chloroplast phosphoproteins from different published phosphoproteomics experiments at the status of January 2012, most of them being represented in the PhosPhAT 3.0 database (http://phosphat.uni-hohenheim.de/). Table S1 in File S1 lists all studies that were included for the peptide library generation. The majority of these studies were conducted at the level of the complete cell, only some early MS studies used organelles or isolated proteins for phosphopeptide detection. By means of a chloroplast protein reference table [22] we extracted chloroplast phosphoproteins from these studies. Altogether, we identified 376 phosphoproteins without splice variants. In order to ensure accessibility of phosphorylation sites, we centered the peptides on the phosphorylated amino acid, i.e. we added 7 amino acids upstream and downstream to the phosphorylation site as determined by mass spectrometry. In cases where the phosphorylation site was not unambiguously localized, we allowed all hydroxyl-group carrying amino acids in a phosphopeptide to be present in the center position (Fig. 1). We included two combinatory cases for phosphorylation sites in close proximity in order to decrease the number of different peptides on the microarray. In case of two neighboring phosphorylation sites, only the N-terminal amino acid was centered. In case of two phosphorylation sites separated by one amino acid, the amino acid between the two phosphorylation sites was centered. Phosphorylation sites at the N- or C-terminus of proteins (closer than 8 characters to the terminus) were not centered; instead the suitable 15 mer starting from the N- or C- terminus was built. Redundancies at the peptide level were eliminated. After applying these constraints, our peptide library contained 905 different 15 mers. Some peptide spots as process- or incubation- controls were added to better assess kinase activity on the microarray. Table S2 in File S1 lists all peptides spotted on the microarray. All peptides were synthesized with a linker at their N-terminus (N-(3-(2-(2-(3- amino-propoxy)-ethoxy)-ethoxy)-propyl)-succinamic acid).

### Phosphorylation activity of recombinant pCKII on the peptide microarray ChloroPhos1.0

Although casein kinase II activity is the major kinase activity in chloroplast extracts, we were unable to detect phosphorylation activity on the microarray with chloroplast protein preparations. In these experiments, we assayed up to 6 mg chloroplast protein, either from solubilized chloroplasts, where Triton X-100, or ß- DDM were utilized as nonionic detergents, or from detergent-free stroma extracts (data not shown). This is most likely due to low kinase concentrations and difficulties with the phosphorylation of peptide substrates because of competition from suitable protein substrates in close proximity to the active kinase. We therefore decided to enrich native chloroplast CKII by Heparin-Sepharose chromatography [9] and expressed recombinant *Arabidopsis thaliana* pCKII in *E. coli* as a control. Phosphorylation activity of these two kinase preparations on the microarray was determined to identify pCKII phosphorylation targets. MBP-tagged pCKII was overexpressed in *E. coli* and purified in two steps by utilizing the maltose-binding protein tag on an Amylose column in the first step and by size-exclusion chromatography on Sephacryl S100 in the second step (Fig. S1 in File S1). Activity tests using 2 µg casein as substrate show kinase activity of the recombinant protein and experiments with GTP as a phosphate donor and heparin as inhibitor reveal the specific CKII characteristics of this phosphorylation activity (Fig. S1 in File S1).

**Figure 2:**
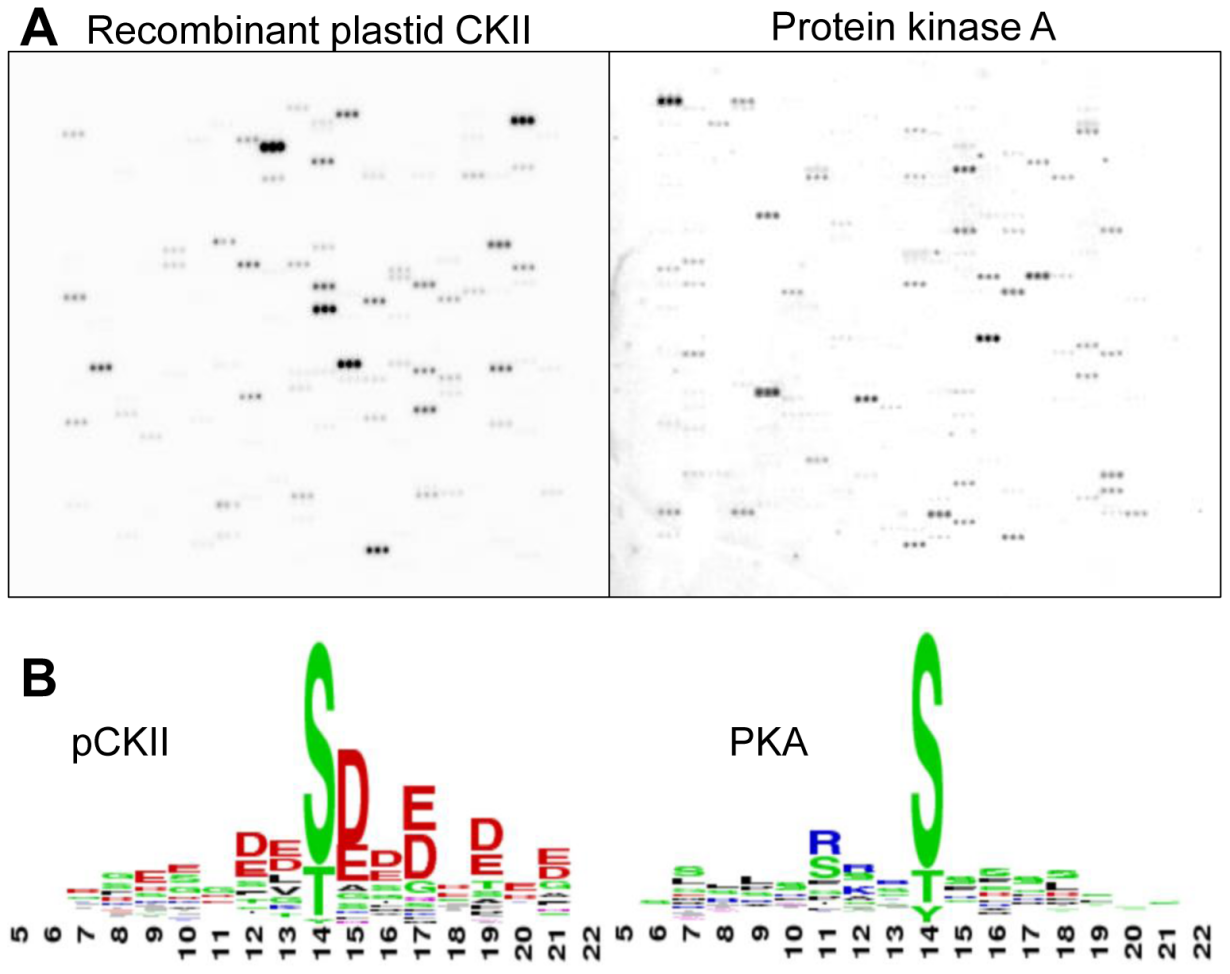
Microarray assay with recombinant pCKII and protein kinase A (PKA). (A) Shown is the autoradiography result obtained with one sub-array. (B) Phosphorylation motif used by the different kinases. Depicted are the weblogos (http://weblogo.berkeley.edu/) of signal peptides with centered phosphorylation sites in 3 bits latitude.

We performed a first microarray experiment and compared pCKII activity with that of bovine heart protein kinase A (PKA) to assess phosphorylation activity and specificity (Fig. 2 A). PKA phosphorylated 43 peptides and pCKII phosphorylated 33 peptides (Table S3 in File S1). There was no overlap in peptide phosphorylation between these two activities suggesting high specificity of these two kinases with peptides on the microarray. We extracted the phosphorylation motifs from the set of phosphorylated peptides by WebLogo (http://weblogo.berkeley.edu/) and found that PKA prefers a motif with basic residues at positions −3 to −1 relative to the phosphorylation site, while pCKII prefers motifs with acidic residues at positions +1 to +3 relative to the phosphorylation site (Fig. 2 B). These motifs are in good agreement with alignments for these two kinases obtained from a collection of their phosphorylation targets in other systems [20], [30]. In conclusion, recombinant pCKII recognizes its bona fide substrates on the microarray and does so with a clear substrate preference that differs from that of PKA (Table S3 in File S1).

### Preparation of native pCKII from *Arabidopsis thaliana* and *Sinapis alba*

**Figure 3:**
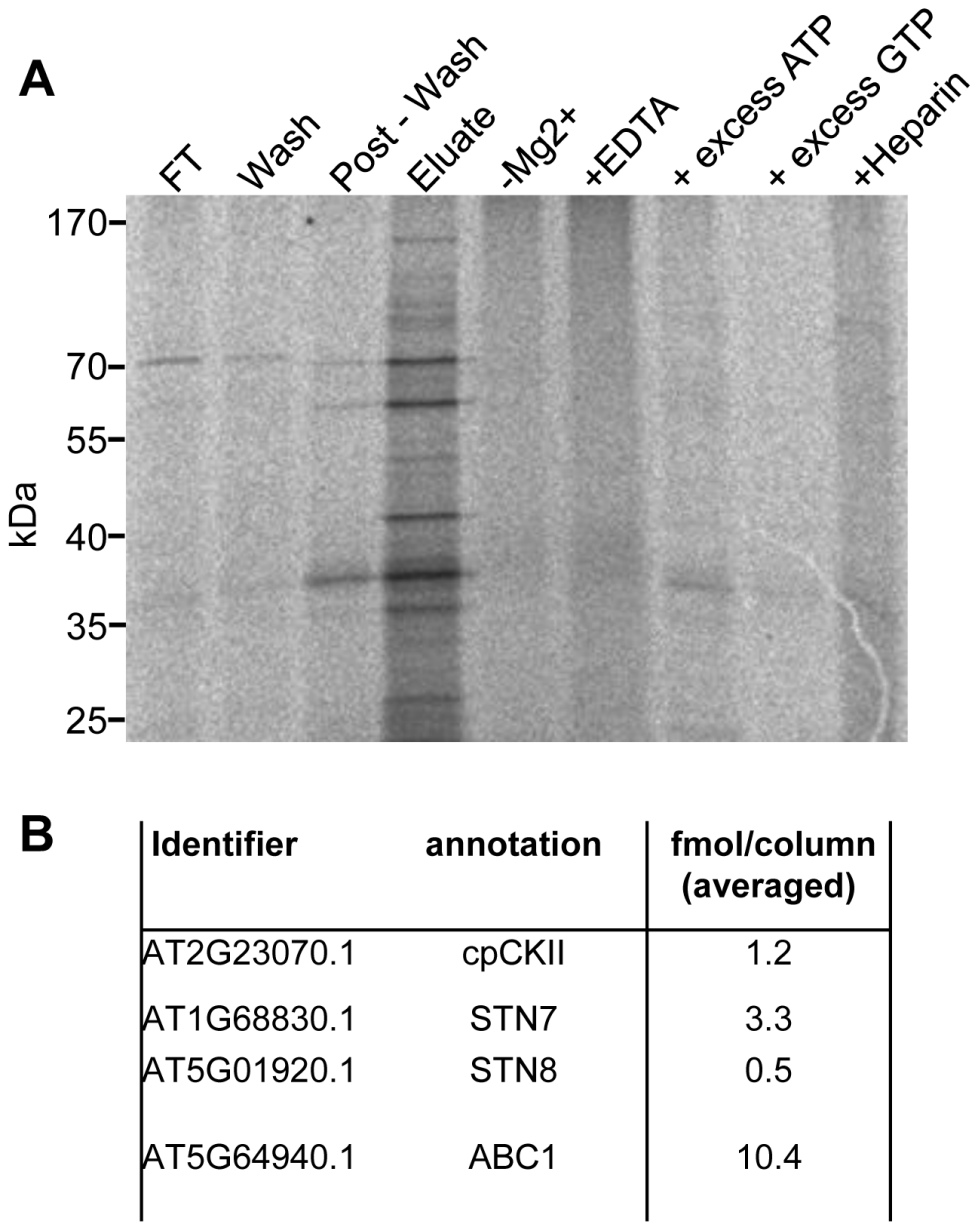
*In vitro* phosphorylation activity of native pCKII preparations. (**A**) Autoradiography result of an activity assay with native pCKII isolated from Arabidopsis chloroplasts by Heparin-Sepharose chromatography with dephosphorylated and heat inactivated stroma proteins as substrates. Lanes 1–4 show the different chromatography fractions: flow through (FT), wash, post-wash and eluate. Lanes 5–9 present controls with the kinase active eluate fraction. Control reactions contained, no MgCl_2_, 1 mM GTP, 15 µg/ml heparin, 1 mM ATP and 5 mM EDTA where indicated. (**B**) Concentration of kinases in the active Heparin-Sepharose fractions determined by triplicate HD-MSE detection (see Materials and Methods and Table S4 in File S1 for further details).

We next analyzed whether native pCKII isolated from chloroplast extracts phosphorylates the same set of peptides as the recombinant enzyme. To this end, we isolated pCKII from *Sinapis alba* and *Arabidopsis thaliana* chloroplasts by Heparin-Sepharose chromatography. *Sinapis alba* was introduced as a preparation control because the pCKII (PTK) purification protocol was originally established for this plant [9]. The isolation of pCKII from Arabidopsis used the same method as described for *Sinapis alba* with minor modifications. The enrichment of pCKII in the eluates was analyzed *in vitro* using dephosphorylated and heat inactivated stroma extracts as substrate mixture. The specificity of the kinase preparation was assessed in control experiments using heparin as inhibitor and an excess of non-radioactive GTP as phosphate donor. These data indicate that pCKII was successfully enriched in the eluate fractions (Fig. 3A). To assess the efficiency of pCKII enrichment we identified and quantified proteins in the eluate fraction by mass spectrometry as previously described [31]. Altogether, 518 proteins were identified. Chloroplast ribosomal proteins and CSP41 are the most abundant proteins in these fractions followed by components of the transcription machinery (Table S4 in File S1). Despite successful enrichment, the pCKII amount is relatively low with 1.2 fmol measured in 1.5 µg chloroplast protein on column. In 1.5 µg chloroplast eluate protein, we also identified the thylakoid kinases STN7 (3.3 fmol on column) and STN8 (0.5 fmol on column) and the plastoglobuli associated kinase ABC1K8 (10.4 fmol on column) (Fig. 3 B).

### Phosphorylation activity of different pCKII preparation on the peptide microarray ChloroPhos1.0

**Figure 4:**
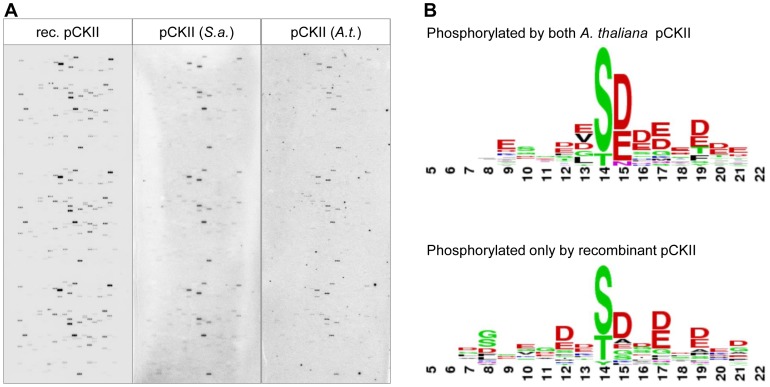
Peptide Chip analysis of preferred pCKII phosphorylation motifs. (**A**) Peptide Chip results obtained with the different pCKII preparations (**B**) Phosphorylation site alignment of the peptides used by all pCKII preparations as substrate (upper panel) and peptides phosphorylated exclusively by recombinant pCKII (lower panel). Depicted are the weblogos (http://weblogo.berkeley.edu/) of peptides giving a signal above background (see Materials and Methods for details) with centered phosphorylation sites in 3 bits latitude.

We tested the phosphorylation activity of the three different pCKII preparations on the peptide microarray and found a robust set of peptides that are phosphorylated by all kinase preparations (Fig. 4 A, Table S5 in File S1 and Table 1). Most importantly, the native pCKII preparation phosphorylated the same peptides as the recombinant pCKII. The common substrates of the recombinant pCKII and the native pCKII preparations are e.g. peptides of translation elongation factor EF1B, Alb3, Toc159, Tac10 and RH3. Unique targets of recombinant pCKII comprise furthermore unknown proteins, RNP31, several metabolic enzymes and Mg^2+^-chelatase (Table 1). Several peptides are exclusively phosphorylated by the recombinant pCKII, which is most likely a consequence of its higher specific kinase activity. In this case we expect that the peptides phosphorylated by all three preparations are better pCKII substrates or contain more pCKII phosphorylation sites, because they apparently require less pCKII activity to produce a detectable phosphorylation signal. The peptides phosphorylated by all preparations do not contain more S, T or Y compared to those phosphorylated exclusively by the recombinant enzyme but they are more acidic and comply better with the canonical CKII consensus motif with acidic amino acids in position +1 to +3 relative to the phosphorylation site (Fig. 4 B). The peptides phosphorylated only by the recombinant enzyme are therefore most likely less favorable CKII substrates.

**Table 1:** Phosphorylation targets of pCKII on the peptide microarray ChloroPhos1.0.

			Phosphorylation category		
Identifier	Annotation	Peptide	native enzyme (HS-A.t.)	recombinant pCKII	PKA
AT1G58200	MSCS-like 3	KAEKDEV**S**DDEA**T**IE	2	1	0
AT1G64510	EF1B	FFEGGFG**S**DDDP**TS**P	2	3	0
AT2G28800	Alb3	Q**S**E**S**EEG**S**DDEEEEA	2	2	0
		VEE**S**Q**S**E**S**EEG**S**DDE	2	2	0
AT2G35490	Plastid-lipid associated protein	**T**DEWGEK**S**GPELEES	0	1	0
AT2G36390	starch branching enzyme 2.1	GFNRLDD**S**AEFF**TS**D	0	1	0
AT2G39730	rubisco activase	RWRGLA**Y**D**TS**DDQQD	1	1	0
		RGLA**Y**D**TS**DDQQDI**T**	0	1	0
AT3G09050	unknown protein	GDG**TS**D**S**D**S**DPDPPK	0	2	0
		L**S**K**S**GDG**TS**D**S**D**S**DP	0	2	0
AT3G48500	Tac10	K**Y**EGKKL**S**EL**S**DDED	3	2	0
		GKKL**S**EL**S**DDEDFDE	2	2	0
AT3G52230	unknown protein;	KDDDEDDQ**SS**DGHED	0	1	0
AT4G02510	Toc159	G**S**E**S**EEE**T**EEMIFG**S**	2	3	0
		V**T**RVDG**S**E**S**EEE**T**EE	1	1	0
		IDGQIV**T**D**S**DEDVD**T**	1	1	0
AT4G04020	fibrillin	PDFKIRA**T**DIDDEWG	0	1	0
AT4G12610	transcription activators	DEEEGNV**S**DRGDEDE	1	1	0
AT4G24770	31RNP	DA**S**EGDV**S**EGDE**S**EG	1	2	0
		DV**S**EGDE**S**EGDV**S**EG	0	1	0
AT5G08540	unknown protein	N**SS**VEEE**T**EEEVEED	0	1	0
AT5G13630	magnesium-chelatase (chlH)	G**S**DKGIL**S**DVELLKE	0	1	0
AT5G14740	carbonic anhydrase 2	VLAE**S**E**SS**AFEDQCG	0	1	0
AT5G19390	Rho GTPase	KGFVADD**S**DIE**S**PRD	0	1	0
		DNEVEPV**T**DDDNDRA	0	1	0
AT5G26742	DEAD box RNA helicase (RH3)	AFK**S**LGL**S**DHDE**Y**DL	1	3	0
		L**S**EEAFK**S**LGL**S**DHD	0	2	0
		GL**S**DHDE**Y**DLDGDNN	0	2	0
AT5G38410, AT5G38420, AT5G38430	RubisCO SSU	L**SY**LPDL**S**DVELAKE	1	2	0
AT5G49910	cpHsc70-2	DNGGDVIDADF**T**D**S**N	1	1	0
AT5G53170	FTSH protease 11	FVGGEE**T**K**S**GGEEAE	0	1	0
AT5G61210	SNAP33	PFD**S**DDE**S**DNKH**T**LN	2	3	0
		**S**KPNPFD**S**DDE**S**DNK	2	2	0

The phosphorylation category was determined by calculating the grayscale intensity median of all spot pixels after background subtraction. These intensity values were averaged over all 9 peptide spots and classified into 4 categories. The categorries are defined as 3 - strong(>50% segregation of background intensity [BI]); 2- medium(>20%–<50% BI); 1 - weak(>10%–<20% BI); 0 – none(<10% BI).

In only two instances we observe a stronger peptide phosphorylation signal with the native Arabidopsis pCKII compared to the recombinant protein, i.e. with peptide KAEKDEVSDDEATIE of MSCS-LIKE 3 (AT1G58200) and peptide KYEGKKLSELSDDED of TAC10 (AT3G48500) (Fig. 4 A and Table 1). Both peptides fulfil the requirements for acidic amino acids in a CKII phosphorylation motif and it is therefore not clear why the highly active recombinant pCKII is less active on them compared to the native enzyme. At this stage we cannot rule out the possibility that one of the kinases in the Heparin-Sepharose eluates are involved in the phosphorylation of the second serine or threonine in each peptide (see peptides above). This could make the peptide more acidic and thus elevate the phosphorylation activity by native pCKII. Such a “priming” phosphorylation would be missing in the recombinant enzyme preparation. It is also possible that pCKII interacts with proteins in the chloroplast extract and that this interaction alters its substrate preference for some of the substrates. Although at present speculative, both explanations make an excellent starting point for further investigations.

### *In vitro* phosphorylation of recombinant Alb3 validates peptide phosphorylation data

**Figure 5:**
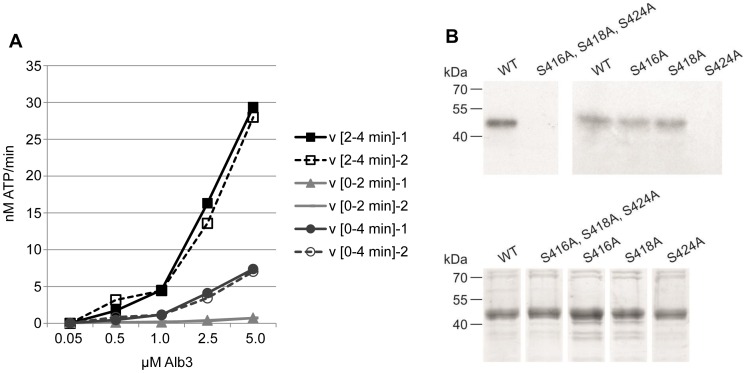
Phosphorylation of Alb3 by pCKII. (**A**) Alb3 phosphorylation kinetic with recombinant pCKII. Depicted is the mean of the two replica and their maximum deviation. Incorporation of ATP was detemrined by cross-relating the pixel intensity of phosphorylated Alb3 with an [^33^P] ATP dilution series. (**B**) *In vitro* phosphorylation of wildtype Alb3 and the phosphorylation site mutants S416A, S418A, and S424A. For the phosphorylation assays we used the single mutants and the triple mutant as indicated. The upper panel shows the autoradiograph of the phosphorylation results, the lower panel the Coomassie stain of the individual protein preparations.

The set of pCKII targets comprises proteins of the gene expression apparatus and proteins involved in chloroplast metabolism, suggesting that pCKII may catalyze crosstalk between different chloroplast functions. We selected Alb3 for further characterization because its phosphorylation by pCKII suggests a so far unknown type of crosstalk between the regulation of gene expression and thylakoid membrane assembly *in vivo* [32]. We expressed Alb3 in *E. coli* with its wildtype sequence and as the phosphorylation site mutants S416A, S418A and S424A (Fig S2 in File S1), and used affinity purified and desalted Alb3 protein for *in vitro* phosphorylation assays. Using recombinant MBP-tagged pCKII, we phosphorylated increasing concentrations of wildtype Alb3 for 2 and 4 minutes in two replicates each (Fig. 5 A). Under these conditions, the maximum velocity of the reaction (Vmax) was not reached at substrate concentrations up to 5 µM. Plastid CKII phosphorylates Alb3 efficiently as illustrated by the high velocity of phosphate incorporation into recombinant wildtype Alb3 at incubation times between 2 and 4 minutes (Fig. 5 A). The phosphorylation of Alb3 by the recombinant pCKII shows the specific properties of CKII phosphorylation activity because it can be competed with unlabeled GTP and is inhibited by low concentrations of heparin (Figure S3 in File S1).

To determine the exact site of pCKII phosphorylation we exchanged the serines S416, S418 and S424 in the Alb3 wildtype sequence with alanine (Fig. S2 in File S1). The S414A and S418A mutant proteins are phosphorylated by recombinant His-tagged pCKII up to wildtype levels, suggesting that these sites are not targeted by pCKII (Fig. 5 B). In contrast, phosphorylation assays with the S424A mutant resulted in a complete loss (His-tagged pCKII) or a greatly diminished phosphorylation signal (MBP-tagged pCKII) (Fig. 5 B and Figure S3 in File S1) depending on the specific activity of the pCKII preparation. This establishes S424 as the major pCKII phosphorylation site in Alb3, which is in line with the phosphopeptide mapping data for Alb3 reported by Reiland and colleagues [13]. The residual phosphorylation observed with the MBP-tagged pCKII preparation is most likely due to phosphorylation of S420, which is placed in a putative CKII phosphorylation motif with acidic amino acids at positions −1 and +1 to +2 relative to the serine residue. The phosphorylation site S424 shows a clear preference of pCKII for the motif E-X-S-D-D-E (Fig. S2 in File S1) for Alb3 phosphorylation. Such a motif in the C-terminal, stroma exposed region of Alb3 is present in *Arabidopsis thaliana*, *Zea mays*, *Hordeum vulgare* and one *Oryza sativa* homologue, but is missing from *Pisum sativum* and *Solanum tuberosum* and from the two *Chlamydomonas reinhardtii* homologues [33] (Fig. S4 in File S1).

## Discussion

### Using peptide microarrays as screening tools for kinase targets

We show here that chloroplast phosphoproteomics data are a useful resource for the design of an organelle-specific peptide microarray. The phosphoproteomics results were collected from published large-scale experiments with different Arabidopsis samples, most of them deposited in the PhosPhAT database (http://phosphat.uni-hohenheim.de/). By exclusively using peptides that were found phosphorylated *in vivo*, we increase the specificity of a positive *in vitro* phosphorylation result on the microarray with a chloroplast protein kinase. By combining prior knowledge on the few established chloroplast kinases with knowledge about their *in vivo* targets, we introduce a meaningful constraint in the design of a large-scale experiment. Such constraints reduce false assignments of kinase/substrate relationships, as previously shown for kinase motif prediction [34]. We are thus confident that the peptides phosphorylated by pCKII in the experiments reported here are true *in vivo* substrates of this kinase, provided that the constraints are fulfilled (this is not the case for the phosphorylation of peptides by PKA, because the kinase does not co-localize with the substrates *in vivo*). The specificity of this assignment is supported by the fact that we do not find any overlap in the set of phosphorylated peptides between pCKII and PKA (Fig. 2).

However, there are a few issues that need attention in the interpretation of the *in vitro* results and their transfer to the *in vivo* situation. The specificity and efficiency of a kinase reaction with its substrate is determined at different levels *in vivo*, some of which cannot be assessed with a peptide microarray [35]. These levels of regulation entail interaction of the kinase with its substrate via docking sites or scaffold proteins. Docking sites are often spatially separated from the kinase active site and the actual phosphorylation sites and they increase the local concentration of substrate in the vicinity of the protein kinase. Thus, docking sites may be required to facilitate the phosphorylation of a substrate *in vivo*. Since they are not represented in the 15mer peptide on the microarray, we expect phosphorylation to occur at lower rate. In fact, the lack of peptide phosphorylations with crude extracts and the necessity to enrich kinases for their use on the microarray support this view. The same holds true for scaffold proteins that are required to establish a productive kinase/substrate interaction. In both cases, “false negative” results are the consequence. With the peptide microarray we furthermore neglect the concentration ratio between kinase and its substrate that exists *in vivo*. This may result in “false positive” assignments because protein concentrations are a potential regulator of kinase activity *in vivo*, that may operate for example by substrate competition [35].

The primary determinant of kinase substrate specificity is the catalytic cleft, e.g. its depth, hydrophobicity and charge distribution. Based on the depth of the catalytic cleft, kinases are roughly grouped into two different families, serine/threonine kinases on the one hand and tyrosine kinases on the other hand. Although the catalytic domains are similar, tyrosine kinases have a deeper catalytic cleft that allows a tyrosine residue to span the distance between the substrate peptide backbone and the y-phosphate of the phosphate donor [36], [37]. Apart from the phosphorylated residue, the amino acids that are directly neighboring the phosphorylation site are the most important specificity determinants for the recognition of a substrate by its kinase [35]. It was shown, that the active site of a kinase interacts roughly with four amino acids up- and downstream of the phosphorylation site. In the case of CKII, basic residues in subdomains VIII, I, II and III are predicted to make contact with the acidic amino acids of the substrate, such determining the high specificity of CKII for acidic residues proximal to the phosphorylation site [38] (subdomain numbering system according to [39]. This substrate specificity is reflected in the consensus motif of CKII phosphorylation in general, and it was correctly reproduced on the peptide microarray data reported here (Figs. 2 B and 4 B).

### The set of chloroplast pCKII targets

The set of phosphorylated peptides on the microarray is relatively small, i.e. much smaller than the set of predicted CKII targets based on phosphorylation motif recognition [13]. For reasons detailed above we conclude that we have assembled a highly stringent dataset with a high number of false negative observations, i.e. peptides that were not phosphorylated *in vitro* although they are targets for pCKII *in vivo*. Exceptions are the phosphorylations of Toc159 and SNAP33, both of which are strongly phosphorylated by all pCKII preparations on the microarray. These proteins are normally not in contact with pCKII because the acidic A-domain of Toc159 is reaching into the cytosol and SNAP33 (AT5G61210) is an endosomal contaminant in the chloroplast reference proteome. Nonetheless, these proteins are excellent targets for CKII and it is very likely that they are phosphorylated by one of the other CKII alpha subunits in the nucleo-cytoplasmic compartment [12]. The set of pCKII targets comprises several proteins involved in plastid gene expression. TAC10 is an essential component of the plastid RNA polymerase complex containing an S1 RNA-binding domain. Its exact function is currently unknown [40]. The phosphorylation site is located C-terminal to the S1 domain at Ser431 and/or Ser434. The function of phosphorylation is not known, but early analyses revealed that phosphorylation of the plastid RNA polymerase complex by pCKII results in a decrease of *in vitro* transcription activity [41], [42]. The hypothesis that pCKII phosphorylation of the RNA polymerase has an inhibitory effect aligns with the higher concentration of pCKII in non-photosynthetic organs [14]. Here, phosphorylation of the RNA polymerase could prevent the transcription of genes for photosynthetic proteins, that are not needed e.g. in roots.

A function of pCKII in the regulation of posttranscriptional processes of plastid gene expression is supported by the phosphorylation of the DEAD box RNA helicase RH3. RH3 has a predicted transit peptide length of 60 amino acids and the phosphorylation site is located at Ser80. Thus, this phosphorylation site is N-terminal to the helicase domain and close to the N-terminus of the mature protein. Because of this location it seems unlikely that phosphorylation alters the catalytic properties of RH3. It is more likely that phosphorylation alters its interactions with other proteins in RNA degrading complexes. The RNA-binding protein RNP31 is already a known pCKII target. This protein belongs to a family of RNA-binding proteins that have an acidic domain and two tandem RNA recognition motifs (RRM). They were among the first identified pCKII targets in chloroplasts [7]. Phosphorylation by pCKII occurs in the acidic domain at Ser128 and is known to alter the RNA binding properties by this RNP family [43].

In addition to targets in the gene expression system, pCKII appears to be involved in the regulation of the plants central energy metabolism. This entails the carbohydrate metabolism via phosphorylation of the starch branching enzyme (SBE2.1), the small subunit of RubisCO, and RubisCO activase, and photosynthesis via phosphorylation of Alb3. A regulatory role of CKII in carbohydrate metabolism was reported earlier for mammalian systems. Here, CKII phosphorylates glycogen synthase, phosphoglucose isomerase and glycerol-3-phosphate acyltransferase (GAT) [44]. A new and previously unreported function of pCKII in the regulation of photosynthesis may be mediated by phosphorylation of Alb3. Alb3 is essential for the integration of light-harvesting complex proteins into the thylakoid membrane and lack of Alb3 results in an albino phenotype [45]. This protein is well conserved in land plants and algae and structurally related to bacterial YidC proteins [31]. Using recombinant Alb3, we could establish Ser424 as the major site for phosphorylation (Fig. 5). This phosphorylation site is not strictly conserved (Fig. S4 in File S1) suggesting specialization in the regulation of LHC complex insertion into thylakoid membranes.

## Conclusions

We show here that the peptide microarray ChloroPhos1.0 is a suitable screening tool to identify novel kinase substrates and to characterize the preferred phosphorylation motif of currently uncharacterized plastid kinases. By using *in vivo* phosphorylation sites of proteins that co-localize with chloroplast kinases, we aim for stringent substrate recognition and a low false positive rate in the assignment of *in vivo* kinase substrates. This requires that the constraints are correct, i.e. that the substrates truly co-localize with the kinase, which is not the case for PKA. At the same time, we expect a relatively high false negative rate for structural reasons such as the lack of interaction domains and docking sites that facilitate substrate recognition by its cognate kinase *in vivo*. The fact that we were unable to measure phosphorylation activity with complex extracts supports this hypothesis. Because of its design, the microarray is restricted to assay targets for Arabidopsis chloroplast protein kinases and may be extended to the analysis of closely related species such as mustard. We will constantly update the microarray and the number of peptides will increase as more information about chloroplast protein phosphorylation becomes available.

## Supporting Information

File S1 **File S1 includes the following: Figure S1.** Purification and activity assay of recombinant pCKII from E coli. **Figure S2.** Potential phosphorylation sites in the C-terminal region of Alb3. **Figure S3.** *In vitro* phosphorylation of Alb3. **Figure S4.** Excerpt of a multiple sequence alignment of Alb3 homologs in different plant species. **Table S1.** MS-based phosphoproteomics studies used for the peptide library. **Table S2.** List of peptides included on the microarray ChloroPhos1.0. **Table S3.** List of peptides phosphorylated by recombinant pCKII and protein kinase A. **Table S4.** Proteins identified and quantified from the Heparin-Sepharose eluate fraction with kinase activity. **Table S5.** List of peptides phosphorylated by recombinant pCKII and the isolated native plastid casein kinases from *Arabidopsis thaliana* (A.t.) and *Sinapis alba* (S.a.).
(PDF)
