# Supplementary material for: The Peptide Microarray “ChloroPhos1.0” Identifies New Phosphorylation Targets of Plastid Casein Kinase II (pCKII) in Arabidopsis thaliana
Source: PLoS One. 2014 Oct 8;9(10):e108344. doi: 10.1371/journal.pone.0108344 (PMC4189921; doi:10.1371/journal.pone.0108344)
Supplement: File S1 — includes the following: Figure S1. Purification and activity assay of recombinant pCKII from E coli. Figure S2. Potential phosphorylation sites in the C-terminal region of Alb3. Figure S3. In vitro phosphorylation of Alb3. Figure S4. Excerpt of a multiple sequence alignment of Alb3 homologs in different plant species. Table S1. MS-based phosphoproteomics studies used for the peptide library. Table S2. List of peptides included on the microarray ChloroPhos1.0. Table S3. List of peptides phosphorylated by recombinant pCKII and protein kinase A. Table S4. Proteins identified and quantified from the Heparin-Sepharose eluate fraction with kinase activity. Table S5. List of peptides phosphorylated by recombinant pCKII and the isolated native plastid casein kinases from Arabidopsis thaliana (A.t.) and Sinapis alba (S.a.). (PDF) [file pone.0108344.s001.pdf]

Fig. S1

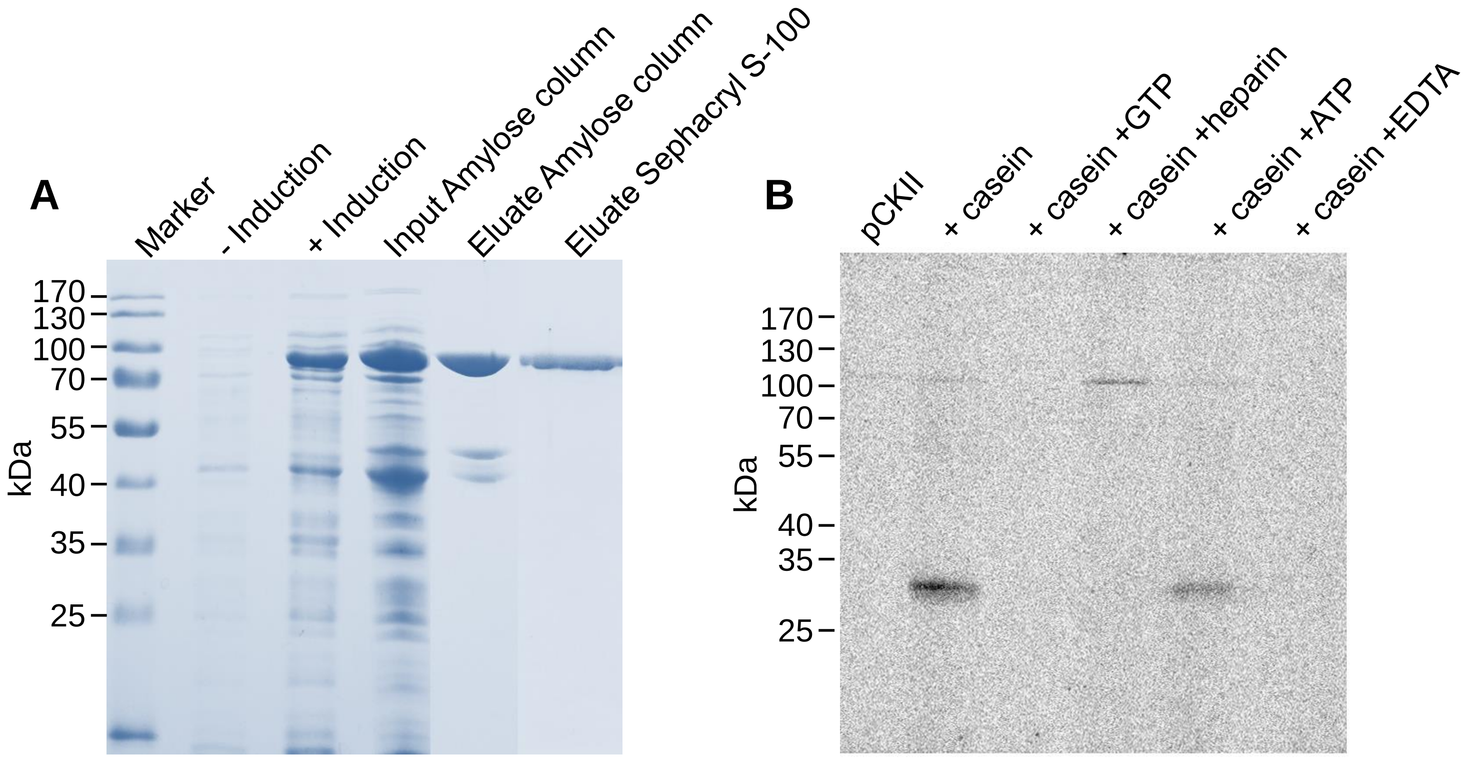

Fig. S1: Purification and activity assay of recombinant pCKII from E coli. (A) Purification of pCKII from E. coli. Shown is the colloidal Coomassie stain of the indicated fractions. (B) Result of the activity test in the presence of 2 µg casein as substrate. Controls included 1 mM GTP, 15µg/ml heparin, 1 mM ATP and 10 mM EDTA.

Fig. S2

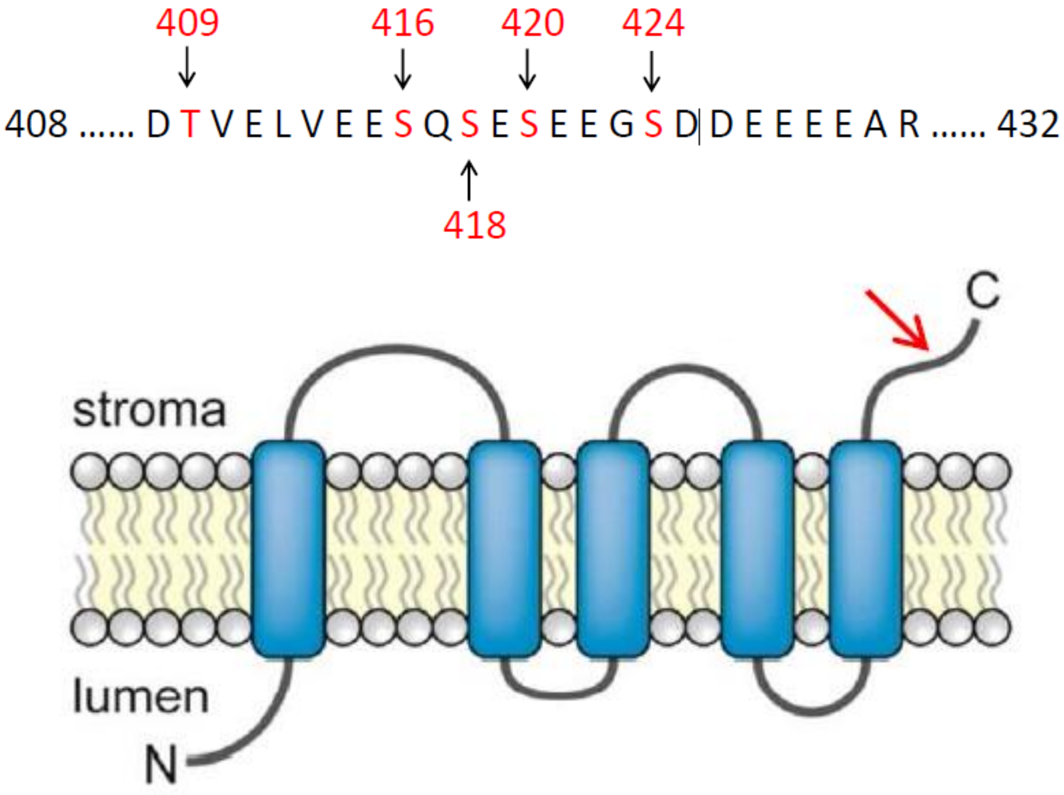

Fig. S2: Potential phosphorylation sites in the C-terminal region of Alb3. The indicated serines were exchanged with alanine in order to determine the exact site of phosphorylation. The topology of Alb3 is shown in the figure below.

Fig. S3

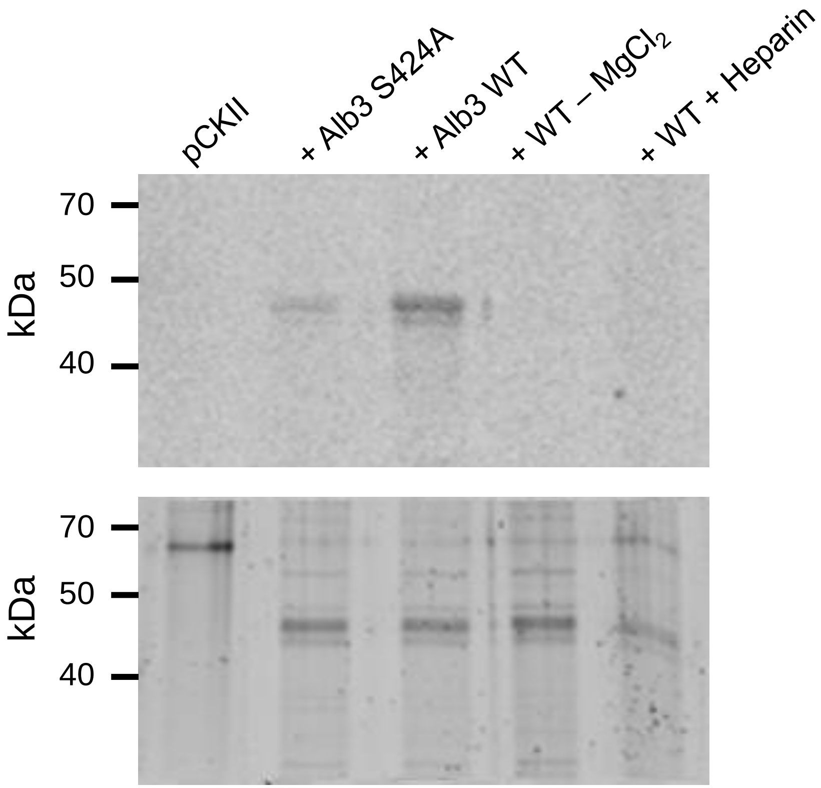

Fig. S3: The upper panel shows the autoradiograph of the Alb3 phosphorylation by MBP-tagged pCKII. The Alb3 S424A mutant was phosphorylated side-by-side with the wildtype Alb3. The panel below shows the colloidal Coomassie stain of the radioactive gel as a loading control. Controls were performed that omitted MgCl2 or contained 15µg/ml heparin as indicated. These controls were performed with wildtype Alb3.

Fig. S4

```
Arabidopsis         -----DDPAQKNTLLVFKFLPLMIGYFALSVPSGLSIYWLTNNVLSTAQQVYLRKLGGAK     359
Solanum_1           -----DDPSQKNTLLVFKFLPLMIGYFSLSVPSGLTIYWFTNNVLTTAQQVWLRKLGGAK     342
Solanum_2           GMLLSDDPNVKNSQAITKFLPLMLGYFSLSVPSGLSLYWLTNNILSTGQQVWLQKLGGAK     383
Pisum               -----NDPNQKNTLLIFKFLPLMIGYFSLSVPSGLTIYWFTNNVLSTAQQVWLRKLGGAK     337
Hordeum             -----TDPSQKNTQLILKFLPFMIGYFSLSVPSGLSIYWFTNNVLSTAQQIWLRKLGGAK     346
Zea                 -----DDPSQKNTMLILKFLPFMIGWFSLSVPSGLSIYWFTNNVLSTAQQVWLRKMGGAK     353
Oryza_1             -----NDPSQQGAQAVVKFLPLLIGYFALSVPSGLSLYWLTNNILSTAQQVWLQKLGGAK     346
Oryza_2             -----DDPSQKNTLLVLKFLPFMIGWFSLSVPSGLSIYWFTNNILSTAQQVWLRKLGGAK     356
Chlamydomonas_1     -----KDENANTQRALLVFLPLMVGWFSLNVPAGLSLYYLANTVLSSAIQIYLKKLGGAN     363
Chlamydomonas_2     -----QDPSQQQAQAILKFLPLMIGWFSLNVPSGLTLYWFVNNLLSTGQQLYLKATVKVN     315
                          *   :    :  ***:::*:*:*.**:**::*::.*.:*::. *::*:     .:

Arabidopsis         PNMDENA-------SKII-------------------------------------SAGR-     374
Solanum_1           PAVSGDA-------GGII-------------------------------------SAGR-     357
Solanum_2           VPTLKLS-------DDTPKKEQPQIQKLVSEA---------------------TIAKKK-     414
Pisum               PAVNENA-------GGII-------------------------------------TAGQ-     352
Hordeum             PAVNEGA-------SGII-------------------------------------TAGR-     361
Zea                 PVVTEGG-------SGII-------------------------------------TAGR-     368
Oryza_1             NPVKEYI-------DKLAKEESTNLGKPEPAI--------------KSDPLPKVGKPPA-     384
Oryza_2             PVVNQGG-------SGII-------------------------------------TAGR-     371
Chlamydomonas_1     VVMNELGPVTKPGSGRRN--------GVAAGEWSV------------WKPA-TVLTTAE-     401
Chlamydomonas_2     IPEAIKAPATAGSSTPIVKPKEERVKKVTGKELGSRKKRRNDDGEEVEDVEVEVVSSGSS     375

Arabidopsis         -AKRSIAQPDDAGERFRQLKEQEKRSKKNKAVAKDTVELVEESQSESEE-----GSDDEE     428
Solanum_1           -AKRTTSQPEQSGERFRQLKEDEKKKKSTKALPTDKVELSASI-SDSED-----EQDDDT     410
Solanum_2           -DANPTSDKPRQGERFKQLKEQEARKRQQREEEKRRAEVAANYRKEDKSVGLETRNEIDS     473
Pisum               -AKRSASKPEKGGERFRQLKEEEKKKKLIKALPVEEVQPLASA-SASND-----GSDV-E     404
Hordeum             -AKRSGSQSGQGGERFKQLKEEENRRKAVKALGAGDSNGSTT--SEDED-----S--DDD     411
Zea                 -AKRSNAQPA--GERFRQLKEEENRRKFSKALAAGDSDASSSTYDMDDE-----E--SDE     418
Oryza_1             -SQEPEPSGPQRGERFRKLKEEESRRKVFLEKAEQTEQAGT-------QAGIVDGKQNSD     436
Oryza_2             -AKRTSAQPAQPGERFKQLKEEESKRKGNKALAAGDSDLSASTSE-DEE-----S--DDE     422
Chlamydomonas_1     -AAKARAEAEEAVERAREAAEEAAAAAAF-----DNA--SVSL-SVDDS--------TAA     444
Chlamydomonas_2     SSSGSNGASGRKGEKFRALKAREAAAKAASTVSAGAG-------------GSEEGKDNSA     422
                                 *: :

Arabidopsis         E----EAREGALASSTTSKPLPEVGQRRSKRSKRKRTV----------------------     462
Solanum_1           KPKDEEVLEEAYASSSSKEVPNYSGPRKSKRSKRKRAV----------------------     448
Solanum_2           INGDSASLSRNASTSE--NYRIVNGDSTSSEVKADERSQTGKISDDHNDGEDQHVSDIRK     531
Pisum               NNKEQEVTEESNTSKVSQEVQSFSRERRSKRSKRKPVA----------------------     442
Hordeum             TAEEGGPVEETFTTGNDKKLPTYSG-KKGKRSKRKRIVQ---------------------     449
Zea                 TIEEGGPVEE-ASTSSDKKLPNYSG-KKGKRSKRKRMVQ---------------------     455
Oryza_1             ASGDNI--DEQESHEN--EPIIANGNGGLSHS-TNEMIP---------NGSMKEVCFVLI     482
Oryza_2             TTEEGGPEER-YNSSSNKKLPNYSG-KKGKRSKRKRMVQ---------------------     459
Chlamydomonas_1     IAGTATMAVTAGATAA--AMDPSKVNRRCKRRRLTSLVQDGSTASAAVAGASA-------     495
Chlamydomonas_2     ------------------------------------------------------------     422
```

Fig. S4: Excerpt of a multiple sequence alignment of Alb3 homologs in the indicated plant species. The alignment was performed with ClustalOmega (http://www.ebi.ac.uk/Tools/msa/clustalo/). The phosphorylation site is marked in red with a star.

Supplemental Table S1: MS- phosphoproteomics studies used for the peptide library generation at the status of January 2012

publications used for phosphopeptide list	PMID	PhosPhat 3.0 entry (January 2012)
Engelsberger WE, et al. (2012) The Plant Journal	22060019	yes
Reiland S, et al. (2011) PNAS	21768351	yes
Kline KG, et al. (2010) PNAS	20733066	yes
Nakagami H, et al. (2010) Plant Physiology	20466843	yes
Chen Y, et al. (2010) The Plant Journal	20374526	yes
Umezawa, et al. (2009) PNAS	19805022	yes
Lohrig K, et al. (2009) Planta	19238429	no
Reiland S, et al. (2009) Plant Physiology	19376835	yes
Ito J, et al. (2009) Proteomics	19688752	yes
Wang Z, et al. (2009) Journal of Proteomics	19341826	yes
Li H, et al. (2009) Proteomics	19253305	yes
Jones A, et al. (2009) Journal of Proteomics	19245862	yes
Whiteman S, et al. (2008) Proteomics	18686298	yes
Sugiyama N, et al. (2008) Molecular Systems Biology	18463617	yes
de la Fuente van Bentem S, et al. (2008) Journal of Proteome Research	18433157	yes
Carroll A, et al. (2008) Molecular and Cellular Proteomics	17934214	yes
Nühse T, et al. (2007) The Plant Journal	17651370	yes
Niittylä T, et al. (2007) Molecular and Cellular Proteomics	17586839	yes
Benschop J, et .al. (2007) Molecular and Cellular Proteomics	17317660	yes
de la Fuente van Bentem S, et al. (2006) Nucleic Acids Research	16807317	yes

Wolschin F, et al. (2005) Plant Methods	16270910	yes
Nühse T, et al. (2004) The Plant Cell	15308754	yes

Supplemental Table S2: List of peptides included on the microarray ChloroPhos1.0

type	Peptide	AGI 1	AGI 2	AGI 3	AGI 4	AGI 5	AGI 6	AGI 7	AGI 8
blank-control	AA	AT3G52930.1							
peptide library member	AADESTGTIGKRLAS	AT1G29910.1	AT1G29920.1	AT1G29930.1					
peptide library member	AAEADLDTEEDLEQT	AT3G25690.1	AT3G25690.2	AT3G25690.3					
peptide library member	AAEESDGSVKEDDDD	AT3G59980.1							
peptide library member	AAIEKLPTYSRLRTT	AT1G16080.1							
peptide library member	AAIIRIDSPGGDALA	AT4G25650.1	AT4G25650.2						
peptide library member	AAKVEQITAALQTGT	AT5G19390.1	AT5G19390.2	AT5G19390.3	AT5G19390.4				
peptide library member	AAKVKKITAELQAAS	AT2G47390.1							
peptide library member	AALNIVPTSTGAAKA	AT2G32040.1							
peptide library member	AALQTGTSSDKKAFD	AT1G74730.1							
peptide library member	AALSSSVTTRSYHSG	AT2G01140.1							
peptide library member	AAPDGATATKPKPPP	AT5G08050.1							
peptide library member	AASEVFGTGRITMRK	AT5G14740.1	AT5G14740.2	AT5G14740.3	AT5G14740.4	AT5G14740.5			
peptide library member	AASEVLGSGRVTMRK	AT3G03630.1							
peptide library member	AASFHVMSKSVDNVL	AT5G26742.1	AT5G26742.2						

peptide library member	AATIAVFTGGLKVKA	AT5G63570.1			
peptide library member	AAYGKWASEIAARLQ	AT5G64040.1	AT5G64040.2		
peptide library member	ACFIPTKSDANVVTF	AT5G20250.1	AT5G20250.2	AT5G20250.3	AT5G20250.4
peptide library member	ADDSDIESPRDTNGP	AT5G01530.1			
peptide library member	ADTSPDQSKEGSDSA	AT1G29930.1			
peptide library member	AEGDTSNTDVEGDRD	AT1G29910.1	AT1G29920.1		
peptide library member	AEGLGLTLSSLEKLK	AT4G29060.1	AT4G29060.2		
peptide library member	AEHKNKASPETVADF	AT3G52140.1	AT3G52140.2	AT3G52140.3	AT3G52140.4
peptide library member	AEKSGFSLSTIERLG	AT3G57610.1			
peptide library member	AELQAASSSDSKSFD	AT4G14870.1			
peptide library member	AENSGAITPRKTVLV	AT1G03130.1			
peptide library member	AFKSLGLSDHDEYDL	AT4G30910.1			
peptide library member	AFLVKAGSGVATLGL	AT4G22890.1	AT4G22890.2	AT4G22890.3	
peptide library member	AFTVQFGSCKFPENF	AT1G19870.1			
peptide library member	AGDEKKESPIFRLTG	AT1G29910.1	AT1G29920.1	AT1G29930.1	AT2G34420.1
peptide library member	AGDVIGTRTEAADAK	AT4G10340.1			

peptide library member	AGKAVKLSPAASEVL	AT1G19870.1		
peptide library member	AGKAVNLSPAASEVL	AT4G17090.1		
peptide library member	AGKQILSSTQRLVLP	AT5G14260.1	AT5G14260.2	AT5G14260.3
peptide library member	AGLGAGLTSLDKKKQ	AT2G21280.1		
peptide library member	AGQEFGTTTGRPRRC	AT5G05730.1		
peptide library member	AIEQRRDTAGSESES	AT3G52930.1		
peptide library member	AIRAEKTESSSAAPA	AT5G64040.1	AT5G64040.2	
peptide library member	AITPLYCSSKATAHT	AT4G02510.1		
peptide library member	AKATTEQSGPVGGDN	AT5G61210.1		
peptide library member	AKIEEDVTSEVEMAS	AT1G48620.1		
peptide library member	AKPKGPSGSPWYGSD	AT5G20720.1	AT5G20720.2	AT5G20720.3
peptide library member	AKSKAVSETSDELAK	AT1G68830.1		
peptide library member	AKVQEHNSPRSSPDL	AT1G15820.1		
peptide library member	ALERYDSSAFGQVVA	AT4G04640.1		
peptide library member	ALLADPSLSPRKRVA	AT5G40200.1		
peptide library member	ALLGEGATVVLEGQK	AT1G12900.1	AT1G12900.3	AT1G12900.4
protein-control	alphacasein	AT1G73990.1		

peptide library member	ALRVVNPSPYMGYLQ	AT2G37660.1	
peptide library member	ANAAYIGTPGKGILA	AT1G19870.1	
peptide library member	ANFARAFTVQFGSCK	AT3G20550.1	
peptide library member	ANISNNMSDRIDGQI	AT5G23060.1	
peptide library member	ANLPKHNSVDLKSSK	AT4G19490.1	AT4G19490.2
peptide library member	APPPPPPTNVESGGE	AT4G28750.1	
peptide library member	AQIIYSKYAGTEVEF	AT3G18890.1	
peptide library member	AQRNALASALRLQRK	AT5G04360.1	
peptide library member	AQRVGRKTLIVAAAA	AT3G60750.1	AT3G60750.2
peptide library member	ASDLKKSLSMVYNRK	ATCG00710.1	
peptide library member	ASEANPSPSRRSRGR	AT2G47400.1	
peptide library member	ASHLLKYDSTLGIFD	AT3G47070.1	
peptide library member	ASKPEGTGTPTKDFK	AT2G46820.1	AT2G46820.2
peptide library member	ASQAQQGTKDRKWQR	AT1G06690.1	
peptide library member	ASRHDEGSNARGGSE	AT3G14440.1	
peptide library member	ASRSFGTRSGTKFLP	AT3G17040.1	
peptide library member	ASSQSLSSILNNPHG	AT1G59870.1	
peptide library member	ASSSSKDSPAAAAAP	AT5G14740.4	

peptide library member	ASSTRKSDSLSPGPT	ATCG00490.1		
peptide library member	ATIVNAKSAIGSLRK	AT1G78510.1		
peptide library member	ATLESDWSAKFAAYE	AT5G64040.1	AT5G64040.2	
peptide library member	ATQTVEDSSRSGPRS	AT2G35410.1		
peptide library member	ATSEGEISEKVEKSI	AT1G55490.1	AT1G55490.2	
peptide library member	ATSTKGGSGGPKEEK	AT3G13470.1		
peptide library member	ATTTEAETTELPEIV	AT5G56500.1	AT5G56500.2	
peptide library member	AVGVSNYSEKRLRDA	AT5G66190.1	AT5G66190.2	
peptide library member	AVKFEHGSASYACRF	AT1G15500.1		
peptide library member	AVVDRSSSGVFSPQK	AT5G63420.1		
peptide library member	AVVEDDVYGNQLMSK	AT4G24770.1		
protein-control	betacasein	AT3G01500.1	AT3G01500.2	AT3G01500.3
peptide library member	CERAVNVSLANLLTY	AT2G38040.1	AT2G38040.2	
peptide library member	CEVWKEITFNFPTID	AT2G38040.1	AT2G38040.2	
peptide library member	CGRRQFPSLAKTVCK	AT1G73110.1		
peptide library member	CKFPENFTGCQDLAK	AT4G26670.1		

peptide library member	CSVAEKETSADEETS	AT4G12610.1	AT4G12610.2		
peptide library member	CTLLRLASKVDAIKA	AT2G38140.1			
peptide library member	CTLLRLASKVDAIKD	AT5G63420.1			
peptide library member	CTLLRLASKVDAIKE	AT5G61210.1			
peptide library member	CVKRLVYTNDGGEIV	AT3G47070.1			
peptide library member	DAPSGETTSQLSEKS	AT5G26742.1	AT5G26742.2		
peptide library member	DASDSETKSSPKRVR	AT1G52510.1	AT1G52510.2		
peptide library member	DASEGDVSEGDESEG	AT5G19390.1	AT5G19390.2	AT5G19390.3	AT5G19390.4
peptide library member	DDDDDEDSSESGKSE	AT5G49910.1			
peptide library member	DDEDSSESGKSEMVN	AT1G12800.1			
peptide library member	DEAGGMETLGKVLLR	AT1G48860.1	AT1G48860.2	AT2G45300.1	
peptide library member	DEATDNDSSKALVTI	AT5G45930.1			
peptide library member	DEEEGNVSDRGDEDE	AT4G02510.1			
peptide library member	DEKIKIDIDESLFSN	AT3G04790.1			
peptide library member	DELADASDSETKSSP	AT5G42080.1	AT5G42080.2	AT5G42080.3	
peptide library member	DESDNKHTLNPSKRT	AT3G63190.1			

peptide library member	DFVLGFMTKQDQFYE	AT4G02510.1		
peptide library member	DGEELAISKLSLPQR	AT1G17745.1	AT1G17745.2	
peptide library member	DKGKEYGSTIKSGKL	AT4G26670.1		
peptide library member	DNEVEPVTDDDNDRA	AT3G18890.1		
peptide library member	DNGGDVIDADFTDSN	AT5G19390.1	AT5G19390.2	AT5G19390.3
peptide library member	DNKKKSTSSLDGLKL	AT4G22890.1	AT4G22890.2	AT4G22890.3
peptide library member	DPGCTRKTFPDYFQV	AT1G19870.1		
peptide library member	DPLESMDSGILVTEK	AT2G05120.1	AT2G05120.2	
peptide library member	DQIGDDDSGEFEPVS	AT4G02510.1		
peptide library member	DQIGKLLSSGELYDI	AT3G26840.1		
peptide library member	DRTFGVLTKIDLMDK	AT5G23890.1		
peptide library member	DSCLRIISSAISPST	AT5G24490.1		
peptide library member	DSDEDVDTEDEGEEK	AT1G12900.1	AT1G12900.3	AT1G12900.4
peptide library member	DSDEYNPTLPKPRIL	AT4G24770.1		
peptide library member	DSDSNLNTDGDEATD	AT4G32260.1		
peptide library member	DSDTDKSSTVAKTVT	AT5G40200.1		

peptide library member	DSKISEESGKISVDK	AT3G19170.1	AT3G19170.2
peptide library member	DSNVLPYCSINKAEK	AT1G33810.1	
peptide library member	DSPDTTNTIKDHSLE	AT4G12610.1	AT4G12610.2
peptide library member	DSPVTPATQNRNNFI	AT1G59870.1	
peptide library member	DSSSEAVSGNSDKVG	AT4G14870.1	
peptide library member	DTKMFDKYKIMGGVP	AT4G28750.1	
peptide library member	DTTKELESSKSPVPE	AT2G20260.1	
peptide library member	DVEDSTDSSVGEEEE	AT2G48120.1	AT2G48120.2
peptide library member	DVKPSGDSALSVDGK	AT1G07320.1	
peptide library member	DVSEGDESEGDVSEG	AT1G19870.1	
peptide library member	EALASLESQKEETIK	AT5G65220.1	
peptide library member	EASANEASLPQSPEP	AT1G50900.1	
peptide library member	EASPSPNSSKIKFQK	AT4G36970.1	
peptide library member	EDDSDKFSNAKLKVT	AT3G20550.1	
peptide library member	EDEEEEASRKSRLGL	AT4G28730.1	
peptide library member	EDIFSSGSRRTQSVN	AT1G09795.1	
peptide library member	EDKEVEISAIGAEIK	AT4G01150.1	AT4G01150.2

peptide library member	EDPAPASSSSKDSPA	AT4G02510.1		
peptide library member	EDTPPATASSDSSST	AT3G12780.1		
peptide library member	EEDATTGSKGEGLDP	AT1G08550.1	AT1G08550.2	
peptide library member	EEDDDEDETEGSEEA	AT5G65620.1		
peptide library member	EEDDLIGTELQGPSA	AT1G19870.1		
peptide library member	EEEAAEEAAEAAKSA	AT1G67090.1		
peptide library member	EEIRDLILGYSTQKA	AT1G19870.1		
peptide library member	EEKAEMTTAMQSPVV	AT1G19870.1		
peptide library member	EEPSFELSGKLAEET	AT1G68830.1		
peptide library member	EESIRKTVTENTVVI	ATCG00490.1		
peptide library member	EETPRWSQLLSNLGL	AT3G46780.1		
peptide library member	EETSSIDTNELITDL	AT1G80300.1		
peptide library member	EEVEEKFTSESDSIA	AT4G02510.1		
peptide library member	EFAKKLASLADLYVN	ATCG01130.1		
peptide library member	EFPAPDPSVLVQNFN	AT5G35630.1	AT5G35630.2	AT5G35630.3
peptide library member	EFRGREPSPEPLLRH	AT4G02510.1		
peptide library member	EGKTDIDSPDTTNTI	AT4G01150.1	AT4G01150.2	
peptide library member	EHGNSPGYYDGRYWT	AT2G32040.1		

peptide library member	EIDVKDATSMGTVED	AT1G19870.1				
peptide library member	EIGTGEATEQEEGKE	AT3G17040.1	AT3G17040.2			
peptide library member	EISDGRKTVWWNRWI	AT4G36970.1				
peptide library member	EITFNFPTIDKLDGQ	AT5G17670.1				
peptide library member	EKEAEAASLAEDAQQ	AT2G43090.1				
peptide library member	EKEMERASSVKIPVV	AT3G20550.1				
peptide library member	EKFTSESDSIADSSK	AT4G02510.1				
peptide library member	EKKLSFFSEPQQEEK	AT5G61210.1				
peptide library member	EKPVEDPSELPKWNY	AT1G19870.1				
peptide library member	ELESDKATEEGGGKL	AT3G01500.1	AT3G01500.2	AT3G01500.3	AT5G14740.1	AT5G14740.2
peptide library member	ELLKTRASSEETSSI	AT1G15500.1				
peptide library member	ELPRRRDSEESLLLD	AT5G18820.1				
peptide library member	ELTSSTGSNKAMTLS	AT1G19870.1				
peptide library member	ELYQRALSIDANTES	AT4G23890.1				
empty	empty	AT3G63490.1	AT3G63490.2			
peptide library member	EMTTAMQSPVVSRRD	AT4G02510.1				
peptide library member	ENCAKAVYTPELKYV	AT4G14870.1				
peptide library member	ENFGCGSSREHAPVC	AT3G03710.1				

peptide library member	EPNVEEDSVARMRAV	AT4G02510.1	
peptide library member	EPSGEGVTRVDGSES	AT4G31390.1	AT4G31390.2
peptide library member	EPSLADMTNPFGGER	AT5G36880.1	AT5G36880.2
peptide library member	EQKSGRRTSFGYDQE	AT5G53170.1	
peptide library member	EREAVNVSLANLLTY	AT4G32520.1	AT4G32520.2
peptide library member	EREMERASSVKIPVV	AT1G09340.1	
peptide library member	ESAGDGTTTAIILAR	AT4G32260.1	
peptide library member	ESALLKSPSPDNNNV	AT1G19870.1	
peptide library member	ESDDSNLSFKVPEDG	AT2G35410.1	
peptide library member	ESDSIADSSKLESVD	AT1G19870.1	
peptide library member	ESESEATPSPAEESG	AT2G01140.1	
peptide library member	ESETDKDSQKQQPAG	AT1G59870.1	
peptide library member	ESKIENGSVDVDVKQ	ATCG00280.1	
peptide library member	ESKLDLTDTIKDGAR	AT3G16620.1	
peptide library member	ESMSQLPSGAGKISQ	AT5G42270.1	
peptide library member	ESNRLVSDTEVSELE	AT1G42970.1	
peptide library member	ESTGIVDYDMLEKTA	ATCG00490.1	

peptide library member	ETESLLQSKGVNWTS	AT4G25650.1	AT4G25650.2		
peptide library member	ETIKALDSQIAALSE	AT2G45290.1			
peptide library member	ETQNVKISPQADFVN	AT1G12900.1	AT1G12900.3	AT1G12900.4	
peptide library member	ETSADEETSQEEKTE	AT5G11880.1			
peptide library member	ETTPASRTRETLESA	AT1G06680.1	AT1G06680.2		
peptide library member	ETVADFTLTMLKRRV	AT1G78915.1	AT1G78915.2	AT1G78915.3	
peptide library member	EVAMGRMSRDSAAEA	AT5G08540.1			
peptide library member	EVIFGGETMRFWDLR	AT5G05200.1			
peptide library member	EVLSSVSPTESRSDT	AT1G64510.1			
peptide library member	EVPETGVTFGDVAGA	AT5G46110.1	AT5G46110.2	AT5G46110.3	AT5G46110.4
peptide library member	EVVVLNDSGGVKNAS	AT4G28730.1			
peptide library member	EYKLTYYTPEYETKD	AT1G59870.1			
peptide library member	FAASKWLSHFIYKTF	AT5G48300.1			
peptide library member	FAAYEKKYPEEAAEL	AT2G37660.1			
peptide library member	FDADVKPSGDSALSV	AT5G64300.1			
peptide library member	FDHCFKKSSDGFLYC	AT4G14070.1			
peptide library member	FEDNFDATSNLNVMV	AT5G08280.1			

peptide library member	FEGKSGRSFDIDFDE	AT4G22890.1	AT4G22890.2	AT4G22890.3
peptide library member	FEKTAESSSDKEEDS	AT3G10840.1		
peptide library member	FERMGATYIKLGQFI	AT4G13670.1		
peptide library member	FFEGGFGSDDDPTSP	AT3G46780.1		
peptide library member	FGNKISTQTGIGTGI	AT1G32060.1		
peptide library member	FGSRMEESIRKTVTE	AT1G54520.1		
peptide library member	FHVGTKMSNELAVPF	AT5G13550.1		
peptide library member	FIQAHRETDADITVA	AT2G31170.1		
peptide library member	FKALDLASKPEGTGT	AT3G25690.1	AT3G25690.2	AT3G25690.3
peptide library member	FKGDVVEKIESESES	AT5G53170.1		
peptide library member	FLESSSFSGDAALRS	AT1G10760.1		
peptide library member	FLETLDGSCRTPIAG	AT5G26570.1	AT5G26570.2	
peptide library member	FLLPAKATTEQSGPV	AT4G05180.1		
peptide library member	FLNQPKQTLSTLSLS	AT2G28800.1	AT2G28800.4	
peptide library member	FMDEDVETDKDEAST	AT3G60750.1	AT3G60750.2	
peptide library member	FNKAKDISSGLSWNK	AT3G09050.1		
peptide library member	FPGSNNGTGLFQTIV	ATCG00490.1		

peptide library member	FSSRSRSSSSSSSQS	AT1G06950.1	
peptide library member	FTRRYGGSNNNSSSS	AT2G32040.1	
peptide library member	FTTLSSLTDGGAPIS	AT1G15500.1	
peptide library member	FVDDPNLSCEPALKK	AT5G54600.1	
peptide library member	FVGGEETKSGGEEAE	AT2G36390.1	
peptide library member	FVKSGGNSHLKLEID	AT2G37220.1	
peptide library member	FVRLEASPSHVNLIL	AT4G02510.1	
peptide library member	FYIQPLSPTEAAARA	AT5G40200.1	
peptide library member	GALASSTTSKPLPEV	AT2G37220.1	
peptide library member	GDATRNLSQQCLNAL	ATCG00680.1	
peptide library member	GDGTSDSDSDPDPPK	AT4G34190.1	
peptide library member	GDHIHAGTVVGKLEG	AT1G54520.1	
peptide library member	GDLEAQDSKAKYLQK	AT4G12610.1	AT4G12610.2
peptide library member	GDRDTTSSIRTQPPP	AT5G13550.1	
peptide library member	GETTSQLSEKSTPTG	AT4G39040.1	AT4G39040.2
peptide library member	GEVTKIFTHNSTIVI	AT3G55800.1	
peptide library member	GFNRLDDSAEFFTSD	AT3G52930.1	
peptide library member	GFSRGPRSSFGSSGS	AT4G38970.1	AT4G38970.2

peptide library member	GGEVELESDKATEEG	AT2G39730.1	AT2G39730.2	AT2G39730.3
peptide library member	GGEVKEASANEASLP	AT3G60750.1	AT3G60750.2	
peptide library member	GGGSGAGSGNRVYVG	AT5G19220.1		
peptide library member	GGLFRAGSMDNGDGI	AT1G74040.1		
peptide library member	GGNRAASVSIRSEQS	AT1G74070.1		
peptide library member	GGNSFSSRSRSSSSS	AT1G67090.1	AT1G67090.2	
peptide library member	GGRGRKKSSGGDEEE	AT3G48500.1	AT3G48500.2	
process-control	GGSGGGSDYKDDDDK	AT2G38040.1	AT2G38040.2	
peptide library member	GGSNNNSSSSNALLK	AT1G11860.1	AT1G11860.2	AT1G11860.3
peptide library member	GIEDSEASEEVSEIG	AT2G26540.1		
peptide library member	GIFTNVTSPTAKAKL	AT3G60750.1	AT3G60750.2	
peptide library member	GILAADESTGTIGKR	ATCG00280.1		
peptide library member	GILAMDESNATCGKR	AT1G74730.1		
peptide library member	GINPIMMSAGELESG	AT5G52840.1		
peptide library member	GINSFGASAPAPLLY	AT3G25690.1	AT3G25690.2	AT3G25690.3

peptide library member	GIRSRVGSNVQLKDT	AT5G26742.1	AT5G26742.2				
peptide library member	GIVLGKLSGRHALKD	AT2G28000.1					
peptide library member	GIVVRDPSKPPPKTK	AT1G71500.1					
peptide library member	GKKKFETLSYLPDLT	AT2G37220.1					
peptide library member	GKKLSELSDDEDFDE	AT1G65410.1					
peptide library member	GKPYEGSITKMPFVA	AT5G36700.1	AT5G36700.2	AT5G36700.4	AT5G36790.1	AT5G36790.2	AT5G36790.3
peptide library member	GKRSSVLYPASLKAG	AT4G19170.1					
peptide library member	GKSIGINSFGASAPA	ATCG00120.1					
peptide library member	GKYLMRSPTGEVIFG	AT1G54520.1					
peptide library member	GLGLTLSSLEKLKVF	AT1G48620.1					
peptide library member	GLIAESKTEIPAATP	AT1G19870.1					
peptide library member	GLNSVAASFHVMSKS	AT5G18660.1					
peptide library member	GLSDHDEYDLDGDNN	ATCG01130.1					
peptide library member	GNDDLIGSMIADAID	AT5G13630.1	AT5G13630.2				
peptide library member	GNVRCEATEVSSSSS	AT4G02510.1					
peptide library member	GPRSSFGSSGSGYGG	AT3G20550.1					
peptide library member	GPSGTGKSTILKIMA	AT1G06680.1	AT1G06680.2				

peptide library member	GQNGGCKTLLVLSGV	AT2G30790.1		
peptide library member	GRCSRYVYAAIGDPM	AT3G25690.1	AT3G25690.2	AT3G25690.3
peptide library member	GRGKISASESRLIES	AT2G41220.1		
peptide library member	GRIGGNSFSSRSRSS	AT5G01590.1		
peptide library member	GRPRKDGTSPTVKPA	AT5G23060.1		
peptide library member	GRRTSFGYDQEARES	AT1G19870.1		
peptide library member	GRYYAAESMLILDPE	AT1G14810.1		
peptide library member	GSAQRKPSFFEPISK	AT3G27925.1		
peptide library member	GSDKGILSDVELLKE	AT3G01500.1	AT3G01500.2	AT3G01500.3
peptide library member	GSESEEETEEMIFGS	AT3G59400.1		
peptide library member	GSNARGGSEEPNVEE	AT4G34240.1		
peptide library member	GSPEEFLSQVNYLLG	AT2G37220.1		
peptide library member	GSPEQFLSQVNYLLG	AT2G17972.1		
peptide library member	GSPGRLSSSMNKQRG	AT4G25130.1		
peptide library member	GSQVLVLSDRSDNPE	AT4G14970.1		
peptide library member	GTDEPGPYKQIAEQF	AT1G12900.1	AT1G12900.3	AT1G12900.4
peptide library member	GTDSYNFSFAQVLSP	AT1G32470.1		
peptide library member	GTELQGPSAADAAKI	AT5G13550.1		

peptide library member	GTIVVDNSSAFRMVD	AT5G51820.1		
peptide library member	GTKVSNGSDLYRILD	AT5G61210.1		
peptide library member	GVGAAIEYAVLHLKV	AT4G02510.1		
peptide library member	GVGADKRVFKTNYSF	AT1G19870.1		
peptide library member	GVLYRSFSGDADLRY	AT4G24620.1		
peptide library member	GYGGGGGSGAGSGNR	AT1G73060.1		
peptide library member	HFLAPNSTIINLKQQ	AT4G26670.1		
peptide library member	HGIVHNPSYEDVCTG	AT4G02510.1		
protein-control	Histon 1	AT3G01500.1	AT3G01500.2	AT3G01500.3
protein-control	Histon 2	AT5G13110.1		
protein-control	Histon 3	AT4G28750.1		
protein-control	Histon 4	AT2G34430.1		
process-control	H-KPKS-Cys(ATTO_550-SPQ-NH3	AT1G59870.1		
peptide library member	HLKNIQKTVNVVVSL	AT1G19870.1		
peptide library member	HLLKYDSTLGIFDAD	ATCG00280.1		
peptide library member	HLLPAFSISRCFSSV	AT4G01150.1	AT4G01150.2	
peptide library member	HLSFTRRYGGSNNNS	AT3G23790.1		
peptide library member	HNPGGPEYDWGIKFN	AT5G08050.1		

peptide library member	HNSVDLKSSKPNPFD	AT5G61410.1	AT5G61410.2			
peptide library member	HSDEANISNNMSDRI	AT5G53460.1	AT5G53460.2	AT5G53460.3		
peptide library member	HSLKRKVSNPSFIAA	AT5G26742.1	AT5G26742.2	AT5G26742.3		
peptide library member	IAEGNCGSPRSIKVY	AT1G69830.1				
peptide library member	IDFPPLPSSISSYKG	AT4G02510.1				
peptide library member	IDGNLDSDSNLNTDG	AT1G16720.1				
peptide library member	IDGQIVTDSDEDVDT	AT1G12900.1	AT1G12900.2	AT1G12900.3	AT1G12900.4	AT3G26650.1
peptide library member	IDHRTEVSPVIVESK	AT2G45770.1	AT2G45770.2			
peptide library member	IDRDFEPVLSMTPLN	AT4G30950.1				
peptide library member	IDTNELITDLKEKWD	AT5G01600.1				
peptide library member	IEEAAMRSNLIQQIV	ATCG00490.1				
peptide library member	IERLGLLTKAEEFGV	ATCG00490.1				
peptide library member	IEVDGGVTPANAYKV	AT5G02250.1				
peptide library member	IFEALGLSSEVIQKC	AT3G25690.1	AT3G25690.2	AT3G25690.3		
peptide library member	IGGRSSSSSRAPSRE	AT1G15210.1	AT1G59870.1			

peptide library member	IGKSVGVSSMNKSPV	AT1G80380.1	AT1G80380.2	AT1G80380.4
peptide library member	IGVDDVSSLSPKPEA	AT2G32040.1		
peptide library member	IHRFHDRSQIVIPRA	AT3G01500.1	AT3G01500.2	AT3G01500.3
peptide library member	IIKGTMTTTHSYTGD	AT2G30790.1		
peptide library member	IMSGKLKSGSEIKDA	AT4G34240.1	AT4G34240.2	
peptide library member	IMTVCHVYDKEENYI	AT1G19870.1		
peptide library member	IPITSHASLARQRFA	AT3G60750.1	AT3G60750.2	
peptide library member	IPPAYTKTFQGPPHG	AT3G27740.1		
peptide library member	IREACKWSPELAAAC	AT1G42970.1		
peptide library member	IRRYMDLTAHYQIKA	AT4G02510.1		
peptide library member	IRTQQQASSPGEGLN	AT5G20720.1	AT5G20720.2	AT5G20720.3
peptide library member	ISGYCEQTDIHSPQV	AT3G52930.1		
peptide library member	ISSTLQHYNNRIYIR	AT3G46780.1		
peptide library member	ISVAEGDTSNTDVEG	AT5G63420.1		
peptide library member	ITAALQTGTSSDKKA	AT1G58200.1	AT1G58200.2	
peptide library member	ITPTDKKSITDYGSP	ATCG00900.1	ATCG01240.1	

peptide library member	ITTKDFASKLIDALK	AT3G52230.1			
peptide library member	IVESKGTETEEDDLI	ATCG00490.1			
peptide library member	IVGGKGKSIGINSFG	AT3G25690.1	AT3G25690.2	AT3G25690.3	
peptide library member	IVGIDLISDVSCKSP	AT3G46780.1			
peptide library member	IVKGTMTTTHSYTGD	AT1G59870.1			
peptide library member	IVSSREFSFGGKEVD	AT3G10050.1			
peptide library member	IYSKYAGTEVEFNDV	AT5G08540.1			
peptide library member	KAAQEALYVRCKANS	AT4G14070.1			
peptide library member	KADAVGVTVDGLFNK	AT5G23890.1			
peptide library member	KAEGKENSRDDDELA	AT3G11050.1			
peptide library member	KAEKDEVSDDEATIE	AT1G67700.1	AT1G67700.2	AT1G67700.3	
peptide library member	KARRVGGSTHQVPIE	AT1G15210.1			
peptide library member	KDDDEDDQSSDGHED	AT4G02510.1			
peptide library member	KDDENVNSQPFMRWR	AT1G19870.1			
peptide library member	KDDSSVQSSPSRSFY	AT5G42270.1			
peptide library member	KDISSGLSWNKLGSQ	AT5G19390.1	AT5G19390.2	AT5G19390.3	AT5G19390.4
peptide library member	KDPMRRSLSTADGNR	AT4G04770.1			
peptide library member	KEAVVLYSVGVHTAG	AT4G36970.1			

peptide library member	KEEDSNEEDDSNTTS	AT1G32060.1					
peptide library member	KEKEVKPSSPFLESS	AT5G14740.1	AT5G14740.2	AT5G14740.3	AT5G14740.4	AT5G14740.5	
peptide library member	KELESSKSPVPESTD	AT4G02510.1					
peptide library member	KEMELVPTTPFVSLA	AT5G36880.2					
peptide library member	KESFGKLSSLNPGSD	AT1G67090.1	AT1G67090.2				
peptide library member	KETEMESVSAKKGMV	AT1G08640.1					
peptide library member	KEVDQEPSGEGVTRV	AT4G08510.1					
peptide library member	KFEELTSSTGSNKAM	AT4G10340.1					
peptide library member	KFQEVPETGVTFGDV	AT4G04640.1					
peptide library member	KGFVADDSDIESPRD	AT3G12780.1					
peptide library member	KGISAGHSRNCYRGL	ATCG00490.1					
peptide library member	KGLMVAGSPFSTGVL	AT1G70610.1					
peptide library member	KHADFPGSNNGTGLF	AT3G46780.1					
peptide library member	KICLPAKSKVLAESE	AT3G25690.1	AT3G25690.2	AT3G25690.3			
peptide library member	KINADAETLEVANKF	AT1G67090.1	AT5G38410.1	AT5G38410.2	AT5G38410.3	AT5G38420.1	AT5G38430.1
peptide library member	KISGSLGSRILPATQ	AT4G01690.1					
peptide library member	KKFETLSYLPDLTDS	AT5G08540.1					

peptide library member	KKGVTFGSFKVSKDI	AT3G14210.1		
peptide library member	KKLSLAQTQSRHAFY	AT2G01140.1		
peptide library member	KKPAPAKSKAVSETS	AT1G79040.1		
peptide library member	KKSLSMVYNRKRQAK	AT3G01480.1	AT3G01480.2	
peptide library member	KKSVGDLTSADLKGK	AT3G52930.1		
peptide library member	KLEGDRESTLGFVDL	AT5G64050.1		
peptide library member	KLFRTDISSNIKYGC	AT5G17230.1	AT5G17230.2	AT5G17230.3
peptide library member	KLGSQFATAIQNASE	AT3G63130.1	AT3G63130.2	
peptide library member	KLKKWGKSKDDSSVQ	AT1G19870.1		
peptide library member	KLPLFGCTDSAQVLK	AT1G06950.1		
peptide library member	KMLIKPNSTDPLKLG	AT5G61210.1		
peptide library member	KMVEFEKTAESSSDK	AT2G39730.1	AT2G39730.2	AT2G39730.3
peptide library member	KNDIKLLYSLGASKF	AT4G25650.1	AT4G25650.2	
peptide library member	KNKASPETVADFTLT	AT3G63160.1		
peptide library member	KPFGINGSMDLRDGV	AT3G47070.1		
peptide library member	KPLEDITDSLKIAGV	AT1G65260.1		
peptide library member	KPWSLSFSFGRALQQ	AT5G08050.1		

peptide library member	KQLPPVYTGKWATAS	AT3G60750.1	AT3G60750.2	
peptide library member	KQLRSSSYDLDVKKP	AT5G14910.1		
peptide library member	KRGPQEESEVEVSKD	AT2G35500.1		
peptide library member	KRKVSNPSFIAAQSK	AT1G56190.1	AT1G56190.2	
peptide library member	KRLANAVSSGDLEAQ	AT5G52840.1		
peptide library member	KRTTSEPSLADMTNP	AT4G14970.1		
peptide library member	KRVQLAETYLSQAAL	AT4G02510.1		
peptide library member	KSDANVVTFRRWFNK	AT1G19870.1		
peptide library member	KSDSDDATVPPPSGA	AT1G19870.1		
peptide library member	KSEGGFGGLGSLFKK	AT3G06350.1		
peptide library member	KSFADNATALKTQLD	ATCG00860.1	ATCG01280.1	
peptide library member	KSGFSLSTIERLGLL	AT3G48500.1	AT3G48500.2	
peptide library member	KSHWSRHTPEGATLE	AT2G28000.1		
peptide library member	KSPNLSSTHLLPLSK	AT4G02510.1		
peptide library member	KSVAQASLSALKQLI	AT4G05180.1		
peptide library member	KSVGDLNSVDLKGKK	AT4G30620.1		
peptide library member	KTEIPAATPSDPQLK	AT2G21330.1	AT2G21330.2	AT2G21330.3

peptide library member	KTVNVVVSLVNLCRS	AT4G17740.1	AT4G17740.2		
peptide library member	KVDVDDKSDNVIEEE	AT5G65780.2			
peptide library member	KVEPEESESDDVIIV	AT5G18660.1			
peptide library member	KVSKKITSSPKQEIG	ATCG00040.1			
peptide library member	KVVIANRTYERALEL	AT1G19870.1			
peptide library member	KWYFELGTSMKKFTI	AT1G31330.1			
peptide library member	KYEGKKLSELSDDED	AT3G20390.1			
peptide library member	LADCVGLTLGPRGRN	AT3G17040.2			
peptide library member	LAELEKASSGIEAHS	AT3G52930.1			
peptide library member	LALKDRFYIQPLSPT	ATCG00480.1			
peptide library member	LAMKERMSDLAQSLG	AT4G09160.1			
peptide library member	LASRTAAYYQQGARF	AT4G26900.1			
peptide library member	LCELPGSGSNSPKIG	AT2G43750.1	AT2G43750.2	AT3G59760.1	AT3G59760.3
peptide library member	LDCSESPSEAGRKMG	AT2G25140.1			
peptide library member	LDPETGEYSEEKTPS	AT3G61440.1	AT3G61440.2	AT3G61440.3	
peptide library member	LDSKIPISSIIGSLA	AT4G11960.1			
peptide library member	LDTLEKKSDAEGAEP	AT1G54580.1	AT1G54630.1	AT1G54630.2	

peptide library member	LESSLKLYAPESAPA	AT2G35410.1			
peptide library member	LFRMSSRSPPFASLS	AT1G31330.1			
peptide library member	LFVDRSSSGVFSPQK	ATCG00710.1			
peptide library member	LGDGAAESLHVKDYK	AT2G32040.1			
peptide library member	LGQEINVTCEVQQLL	AT5G36880.2			
peptide library member	LGSFKEETNKISDLS	AT5G08050.1			
peptide library member	LHPKLPATQKPMEGK	AT3G04790.1			
peptide library member	LIAVVFPSFGERYLS	AT3G25690.1	AT3G25690.2	AT3G25690.3	
peptide library member	LISLDMGSLLAGAKF	AT3G63410.1			
peptide library member	LIVTIHASFGERYLS	AT3G56690.1			
peptide library member	LKASTDQSGQVGGEE	AT5G61210.1			
peptide library member	LKEADEITAATKFAA	AT3G23400.1			
peptide library member	LKFSLRSASESEDGD	AT4G13430.1			
peptide library member	LKLYAPESAPALALN	AT4G08390.1	AT4G08390.2	AT4G08390.3	AT4G08390.4
peptide library member	LLKPLNSEYGKVAPG	AT2G41220.1			
peptide library member	LLLDSRISVAEGDTS	AT5G04140.1	AT5G04140.2		
peptide library member	LLLHRKMSSNSLRHV	AT4G17740.1	AT4G17740.2		

peptide library member	LLSLAEKSGFSLSTI	AT1G15210.1			
peptide library member	LLSSGELYDIVGIPT	AT5G01590.1			
peptide library member	LMLEYAGSERGQGDT	AT4G01690.1	AT4G01690.2		
peptide library member	LMLNGAITFPKGLGS	AT1G73060.1			
peptide library member	LNLMDGISRTDGVVV	AT2G45290.1			
peptide library member	LNPSKRTTSEPSLAD	AT3G60750.1	AT3G60750.2		
peptide library member	LNRGLVASVDDLERA	AT4G02510.1			
peptide library member	LNYVENRTSVPFEPV	AT2G39730.1			
peptide library member	LPDAGPPSPATHLRE	AT5G45930.1			
peptide library member	LPGKKVSAYIARLRN	AT2G47730.1			
peptide library member	LPGKKVSAYIARLRS	AT5G48220.1			
peptide library member	LPGSGSNSPKIGYIK	AT5G23060.1			
peptide library member	LPGVTGLSTEQRKRL	AT3G55800.1			
peptide library member	LPKFSKKSPKPETAG	AT5G45930.1			
peptide library member	LPKPQGQTVGSFRKG	AT1G72640.2			
peptide library member	LPSSISSYKGSSDDF	AT5G14740.1	AT5G14740.3	AT5G14740.4	AT5G14740.5
peptide library member	LPTYTPDSPGDATRN	AT4G08390.1	AT4G08390.2	AT4G08390.3	AT4G08390.4

peptide library member	LPTYTPESPGDATRN	AT5G26742.1	AT5G26742.2				
peptide library member	LPVEDIVSSREFSFG	AT3G47470.1					
peptide library member	LPVPEGCTDPVAENF	ATCG01130.1					
peptide library member	LQGRDQVTAEDVGIV	AT1G52220.1	AT1G52220.2	AT1G52220.3			
peptide library member	LQKVLDVYEARLAKS	AT5G13650.1	AT5G13650.2				
peptide library member	LQPFPSSATQRRLSP	AT3G09050.1					
peptide library member	LQSRLGTDSYNFSFA	AT1G31190.1					
peptide library member	LRATFDNSEYSKLID	AT2G32480.1					
peptide library member	LRKDPLESMDSGILV	AT1G76080.1					
peptide library member	LRPKDGSLSGDDDEF	AT4G38970.1	AT4G38970.2				
peptide library member	LRRMGNESYEDAIEA	AT1G32470.1					
peptide library member	LSASSSLSFVRSLVS	AT5G28500.1	AT5G28500.2				
peptide library member	LSEEAFKSLGLSDHD	AT5G24400.1					
peptide library member	LSFTSIGSSAKTSSF	AT3G46780.1					
peptide library member	LSIEKKLSFFSEPQQ	AT5G38410.1	AT5G38410.2	AT5G38410.3	AT5G38420.1		
peptide library member	LSKIVKKSVADILGQ	AT4G15530.1	AT4G15530.2	AT4G15530.3	AT4G15530.4	AT4G15530.5	AT4G15530.6

peptide library member	LSKNTSITYKNTKVN	AT1G17745.1	AT1G17745.2
peptide library member	LSKSGDGTSDSDSDP	AT5G66190.1	
peptide library member	LSNSFKSTTRLQTKA	AT5G19220.1	
peptide library member	LSPNVRITKVRPRNI	AT3G53130.1	
peptide library member	LSRSMSETVVFARMN	AT3G46780.1	
peptide library member	LSSRTAAYYQQGARF	AT5G59250.1	
peptide library member	LSSSASKSHLLPAFS	AT1G68830.1	
peptide library member	LSSSIFTQSKTHGFF	AT5G23040.1	
peptide library member	LSVSSIGTGSTKKSS	AT5G53170.1	
peptide library member	LSWNKLGSQFATAIQ	AT2G05120.1	AT2G05120.2
peptide library member	LSYLPDLSDVELAKE	AT4G26670.1	
peptide library member	LTARGGMTSHAAVVA	AT5G12860.1	AT5G12860.2
peptide library member	LTEDNRFSTVGSDSD	AT5G35170.1	AT5G35170.2
peptide library member	LTKISSVSPQRIFLK	AT1G50250.1	
peptide library member	LTQYNSASLNRHLAR	ATCG00710.1	
peptide library member	LTTLTRTLSSGKNDE	AT3G14550.1	
peptide library member	LTVWAKQTAFQLGKT	AT5G45930.1	

peptide library member	LVDDAYLSVKTAYED	AT1G59870.1				
peptide library member	LVKTVTETIDEISDG	AT5G19390.1	AT5G19390.2	AT5G19390.3	AT5G19390.4	
peptide library member	LVRNSSKTSLLPFRN	AT1G08540.1				
peptide library member	LVSDTEVSELETNDR	AT1G15210.1				
peptide library member	LYKFDLPYDAGKVLD	AT3G16000.1				
peptide library member	MAANDSSNAIDIDGN	AT5G61210.1				
peptide library member	MASLALSGSCSLAFP	AT5G14740.2				
peptide library member	MASLSLSSAHFSSTS	AT1G59870.1				
peptide library member	MASNSLLRSSSNFFL	AT5G11670.1				
peptide library member	MATQTVEDSSRSGPR	AT1G66670.1				
peptide library member	MATTVHLSSFSLFIQ	ATCG00350.1				
protein-control	MBP bovine	AT5G14740.1	AT5G14740.2	AT5G14740.3	AT5G14740.4	AT5G14740.5
peptide library member	MDSGILVTEKFYEVF	AT1G79850.1				
peptide library member	MDYNPNLPPLGGGGV	AT5G36880.2				
peptide library member	MEASLAALERPRGSA	AT5G18660.1				
peptide library member	MEDGRMKTLQEIGEM	AT5G28500.1	AT5G28500.2			
peptide library member	MEDIFNTSSRRTKSV	AT1G08640.1				
peptide library member	MEREARKSLETDLEE	AT1G50200.2				

peptide library member	MFGLRKSPANLPKHN	AT3G63490.2		
peptide library member	MGNESYEDAIEALKK	AT5G65780.2		
peptide library member	MGRMSRDSAAEASGG	AT2G34430.1		
peptide library member	MGSTPTDLPGEDVAD	AT4G02780.1		
peptide library member	MIHQPLGTAGGKATE	ATCG00480.1		
peptide library member	MIIRSPEPEVKILVD	AT2G40490.1		
peptide library member	MITSSLTSSLQALKL	AT5G13550.1		
peptide library member	MKIGSPSSPILSVVS	ATCG00020.1		
peptide library member	MLILDPETGEYSEEK	ATCG00270.1		
peptide library member	MLSLTATTLSSSIFT	AT5G05000.1	AT5G05000.2	AT5G05000.3
peptide library member	MNRKKGVTFGSFKVS	AT5G20250.1	AT5G20250.2	AT5G20250.3
peptide library member	MPGSEPSETQWPAKR	ATCG00560.1		
peptide library member	MPNPKAGTVTANIPQ	AT4G36970.1		
peptide library member	MQKRELEYCIENKRE	AT3G63160.1		
peptide library member	MRKASKPTGPSGSPW	AT3G47070.1		
peptide library member	MRLDRPGSVQASRLA	AT5G04360.1		
peptide library member	MRTNPTTSNPEVSIR	ATCG00130.1		
peptide library member	MSILQVSTSSLSSST	AT2G35410.1		

peptide library member	MSYASLSVKDLTSLV	AT4G13430.1	
peptide library member	MTAILERRESESLWG	AT5G40200.1	
peptide library member	MTIALGKFTKDEKDL	AT5G20250.4	
peptide library member	MTIDVLLYVDRLDVY	AT2G39730.1	
peptide library member	MTIKPAVRISDGNLI	AT5G51820.1	
peptide library member	MTQSNPNEQSVELNR	AT1G80300.1	
peptide library member	MVAGSPFSTGVLEAD	AT1G32060.1	
peptide library member	MVEKSGGEVNFPKLE	AT2G28800.1	AT2G28800.4
peptide library member	MVSSLLMSFAPATVR	AT5G61210.1	
peptide library member	NAKSAIGSLRKETDG	AT4G17740.2	
peptide library member	NANIGMFGTMKEITD	AT4G02510.1	
peptide library member	NASEDGDTVEDKNTS	ATCG01130.1	
peptide library member	NASFVADYRFDVSKL	AT1G06680.1	AT1G06680.2
peptide library member	NEASLPQSPEPVSAS	AT1G31330.1	
peptide library member	NEEEEDMTIKPAVRI	AT3G47470.1	
peptide library member	NFDPTARSDDGTCVY	AT2G03390.1	AT2G03390.2
peptide library member	NGGFIMSASHNPGGP	AT1G69830.1	
peptide library member	NGSLGESPSSSPEKS	AT1G73110.1	

peptide library member	NGTGLFQTIVGLKIR	AT2G05120.1	AT2G05120.2	
peptide library member	NKAVAKDTVELVEES	AT5G08540.1		
peptide library member	NKHTLNPSKRTTSEP	AT4G26670.1		
peptide library member	NKINPNFSIQISKAS	AT4G04770.1		
peptide library member	NKMSGQITVRTSSSD	AT2G47390.1		
peptide library member	NKSLEWTNSSLSEKK	AT5G19390.1	AT5G19390.2	AT5G19390.3
peptide library member	NLNVMVTPTDKKSIT	AT5G10000.1		
peptide library member	NPRSNKSLTQSVPKS	AT5G14200.1	AT5G14200.2	AT5G14200.3
peptide library member	NRDLSFTSIGSSAKT	AT2G20260.1		
peptide library member	NRPRHEVPFDSQDED	AT1G03130.1		
peptide library member	NRSFIPISLNLRSHF	AT5G42080.1	AT5G42080.3	
peptide library member	NRVPLIVTGNDFSTL	AT4G28750.1		
peptide library member	NRVPPPDSPVTPATQ	AT1G19870.1		
peptide library member	NSSVEEETEEEVEED	AT4G04020.1		
peptide library member	NTDGDEATDNDSSKA	AT5G51070.1		
peptide library member	NTKSRIISKGISAGH	AT3G60750.1	AT3G60750.2	
peptide library member	NTSDADTSPDQSKEG	AT2G37660.1		

peptide library member	NTTDSKISEESGKIS	ATCG00680.1							
member	NTTYFGLSSSRGNFG	AGI 1	AGI 2	AGI 3	AGI 4	AGI 5	AGI 6	AGI 7	AGI 8
peptide library member	NVLQKAGSLEGLEFD	AT3G60750.1	AT3G60750.2						
peptide library member	PAAIGGGSSKSTTIV	AT5G61210.1							
peptide library member	PAITTTTSAVKKLHL	AT1G42970.1							
peptide library member	PALRVEVTNAAIESL	AT3G25660.1							
peptide library member	PAPASSSSKDSPAAA	AT3G18890.1							
peptide library member	PASRTRETLESALLK	AT5G36880.2							
peptide library member	PDFKIRATDIDDEWG	AT3G54090.1							
peptide library member	PDSSTIRVTDKTSIA	AT1G23740.1							
peptide library member	PEGATLESDWSAKFA	AT1G14810.1							
peptide library member	PEGTGTPTKDFKALF	AT3G01500.1	AT3G01500.2	AT3G01500.3	AT5G14740.1	AT5G14740.2	AT5G14740.3	AT5G14740.4	AT5G14740.5
peptide library member	PEKLAFYDYIGNNPA	AT3G63160.1							
peptide library member	peptide	AT1G59870.1							
peptide library member	PESPGDATRNLSQQC	AT4G12800.1							
peptide library member	PFDSDDESDNKHTLN	AT5G42270.1							
peptide library member	PGAEAVGSGDPLEDF	AT1G59870.1							
peptide library member	PGGSSGGSAAAVAAR	AT1G42960.1							

peptide library member	PGPTDSDTDKSSTVA	AT3G25690.1	AT3G25690.2	AT3G25690.3	
peptide library member	PILSVVSSSGSLDPK	AT2G20260.1			
peptide library member	PKASINGSGITNGAA	AT3G09050.1			
peptide library member	PKGPFPFSRVADAFS	AT2G05120.1	AT2G05120.2		
peptide library member	PLAAEKGTIVVDNSS	AT3G53460.1	AT3G53460.2	AT3G53460.3	AT3G53460.4
peptide library member	PLDGNNSTDFIEDWV	AT1G48620.1			
peptide library member	PLFKKLSSSKDKSDS	AT5G17170.1			
peptide library member	PLGGGGVSMRRSISR	AT3G47860.1			
peptide library member	PMASQLRSSFSSASL	AT1G19870.1			
peptide library member	PMDFGRSKSKFQEVP	AT1G12900.1	AT1G12900.3	AT1G12900.4	
peptide library member	PMRRSLSTADGNRRG	AT2G34430.1			
peptide library member	PNGAALSTDEIEATA	AT4G14870.1			
peptide library member	PNLPRIRTQQQASSP	AT4G19490.1	AT4G19490.2		
peptide library member	PPATASSDSSSTTAA	AT3G01480.1	AT3G01480.2		
peptide library member	PPKPEGDTRRQELLA	AT1G59870.1			
peptide library member	PPPDSPVTPATQNRN	AT3G26840.1			

peptide library member	PPPKREESFSRGPRS	AT1G80300.1				
peptide library member	PPPTNVESGGEEVAV	AT5G14740.1	AT5G14740.2	AT5G14740.3	AT5G14740.4	AT5G14740.5
peptide library member	PRFGRKLTETQKARA	AT3G22960.1				
peptide library member	PSDGDSESMMMMMMR	AT1G67090.1				
peptide library member	PSFIAAQSKFEELTS	AT1G80300.1				
peptide library member	PSGDSALSVDGKIIK	ATCG00860.1	ATCG01280.1			
peptide library member	PSGSPWYGSDRVKYL	AT1G19870.1				
peptide library member	PSPAEESGSGEDKEV	ATCG00650.1				
peptide library member	PSPLTKSISDASSQS	AT3G09050.1				
peptide library member	PSTEKTRTVPLEIMV	AT4G09160.1				
peptide library member	PTYSRLRTTLMNAVV	AT4G09160.1				
peptide library member	PVSHFNIYKLLREKA	ATCG00650.1				
peptide library member	PVVSQDESGNGSLGE	AT2G39730.1	AT2G39730.2	AT2G39730.3		
peptide library member	QAASSSDSKSFDPVE	AT5G55220.1				
peptide library member	QAIGSSFSDKISEEI	AT3G08940.1	AT3G08940.2			
peptide library member	QCISFIAYKPPSFTG	AT4G30690.1	AT4G30690.2			
peptide library member	QDESGNGSLGESPSS	AT1G31330.1				

peptide library member	QDVSFVPSRRSENKE	AT5G64940.1	AT5G64940.2		
peptide library member	QEHNSPRSSPDLQER	AT1G68830.1			
peptide library member	QFERSESTPRTTSLR	AT4G15530.1	AT4G15530.4	AT4G15530.5	AT4G15530.6
peptide library member	QFLCLSKSGDGTSDS	AT2G28800.1	AT2G28800.4		
peptide library member	QIPRSLGSFKEETNK	ATCG00560.1			
peptide library member	QKIQIPRSLGSFKEE	AT5G40200.1			
peptide library member	QKQFERSESTPRTTS	AT1G66670.1			
peptide library member	QLAETYLSQAALGDA	ATCG00900.1	ATCG01240.1		
peptide library member	QLRQKYKSLGALKIV	AT5G01220.1			
peptide library member	QNLAKNLYGEVIGTR	AT1G03130.1			
peptide library member	QNMIGLVSKEEAVRR	AT3G03710.1			
peptide library member	QQIKKLESSLKLYAP	AT5G26742.2	AT5G26742.3		
peptide library member	QQKQVEVSVTPGGRW	AT4G01150.1	AT4G01150.2		
peptide library member	QRKLVKTVTETIDEI	AT5G65620.1			
peptide library member	QRLSIAKTGLHRETK	AT3G25690.1	AT3G25690.2	AT3G25690.3	
peptide library member	QSESEEGSDDEEEEA	AT4G24280.1			

peptide library member	QSNPNEQSVELNRTS	AT1G09420.1	AT1G09420.2	
peptide library member	QSPEPVSASEANPSP	AT3G22960.1		
peptide library member	QSPSRLPSFEELDTT	AT2G28800.1	AT2G28800.4	
peptide library member	QVPIEIGSTQGKALA	AT2G38040.1	AT2G38040.2	
peptide library member	QVREDDESEIDAPLL	AT5G26742.2	AT5G26742.3	
peptide library member	RAEKTESSSAAPAVK	AT3G26740.1		
peptide library member	RALLPESETDKDSQK	AT2G41040.1		
peptide library member	RAPSRERSFGGSCFI	AT1G17745.1	AT1G17745.2	
peptide library member	RASSEETSSIDTNEL	AT1G33810.1		
peptide library member	RAYLSRASSGDLDNT	AT2G39730.1	AT2G39730.2	AT2G39730.3
peptide library member	RDLSKNLSPKSQAKA	AT4G00690.1		
peptide library member	RDNKSLGSFRLDGIP	AT3G60750.1	AT3G60750.2	
peptide library member	RDVILGQYKSSSRDK	AT5G23060.1		
peptide library member	REEKRHESVPLQAIG	AT4G04770.1		
peptide library member	REGALASSTTSKPLP	AT5G08650.1		

peptide library member	RELAAEESDGSVKED	ATCG01000.1	ATCG01130.1
peptide library member	RERSFGGSCFICGKS	AT2G34430.1	
peptide library member	REYEEYNSPKTVFRE	AT1G48620.1	
peptide library member	RFPSAAISAVAPKSD	AT4G28730.1	
peptide library member	RFSTVGSDSDEYNPT	AT4G02510.1	
peptide library member	RGDDEDDSDKFSNAK	AT5G48960.1	
peptide library member	RGLAYDTSDDQQDIT	AT4G30950.1	
peptide library member	RGLSLQFSQVIRDVI	AT3G47470.1	
peptide library member	RHTPEGATLESDWSA	AT3G18680.1	
peptide library member	RIIPAASRSFGTRSG	AT2G47400.1	
peptide library member	RIISKGISAGHSRNC	AT2G41220.1	
peptide library member	RIPAPLDTAGKPLRA	AT1G59870.1	
peptide library member	RIPSPIFTKKLKGTS	AT5G63310.1	
peptide library member	RITMRKASKPTGPSG	AT2G11810.1	AT2G11810.2
peptide library member	RKDGTSPTVKPAASV	ATCG00900.1	ATCG01240.1
peptide library member	RKTVTENTVVIYSKT	AT2G46820.1	AT2G46820.2
peptide library member	RKVVEGDSAEEDENK	AT2G31170.1	

peptide library member	RLDDGFISADLGTLD	AT4G14870.1		
peptide library member	RLMKTIMTVCHVYDK	ATCG00280.1		
peptide library member	RLNRDLSFTSIGSSA	ATCG00750.1		
peptide library member	RLPSFDGTSKPPLKW	AT3G47860.1		
peptide library member	RMVVVKATSEGEISE	AT3G52230.1		
peptide library member	RNGKMPTVTMEQAQK	AT5G17520.1		
peptide library member	RNIEDIFSSGSRRTQ	AT4G32770.1		
peptide library member	RNIVHGSDSPENGKR	AT3G52230.1		
peptide library member	RNKVLASTLASHEWK	AT1G54520.1		
peptide library member	RNMAFKLSSELVDAA	AT5G16150.1	AT5G16150.2	AT5G16150.3
peptide library member	RNVVTRATTEVGEAP	AT3G04790.1		
peptide library member	RRCFTTLSSLTDGGA	AT1G02280.1	AT1G02280.2	
peptide library member	RRDTAGSESESEATP	AT4G31850.1		
peptide library member	RRFYHVETLFNGTLA	AT1G19870.1		
peptide library member	RRSGILLSFVRDVTP	AT1G48620.1		
peptide library member	RRSHSSVTVKCCCSS	AT2G39730.1	AT2G39730.2	AT2G39730.3
peptide library member	RSDKKEATGDDDQKD	AT4G02510.1		
peptide library member	RSINSESDSDSDFPH	AT4G15560.1		

peptide library member	RSISRVSASISTPNS	AT3G60750.1	AT3G60750.2	
peptide library member	RSNGSDSSSPPIKLP	AT2G41040.1		
peptide library member	RSRSSSSSSSQSYSV	AT4G32520.1	AT4G32520.2	
peptide library member	RSVRAQASSDGDEEE	AT4G19490.1	AT4G19490.2	
peptide library member	RTEEQARSLGIPLVG	AT2G39730.1	AT2G39730.2	AT2G39730.3
peptide library member	RTIDVLLYVDRLDVY	AT5G63420.1		
peptide library member	RTLFEKFTKDLGVQP	AT3G01500.1	AT3G01500.2	AT3G01500.3
peptide library member	RTRETLESALLKSPS	AT4G39040.1	AT4G39040.2	
peptide library member	RVDAGGASSVAPPPP	AT4G02510.1		
peptide library member	RWRGLAYDTSDDQQD	AT3G11670.1	AT3G11670.2	
peptide library member	SAKELDSSSEAVSGN	AT4G39040.1	AT4G39040.2	
peptide library member	SALSRLQSNPALREL	AT2G35410.1		
peptide library member	SAPAPLLYKEFGITV	AT4G02510.1		
peptide library member	SASTSVRSRFPSAAI	ATCG00650.1		
peptide library member	SCLTNKYSEGLPGKR	AT5G23060.1		

peptide library member	SDASSQSLSSILNNP	AT2G37220.1		
peptide library member	SDDQQDITRGKGMVD	AT5G23060.1		
peptide library member	SDSETKSSPKRVRKN	AT3G48730.1		
peptide library member	SDSRVCPSHVLDFQP	AT3G58610.1	AT3G58610.2	AT3G58610.3
peptide library member	SEASEEVSEIGDKEE	AT3G60210.1		
peptide library member	SEAVSGNSDKVGADD	AT3G48110.1		
peptide library member	SEFRRVYSAPEISRK	AT4G02510.1		
peptide library member	SERGIEDSEASEEVS	AT4G35770.1	AT4G35770.2	AT4G35770.3
peptide library member	SESEDGDSVEANNAS	AT1G80380.1	AT1G80380.2	AT1G80380.4
peptide library member	SESLKVGSVDAEEDS	AT1G29900.1		
peptide library member	SESTPRTTSLRTRKK	AT5G26570.1	AT5G26570.2	
peptide library member	SFAQVLSPSRIIPAA	AT2G47910.1	AT2G47910.2	
peptide library member	SFGSSGSGYGGGGGS	AT5G64280.1		
peptide library member	SFGTRSGTKFLPSSD	AT4G10340.1		
peptide library member	SFLVKAGSGVATLGL	AT5G23890.1		
peptide library member	SFMVDNCSTTARLGS	AT3G16290.1		
peptide library member	SFRINAVSTKWEPAK	AT3G51140.1		

peptide library member	SGDVIPKTDAGMVLA	AT5G61210.1				
peptide library member	SGEFEPVSDKAIEEV	AT2G34430.1				
peptide library member	SGMVKNPSFLRQVSS	AT5G23890.1				
peptide library member	SGSSLISSTLQHYNN	AT1G06690.1				
peptide library member	SGTLSEITKEDLYEV	AT5G14740.1	AT5G14740.2	AT5G14740.3	AT5G14740.4	AT5G14740.5
peptide library member	SHVNLILSTEGRSRT	AT5G19760.1				
peptide library member	SIGRLDLSPFQRAIG	AT5G36880.2				
peptide library member	SISSPRFTLRATASS	ATCG01130.1				
peptide library member	SKAVSETSDELAKWY	AT1G80300.1				
peptide library member	SKDELNIYSQDELDD	AT2G34590.1				
peptide library member	SKDLPVVSNRIPYTD	AT5G36880.1	AT5G36880.2			
peptide library member	SKLNNRNSAWPVLKS	AT2G35410.1				
peptide library member	SKPNPFDSDDESDNK	AT3G18890.1				
peptide library member	SKPTGPSGSPWYGSD	AT5G51020.1				
peptide library member	SKSPVPESTDGSKDE	AT4G37040.1				
peptide library member	SKVKLGGSDLKVTKL	AT5G13550.1				
peptide library member	SKVLAESESSAFEDQ	ATCG00120.1				
peptide library member	SLDCAMKTLKEGGPL	AT1G19870.1				

peptide library member	SLDPKISGSLGSRIL	AT3G20550.1		
peptide library member	SLEWTNSSLSEKKIK	AT3G25690.1	AT3G25690.2	AT3G25690.3
peptide library member	SLGESPSSSPEKSAP	AT1G19870.1		
peptide library member	SLKPFDLYTIGNSVK	AT5G62720.1	AT5G62720.2	
peptide library member	SLRHVESMSQLPSGA	AT3G23400.1		
peptide library member	SLRSASESEDGDSVE	AT1G19870.1		
peptide library member	SLSPGPTDSDTDKSS	AT5G36880.2		
peptide library member	SLTGEKFTREQASRD	AT5G42070.1		
peptide library member	SMQLSRTFSGLTDLL	AT4G12800.1		
peptide library member	SNNNSSSSNALLKEP	AT4G12800.1		
peptide library member	SPAPGIISRRSVYEP	AT5G26742.1		
peptide library member	SPDNNNVSEKQQQSF	AT5G26742.1		
peptide library member	SPERAIASRHDEGSN	AT5G23060.1		
peptide library member	SPGEGLNSVAASFHV	AT3G22960.1		
peptide library member	SPKQEIGTGEATEQE	AT1G79850.1		
peptide library member	SPMLANVSSRHSLGI	AT3G25690.1	AT3G25690.2	AT3G25690.3
peptide library member	SPPPSIRYSFSPLTT	ATCG00710.1		
peptide library member	SPRIQRSASQAQQGT	AT4G37040.1		

peptide library member	SPSSPILSVVSSSGS	AT4G32770.1		
peptide library member	SPSSVVETDNDDDND	AT2G32180.1		
peptide library member	SPTKRVSSFTVRAVK	AT3G63140.1		
peptide library member	SQLRSSFSSASLSQR	AT5G08540.1		
peptide library member	SRAPSRESFGGSCFI	AT2G20260.1		
peptide library member	SRESFGGSCFICGKS	AT3G18680.1		
peptide library member	SRLGTDSYNFSFAQV	AT4G12800.1		
peptide library member	SRPERTISVSYDGFA	AT4G02510.1		
peptide library member	SRPISKTKSFVALPV	AT3G53130.1		
peptide library member	SRSFYGGSPGRLSSS	AT1G59870.1		
peptide library member	SRSGPRSTTVGKLLK	AT3G12930.1		
peptide library member	SRTFSGLTDLLFNRR	AT5G66190.1		
peptide library member	SRVSASISTPNSETD	AT5G13550.1		
peptide library member	SRWSVWKTGKCKLPW	AT4G14070.1		
peptide library member	SSAGIYKSTEQPPHV	AT3G25690.1	AT3G25690.2	AT3G25690.3
peptide library member	SSDKEEDSNEEDDSN	AT3G63160.1		
peptide library member	SSDSSSTTAAAAPAK	AT3G53560.1		
peptide library member	SSFPRLPSFDGTSKP	AT5G26742.2	AT5G26742.3	

peptide library member	SSFSSASLSQRLAVP	AT5G26742.1			
peptide library member	SSGIEAHSDEANISN	AT1G54520.1			
peptide library member	SSGKNDESGIPIANA	AT1G54520.1			
peptide library member	SSGSRRTQSVNDDEE	AT2G40490.1			
peptide library member	SSLIPRKTSSSLSCL	AT4G24770.1			
peptide library member	SSLLTKISSVSPQRI	AT2G26540.1			
peptide library member	SSNALLKEPLLSVEK	AT5G14910.1			
peptide library member	SSPFLESSSFSGDAA	AT5G46110.1	AT5G46110.2	AT5G46110.3	AT5G46110.4
peptide library member	SSPSRSFYGGSPGRL	AT2G42975.1			
peptide library member	SSSKDKSDSDDATVP	AT1G50200.1			
peptide library member	SSSKFQNSITPLPKS	AT2G35410.1			
peptide library member	SSSSRAPSRERSFGG	AT1G80300.1			
peptide library member	SSSSRAPSRESFGGS	AT1G15500.1			
peptide library member	SSSSSQSYSVPRTSN	AT4G13430.1			
peptide library member	SSSSSSSQSYSVPRT	AT3G25690.1	AT3G25690.2	AT3G25690.3	
peptide library member	SSTLLSISPRKSLSS	AT1G48620.1			
peptide library member	SSVDEAETAVEKFNR	AT5G36880.2			
peptide library member	SSVLYPASLKAGNDI	AT1G67090.1	AT1G67090.2		

peptide library member	STHLLPLSKNLNFRT	AT4G14870.1					
peptide library member	STQTGIGTGIAIAGV	AT3G46780.1					
peptide library member	STSDGYLSDGGGSRG	AT3G08940.1	AT3G08940.2				
peptide library member	SVAVMPGSEPSETQW	AT2G20260.1					
peptide library member	SVEANNASEDGDTVE	AT2G35490.1					
peptide library member	SVKIPVVSQDESGNG	AT1G19870.1					
peptide library member	SVKIPVVSQEDAPSG	AT4G20360.1					
peptide library member	SVPFEPVYSDGNASF	AT4G02510.1					
peptide library member	SVQSSPSRSFYGGSP	AT4G28730.1					
peptide library member	SVSGGVETVKRRGRP	AT1G68830.1					
peptide library member	SVVSSSGSLDPKISG	AT5G53170.1					
peptide library member	SYLPDLTDSELAKEV	AT5G65780.2					
peptide library member	TAGSESESEATPSPA	AT1G08640.1					
peptide library member	TAIQNASETPKVQVA	ATCG00490.1					
peptide library member	TARFGFGTKKASPKK	AT1G19870.1					
peptide library member	TASSDSSSTTAAAAP	ATCG00270.1					
protein-control	Tau	AT1G12900.1	AT1G12900.2	AT1G12900.3	AT1G12900.4	AT1G42970.1	AT3G26650.1
peptide library member	TDEWGEKSGPELEES	AT3G52930.1					

peptide library member	TDIDSPDTTNTIKDH	AT1G74640.1					
peptide library member	TDVTGKVTKIMNDKD	AT4G19490.1	AT4G19490.2				
peptide library member	TEEMIFGSSEAAKQF	AT4G11960.1					
peptide library member	TENTVVIYSKTWCSY	ATCG00120.1					
peptide library member	TETIDEISDGRKTVW	AT3G53130.1					
peptide library member	TEVSELETNDRFVGG	AT1G12900.1	AT1G12900.2	AT1G12900.3	AT1G12900.4	AT1G42970.1	AT3G26650.1
peptide library member	TEVTNKVTTLDCSES	AT4G33580.1	AT4G33580.2				
peptide library member	TFGSFKVSKDIKYAD	AT1G06680.1	AT1G06680.2				
peptide library member	TGEIKGHYLNATAGT	AT5G08540.1					
peptide library member	THHEPVISTPDSKKR	AT3G46780.1					
peptide library member	TIALGKFTKDEKDLF	AT2G31170.1					
peptide library member	TIGKRLASINVENVE	AT2G21170.1	AT2G21170.2				
peptide library member	TKQKGSRYVPAAFLT	AT3G18890.1					
peptide library member	TKSISDASSQSLSSI	AT1G48860.1	AT1G48860.2	AT2G45300.1			
peptide library member	TLLPLKASTDQSGQV	AT5G42650.1					
peptide library member	TLTAEAESFLKEGIQ	AT1G67090.1					

peptide library member	TLTRTLSSGKNDESG	AT1G19870.1					
peptide library member	TMTTTHSYTGDQRLL	AT5G36700.1	AT5G36700.2	AT5G36700.4	AT5G36790.1	AT5G36790.2	AT5G36790.3
peptide library member	TNLQVMASGKTPGLT	AT4G33650.1	AT4G33650.2				
peptide library member	TNTDFLPYNGDGFKV	AT5G08280.1					
peptide library member	TPAAKKNSSVEEETE	AT4G25910.1					
peptide library member	TPKVQVATVRGQAKA	ATCG00480.1					
peptide library member	TQLRRCFTTLSSLTD	ATCG00480.1					
peptide library member	TRIIYGGSVNGGNSA	AT3G47070.1					
peptide library member	TRKSDSLSPGPTDSD	ATCG00710.1					
peptide library member	TRKTFPDYFQVLERI	AT1G48620.1					
peptide library member	TRPIKASGSETPDLT	ATCG00490.1					
peptide library member	TRQVQCISFIAYKPP	AT1G23740.1					
peptide library member	TSEVEMASKVEPEES	AT3G48870.1	AT3G48870.2	AT5G50920.1			
peptide library member	TSKISDFLSPKAATV	AT3G47070.1					
peptide library member	TSQVKSRSFLGRQAN	AT4G02510.1					
peptide library member	TTGDKILSQPLADIG	AT2G28800.1	AT2G28800.4				
peptide library member	TTMNLSLSTAEKNPN	AT2G32040.1					

peptide library member	TTSNPEVSIREKKNL	AT3G52150.1	AT3G52150.2	
peptide library member	TTSPIHKSAPAFIEL	AT2G28800.1	AT2G28800.4	
peptide library member	TTTGGRGTVRGGKNS	AT4G04020.1		
peptide library member	TVEDSSRSGPRSTTV	ATCG00280.1		
peptide library member	TVKPAASVSGGVETV	AT4G04020.1		
peptide library member	TYYTPEYETKDTDIL	AT1G06680.1	AT1G06680.2	
peptide library member	VALTGAVTPPGFRFV	AT3G27160.1		
peptide library member	VAVEIPFTPRAKRVL	AT4G02510.1		
peptide library member	VDEKEGTTTGGRGTV	AT2G46820.1	AT2G46820.2	
peptide library member	VEELPVSESLKVGSV	AT4G22890.1	AT4G22890.2	AT4G22890.3
peptide library member	VEESQSESEEGSDDE	AT4G11960.1		
peptide library member	VEGDRDTTSSIRTQP	AT1G17745.1	AT1G17745.2	
peptide library member	VEKVQVMYDKYSGRS	ATCG00490.1		
peptide library member	VELVEESQSESEEGS	AT4G18480.1		
peptide library member	VERVFASSSTVSVAD	AT1G42970.1		
peptide library member	VETLFNGTLALAGRD	AT1G79050.1		

peptide library member	VFASSSTVSVADKAI	AT1G29930.1					
peptide library member	VFGKPKTNTDFLPYN	AT1G22700.1	AT1G22700.2	AT1G22700.3			
peptide library member	VFVEDNESEERLVNR	AT4G15530.1	AT4G15530.2	AT4G15530.3	AT4G15530.4	AT4G15530.5	AT4G15530.6
peptide library member	VGADDLSDSEKEKPN	AT1G36390.1	AT1G36390.2				
peptide library member	VGEAPATTTEAETTE	AT5G14740.1	AT5G14740.2	AT5G14740.3	AT5G14740.4	AT5G14740.5	
peptide library member	VGGDNVDSNVLPYCS	AT1G76080.1					
peptide library member	VGGEEVDSKILPYCS	AT5G48300.1					
peptide library member	VGSDSDEYNPTLPKP	AT3G25480.1					
peptide library member	VKEYKLTYYTPEYET	AT5G11670.1					
peptide library member	VKISRVCSELNVDGL	AT1G29910.1	AT1G29920.1				
peptide library member	VKIVDNETISVDGKL	AT5G23890.1					
peptide library member	VKKGSWYSYEDQRLG	AT4G03520.1	AT4G03520.2				
peptide library member	VKLSPAASEVLGSGR	AT3G56690.1					
peptide library member	VKLYKGVVAVKSKKR	ATCG00480.1					
peptide library member	VKNDTDLSAADLKEL	AT2G40100.1	AT2G40100.2				

peptide library member	VKTLIRSYKQALLNG	AT3G52150.1	AT3G52150.2				
peptide library member	VLAESESSAFEDQCG	AT2G48120.1	AT2G48120.2				
peptide library member	VLKLSRSMSETVVFA	ATCG01110.1					
peptide library member	VLTQFNSASLNRHLS	AT1G74070.1					
peptide library member	VMFYLRKYKPISAMN	AT3G01500.1	AT3G01500.2	AT3G01500.3	AT5G14740.1	AT5G14740.2	AT5G14740.4
peptide library member	VMHNLRQYTVPLQRY	AT3G27925.1					
peptide library member	VNLSPAASEVLGSGR	AT1G15500.1					
peptide library member	VPESTDGSKDELNIY	AT5G66190.1					
peptide library member	VPKTTLTSSLDKFLP	AT5G21930.1	AT5G21930.2	AT5G21930.3			
peptide library member	VPSSTQRSDILHIIL	AT5G42080.1	AT5G42080.3				
peptide library member	VQAGSEVSALLGRMP	AT1G19870.1					
peptide library member	VQARFGFSFGKKKPA	AT1G06680.1	AT1G06680.2				
peptide library member	VQVMYDKYSGRSRRF	AT4G02510.1					
peptide library member	VRIEEDATTGSKGEG	AT5G65780.2					
peptide library member	VRLSEMTESIKIIQQ	AT5G24490.1					
peptide library member	VSGKAGITYRRKDFV	AT5G16715.1					

peptide library member	VSLANLLTYPFVREG	AT5G35170.1	AT5G35170.2	
peptide library member	VSNGSDLYRILDQCK	AT2G28000.1		
peptide library member	VSQEDAPSGETTSQL	AT2G20260.1		
peptide library member	VSVKAQVTTDTTEAP	AT3G12780.1		
peptide library member	VTADTAESLAKRLTE	AT1G59870.1		
peptide library member	VTNAAIESLDKMREG	AT1G67090.1		
peptide library member	VTNGKQVSPRIQRSA	AT4G13430.1		
peptide library member	VTPTDKKSITDYGSP	AT1G15820.1		
peptide library member	VTRVDGSESEEETEE	AT4G08510.1		
peptide library member	VTTLDCSESPSEAGR	AT2G35500.1		
peptide library member	VVEDVEDSTDSSVGE	AT3G60750.1	AT3G60750.2	
peptide library member	VVEKMLASEGIKRVD	AT3G25690.1	AT3G25690.2	AT3G25690.3
peptide library member	VVFEETQTLLSQIQL	AT5G26742.1	AT5G26742.2	AT5G26742.3
peptide library member	VVLDEFGSPKVVNDG	AT4G30950.1		
peptide library member	VVRAAEDTPPATASS	AT1G79460.1		
peptide library member	VVSMAKKSVGDLTSA	AT3G04790.1		
peptide library member	VYGNQLMSKEVDVTK	AT4G21280.1	AT4G21280.2	

peptide library member	VYREHGNSPGYYDGR	AT3G08940.1	AT3G08940.2	
peptide library member	VYSDGNASFVADYRF	ATCG00490.1		
peptide library member	WATPWSKTAENFANY	AT4G21280.1	AT4G21280.2	
peptide library member	WCKKFKPSLLQKLPI	AT2G42975.1		
peptide library member	WDPTHAKSVAQASLS	AT1G09130.1	AT1G09130.2	AT1G09130.3
peptide library member	WEKALPTYTPESPGD	AT4G01690.1	AT4G01690.2	
peptide library member	WGKSKDDSSVQSSPS	AT5G16010.1		
peptide library member	WLIGGRSSSSSRAPS	AT1G74640.1		
peptide library member	WNWRLMKTIMTVCHV	AT4G33760.1		
peptide library member	YARDLNLTIPLGSEV	ATCG01000.1	ATCG01130.1	
member	YDIVGIPTSKRTEEQ			
member	YDLNTIISSKPKDEK			
member	YGEVIGTRTEAVDPK			
member	YLNATAGTCEEMIKR			
member	YLRYDLNTIISSKPK			
member	YLSDGGGSRGLTIRD			
member	YNEHRPRTPPPDLPS			
member	YPKEAIRTECLIDGE			
member	YSKFGVSSSSPQPQK			
member	YVPAAFLTGLLDPVS			
member	YVVLVEGTVRSRPNE			
member	YYLGRIPSPIFTKKL			

Supplemental Table S3: List of peptides phosphorylated by recombinant pCKII and protein kinase A. The phosphorylation category was determined by calculating the grayscale intensity median of all spot pixels after background subtraction. These intensity values were averaged over all 9 peptide spots and classified into 4 categories. The categorries are defined as 3 - strong(>50% segregation of background intensity [BI]); 2- medium(>20% - <50% BI); 1 - weak(>10% - <20% BI); 0 – none(<10% BI).

Peptide	Identifier	Annotation	Phosphorylation category	
			rec. pCKII	PKA
AFKSLGLSDHDEYDL	AT5G26742.1	emb1138 | DEAD box RNA helicase (RH3)	3	0
	AT5G26742.2	emb1138 | DEAD box RNA helicase (RH3)		
DASEGDVSEGDESEG	AT4G24770.1	RBP31, ATRBP31, CP31, ATRBP33	2	0
DEEEGNVSDRGDEDE	AT4G12610.1	RAP74, ATRAP74	1	0
	AT4G12610.2	RAP74, ATRAP74		

DNEVEPVTDDDNDRA	AT5G19390.4	Rho GTPase activation protein (RhoGAP) with PH domain	1	0
	AT5G19390.3	Rho GTPase activation protein (RhoGAP) with PH domain		
	AT5G19390.1	Rho GTPase activation protein (RhoGAP) with PH domain		
	AT5G19390.2	Rho GTPase activation protein (RhoGAP) with PH domain		
DNGGDVIDADFTDSN	AT5G49910.1	CPHSC70-2EAT SHOCK PROTEIN 70-2, HSC70-7, cpHsc70-2	1	0
DVSEGDESEGDVSEG	AT4G24770.1	RBP31, ATRBP31, CP31, ATRBP33	1	0
ELPRRRDSEESLLLD	AT2G32040.1	Major facilitator superfamily protein	0	1
ELYQRALSIDANTES	AT3G17040.2	HCF107	0	2
	AT3G17040.1	HCF107		
EQKSGRRTSFGYDQE	AT1G19870.1	iqd32	0	2
FDHCFKKSSDGFLYC	AT5G11880.1	Pyridoxal-dependent decarboxylase family protein	0	1
FEGKSGRSFDIDFDE	AT1G78915.1	Tetratricopeptide repeat (TPR)-like superfamily protein	0	1
	AT1G78915.2	Tetratricopeptide repeat (TPR)-like superfamily protein		
	AT1G78915.3	Tetratricopeptide repeat (TPR)-like superfamily protein		
FFEGGFGSDDDPTSP	AT1G64510.1	Translation elongation factor EF1B	3	0
FVGGEETKSGGEEAE	AT5G53170.1	FTSH11	1	0
GDGTSDSDSDPDPPK	AT3G09050.1	unknown protein	2	0
GFNRLDDSAEFFTSD	AT2G36390.1	SBE2.1, BE3	1	0
GFSRGPRSSFGSSGS	AT2G37220.1	RNA-binding (RRM/RBD/RNP motifs) family protein	0	1

GGLFRAGSMDNGDGI	ATCG00680.1	PSBB	0	1
GGNSFSSRSRSSSSS	AT1G54520.1	unknown protein	0	1
GKKLSELSDDEDFDE	AT3G48500.1	PDE312, PTAC10	2	0
	AT3G48500.2	PDE312, PTAC10		
GKRSSVLYPASLKAG	AT2G26540.1	HEMD, UROS, ATUROS, ATDUF3, DUF3	0	2
GLSDHDEYDLDGDNN	AT5G26742.1	emb1138 | DEAD box RNA helicase (RH3)	2	0
	AT5G26742.2	emb1138 | DEAD box RNA helicase (RH3)		
GRRTSFGYDQEARES	AT1G19870.1	iqd32	0	2
GSAQRKPSFFEPISK	ATCG01130.1	YCF1.2	0	3
GSDKGILSDVELLKE	AT5G13630.1	GUN5, CCH, CHLH, CCH1, ABAR	1	0
	AT5G13630.2	GUN5, CCH, CHLH, CCH1, ABAR		
GSESEEETEEMIFGS	AT4G02510.1	TOC159, TOC86, PPI2, TOC160, ATTOC159	3	0
GVLYRSFSGDADLRY	AT4G34240.1	ALDH3I1, ALDH3	0	1
HLLPAFSISRCFSSV	AT1G32470.1	Single hybrid motif superfamily protein	0	1
HSLKRKVSNPSFIAA	AT1G19870.1	iqd32	0	2
IDGQIVTDSDEDVDT	AT4G02510.1	TOC159, TOC86, PPI2, TOC160, ATTOC159	1	0
KAEKDEVSDDEATIE	AT1G58200.1	MSL3	1	0
	AT1G58200.2	MSL3		

KDDDEDDQSSDGHED	AT3G52230.1	unknown protein	1	0
KGFVADDSDIESPRD	AT5G19390.2	Rho GTPase activation protein (RhoGAP) with PH domain	1	0
	AT5G19390.1	Rho GTPase activation protein (RhoGAP) with PH domain		
	AT5G19390.4	Rho GTPase activation protein (RhoGAP) with PH domain		
	AT5G19390.3	Rho GTPase activation protein (RhoGAP) with PH domain		
KQLRSSSYDLDVKKP	AT5G17230.1	PSY	0	1
	AT5G17230.2	PSY		
	AT5G17230.3	PSY		
KYEGKKLSELSDDED	AT3G48500.1	PDE312, PTAC10	2	0
	AT3G48500.2	PDE312, PTAC10		
LLLHRKMSSNSLRHV	AT5G36880.2	ACS	0	2
LSASSSLSFVRSLVS	AT4G08390.4	SAPX	0	1
	AT4G08390.2	SAPX		
	AT4G08390.3	SAPX		
	AT4G08390.1	SAPX		
LSEEAFKSLGLSDHD	AT5G26742.1	emb1138 | DEAD box RNA helicase (RH3)	2	0
	AT5G26742.2	emb1138 | DEAD box RNA helicase (RH3)		

LSKIVKKSVADILGQ	AT1G52220.3	molecular_function unknown	0	1
	AT1G52220.1	molecular_function unknown		
	AT1G52220.2	molecular_function unknown		
LSKSGDGTSDSDSDP	AT3G09050.1	unknown protein	2	0
LSYLPDLSDVELAKE	AT5G38420.1	Ribulose bisphosphate carboxylase (small chain) family protein	2	0
	AT5G38410.2	Ribulose bisphosphate carboxylase (small chain) family protein		
	AT5G38410.1	Ribulose bisphosphate carboxylase (small chain) family protein		
	AT5G38410.3	Ribulose bisphosphate carboxylase (small chain) family protein		
LVRNSSKTSLLPFRN	AT5G23040.1	CDF1	0	2
MEASLAALERPRGSA	AT5G19390.1	Rho GTPase activation protein (RhoGAP) with PH domain	0	1
	AT5G19390.2	Rho GTPase activation protein (RhoGAP) with PH domain		
	AT5G19390.3	Rho GTPase activation protein (RhoGAP) with PH domain		

	AT5G19390.4	Rho GTPase activation protein (RhoGAP) with PH domain		
MEDIFNTSSRRTKSV	AT1G15210.1	PDR7, ATPDR7	0	2
MRKASKPTGPSGSPW	AT2G34430.1	LHB1B1, LHCB1.4	0	2
MTAILERRESESLWG	ATCG00020.1	PSBA	0	3
NSSVEEETEEEVEED	AT5G08540.1	unknown protein	1	0
PDFKIRATDIDDEWG	AT4G04020.1	FIB	1	0
PFDSDDESDNKHTLN	AT5G61210.1	SNAP33, ATSNAP33, SNP33, ATSNAP33B	3	0
PLGGGGVSMRRSISR	AT1G59870.1	PEN3, PDR8, ATPDR8, ABCG36, ATABCG36	0	2
PMASQLRSSFSSASL	AT4G12800.1	PSAL	0	1
QRLSIAKTGLHRETK	AT4G15530.1	PPDK	0	1
	AT4G15530.4	PPDK		
	AT4G15530.5	PPDK		
	AT4G15530.6	PPDK		
QSESEEGSDDEEEEA	AT2G28800.4	ALB3	2	0
	AT2G28800.1	ALB3		
RAPSRERSFGGSCFI	AT5G26742.3	emb1138 | DEAD box RNA helicase (RH3)	0	1
	AT5G26742.2	emb1138 | DEAD box RNA helicase (RH3)		

RAYLSRASSGDLDNT	AT5G65620.1	Zincin-like metalloproteases family protein	0	1
RGLAYDTSDDQQDIT	AT2G39730.1	RCA	1	0
	AT2G39730.2	RCA		
	AT2G39730.3	RCA		
RITMRKASKPTGPSG	AT2G34430.1	LHB1B1, LHCB1.4	0	2
RLPSFDGTSKPPLKW	AT3G18680.1	Amino acid kinase family protein	0	1
RWRGLAYDTSDDQQD	AT2G39730.1	RCA	1	0
	AT2G39730.2	RCA		
	AT2G39730.3	RCA		
SASTSVRSRFPSAAI	AT2G41040.1	S-adenosyl-L-methionine-dependent methyltransferases superfamily protein	0	2
SKPNPFDSDDESDNK	AT5G61210.1	SNAP33, ATSNAP33, SNP33, ATSNAP33B	2	0
SMQLSRTFSGLTDLL	AT4G37040.1	MAP1D	0	1
SPAPGIISRRSVYEP	ATCG00120.1	ATPA	0	2
SPPPSIRYSFSPLTT	AT3G23400.1	FIB4	0	1
SPRIQRSASQAQQGT	AT1G19870.1	iqd32	0	1
SQLRSSFSSASLSQR	AT4G12800.1	PSAL	0	2
SRAPSRESFGGSCFI	AT5G26742.1	emb1138 | DEAD box RNA helicase (RH3)	0	2
SRESFGGSCFICGKS	AT5G26742.1	emb1138 | DEAD box RNA helicase (RH3)	0	1
SRPERTISVSYDGFA	AT3G22960.1	PKP1, PKP-ALPHA	0	2
SRSFYGGSPGRLSSS	AT3G25690.3	CHUP1	0	1

	AT3G25690.1	CHUP1		
	AT3G25690.2	CHUP1		
SSLIPRKTSSSLSCL	AT3G12930.1	Lojap-related protein	0	2
SSSSRAPSRESFGGS	AT5G26742.1	emb1138 | DEAD box RNA helicase (RH3)	0	1
SSTLLSISPRKSLSS	AT2G40490.1	HEME2	0	3
TDEWGEKSGPELEES	AT2G35490.1	Plastid-lipid associated protein PAP / fibrillin family protein	1	0
VEESQSESEEGSDDE	AT2G28800.4	ALB3	2	0
	AT2G28800.1	ALB3		
VLAESESSAFEDQCG	AT5G14740.5	CA2, CA18, BETA CA2	1	0
	AT5G14740.3	CA2, CA18, BETA CA2		
	AT5G14740.4	CA2, CA18, BETA CA2		
	AT5G14740.1	CA2, CA18, BETA CA2		
	AT5G14740.2	CA2, CA18, BETA CA2		
VTRVDGSESEEETEE	AT4G02510.1	TOC159, TOC86, PPI2, TOC160, ATTOC159	1	0

Supplemental Table S4: Proteins identified and quantified from the Heparin-Sepharose eluate fraction with kinase activity. Quantification was performed by HD-MSE analysis as described (Helm et al., 2014, Materials and Methods). Listed are those proteins that were detected in at least two of the three replicates. Original MS data were uploaded to the PRIDE repository (http://proteomecentral.proteomexchange.org) and are accesible with the dataset identifier PXD000981.

Identifier	Annotation	technical triplicate [fmol/column]			
		1	2	3	mean
AT1G09340.1	chloroplast RNA binding	67,0643	58,6048	62,1207	62,6
AT3G25920.1	ribosomal protein L15	52,0182	49,8102	52,0076	51,3
ATCG00160.1	ribosomal protein S2	50,7589	45,2506	40,878	45,6
ATCG00790.1	ribosomal protein L16	47,1167	44,2599	44,103	45,2
AT2G33800.1	Ribosomal protein S5 family protein	48,223	38,4229	41,996	42,9
AT3G15190.1	chloroplast 30S ribosomal protein S20, putative	42,8937	37,0553	39,8155	39,9
AT3G54210.1	Ribosomal protein L17 family protein	40,1117	38,0366	38,1034	38,8
AT3G63140.1	chloroplast stem-loop binding protein of 41 kDa	39,4065	36,6091	37,7144	37,9
ATCG01240.1	ribosomal protein S7	38,7966	33,488	37,9592	36,7
ATCG00830.1	ribosomal protein L2	37,5492	34,3544	36,2244	36,0
AT3G44890.1	ribosomal protein L9	36,9303	33,6057	36,2443	35,6
ATCG01120.1	chloroplast ribosomal protein S15	34,1339	35,5659	36,0515	35,3
ATCG00380.1	chloroplast ribosomal protein S4	34,1387	31,9533	33,524	33,2
AT1G05190.1	Ribosomal protein L6 family	33,2984	31,8677	33,1435	32,8
AT2G33450.1	Ribosomal L28 family	36,5465	28,3252	29,2869	31,4
AT2G24090.1	Ribosomal protein L35	31,5374	30,585	31,8283	31,3
ATCG00650.1	ribosomal protein S18	34,391	27,9295	30,1846	30,8
AT1G79850.1	ribosomal protein S17	34,1811	30,4636	25,4032	30,0
ATCG00770.1	ribosomal protein S8	29,6627	27,1218	29,6603	28,8
ATCG00065.1	ribosomal protein S12A	27,9571	29,1938	28,6182	28,6
AT5G23060.1	calcium sensing receptor	28,9697	27,3641	28,9277	28,4
AT3G27850.1	ribosomal protein L12-C	30,8899	28,71	25,3577	28,3
ATCG00750.1	ribosomal protein S11	33,8836	23,1464	25,3038	27,4
AT1G74970.1	ribosomal protein S9	29,4856	25,2782	26,4269	27,1
AT5G25980.2	glucoside glucohydrolase 2	27,2396	24,347	27,2885	26,3
AT5G54600.1	Translation protein SH3-like family protein	26,0524	22,855	22,8193	23,9
AT5G40950.1	ribosomal protein large subunit 27	26,9599	25,9515	18,7261	23,9
AT2G01250.1	Ribosomal protein L30/L7 family protein	24,2274	22,0516	22,336	22,9
AT4G37930.1	serine transhydroxymethyltransferase 1	25,297	19,8095	23,3398	22,8
AT5G14320.1	Ribosomal protein S13/S18 family	24,2108	20,3769	22,101	22,2

AT3G49910.1	Translation protein SH3-like family protein	21,5834	19,295	19,4383	20,1
AT1G29910.1	chlorophyll A/B binding protein 3	24,744	13,6448	19,8455	19,4
AT5G47190.1	Ribosomal protein L19 family protein	19,256	16,9716	21,1453	19,1
AT2G34430.1	light-harvesting chlorophyll-protein complex II subunit B1	24,744	13,8412	18,4323	19,0
AT2G38040.1	acetyl Co-enzyme a carboxylase carboxyltransferase alpha subunit	20,0346	17,5479	17,5432	18,4
AT1G07320.1	ribosomal protein L4	18,2954	17,5698	18,1683	18,0
AT1G65260.1	plastid transcriptionally active 4	18,3925	16,9772	17,4145	17,6
AT1G07770.1	ribosomal protein S15A	18,9401	16,5229	16,7985	17,4
AT3G05560.1	Ribosomal L22e protein family	19,8644	14,7867	17,3414	17,3
ATCG00490.1	ribulose-bisphosphate carboxylases	17,1363	16,2799	16,8985	16,8
ATCG00760.1	ribosomal protein L36	20,1389	14,4654	15,3992	16,7
AT5G15200.1	Ribosomal protein S4	19,0366	13,6534	16,2154	16,3
AT1G22780.1	Ribosomal protein S13/S18 family	18,5151	14,7465	15,0812	16,1
ATCG00800.1	structural constituent of ribosome	18,0304	13,123	17,1137	16,1
AT3G63490.1	Ribosomal protein L1p/L10e family	17,1282	15,2025	15,4526	15,9
AT5G27770.1	Ribosomal L22e protein family	17,6994	13,645	16,318	15,9
AT1G18540.1	Ribosomal protein L6 family protein	16,2928	14,6988	16,0781	15,7
AT4G17390.1	Ribosomal protein L23/L15e family protein	19,0935	12,9142	12,8433	15,0
ATCG00820.1	ribosomal protein S19	16,198	13,4537	14,0508	14,6
AT3G06730.1	Thioredoxin z	14,006	14,4829	14,1256	14,2
AT1G66580.1	senescence associated gene 24	16,2018	12,4614	13,9271	14,2
ATCG00330.1	chloroplast ribosomal protein S14	14,6343	16,8576	10,9064	14,1
AT5G27850.1	Ribosomal protein L18e/L15 superfamily protein	14,2647	13,3626	14,6642	14,1
AT4G10340.1	light harvesting complex of photosystem II 5	13,7076	13,9828	14,5412	14,1
ATCG00500.1	acetyl-CoA carboxylase carboxyl transferase subunit beta	13,6325	14,2319	14,3137	14,1
AT3G46040.1	ribosomal protein S15A D	15,0974	13,4306	13,107	13,9
ATCG00180.1	DNA-directed RNA polymerase family protein	13,2989	15,977	12,0531	13,8
AT4G20360.1	RAB GTPase homolog E1B	13,3325	13,3583	14,4814	13,7

AT2G43030.1	Ribosomal protein L3 family protein	14,4307	13,1198	13,5215	13,7
AT2G17360.1	Ribosomal protein S4 (RPS4A) family protein	15,2094	12,3789	13,0438	13,5
AT1G73990.1	signal peptide peptidase	13,373	13,5729	13,2086	13,4
ATCG00120.1	ATP synthase subunit alpha	13,7071	12,8889	13,5388	13,4
AT1G31330.1	photosystem I subunit F	14,1049	13,122	12,6461	13,3
AT5G02940.1	Protein of unknown function (DUF1012)	13,7283	12,7766	13,3072	13,3
AT3G55280.3	ribosomal protein L23AB	14,55	11,9247	12,8867	13,1
AT4G04640.1	ATPase, F1 complex, gamma subunit protein	12,4078	12,8808	13,4355	12,9
AT4G31700.1	ribosomal protein S6	13,2873	13,006	12,349	12,9
AT5G65220.1	Ribosomal L29 family protein	12,4634	10,9496	15,0513	12,8
AT3G08580.1	ADP/ATP carrier 1	14,1374	12,1863	12,1109	12,8
AT3G24830.1	Ribosomal protein L13 family protein	14,1972	11,6687	12,3357	12,7
AT2G22230.1	Thioesterase superfamily protein	12,8728	12,7506	12,324	12,6
ATCG00740.1	RNA polymerase subunit alpha	13,5152	11,9245	11,915	12,5
AT1G27400.1	Ribosomal protein L22p/L17e family protein	13,063	11,8043	11,7711	12,2
ATCG00810.1	ribosomal protein L22	12,6253	11,6059	12,2208	12,2
AT3G09500.1	Ribosomal L29 family protein	11,2589	12,302	12,755	12,1
AT5G35970.1	P-loop containing nucleoside triphosphate hydrolases superfamily protein	13,1752	11,2739	11,662	12,0
AT3G04920.1	Ribosomal protein S24e family protein	11,6736	11,8589	11,8203	11,8
AT4G39200.2	Ribosomal protein S25 family protein	13,0487	10,9809	11,313	11,8
AT3G07110.1	Ribosomal protein L13 family protein	13,2347	11,1533	10,8843	11,8
AT4G01310.1	Ribosomal L5P family protein	12,9449	10,8838	10,9899	11,6
ATCG00480.1	ATP synthase subunit beta	12,3663	11,0971	10,9975	11,5
AT4G17560.1	Ribosomal protein L19 family protein	12,1258	11,5537	10,6168	11,4
AT5G20290.1	Ribosomal protein S8e family protein	13,0545	10,6697	10,4803	11,4
AT5G26742.1	DEAD box RNA helicase (RH3)	8,7818	16,4235	8,8837	11,4
ATCG00170.1	DNA-directed RNA polymerase family protein	11,4534	11,2778	11,2158	11,3
AT3G04840.1	Ribosomal protein S3Ae	10,0635	14,5945	9,2875	11,3
AT2G34640.1	plastid transcriptionally active 12	18,6143	7,4931	7,8049	11,3

AT5G10160.1	Thioesterase superfamily protein	12,4983	10,7674	10,5573	11,3
AT5G01590.1	unknown protein; FUNCTIONS IN: molecular_function unknown; INVOLVED IN: biological_process unknown; LOCATED IN: chloroplast, chloroplast envelope; EXPRESSED IN: 22 plant structures; EXPRESSED DURING: 13 growth stages; Has 60 Blast hits to 59 proteins in 31 species: Archae - 0; Bacteria - 20; Metazoa - 1; Fungi - 2; Plants - 33; Viruses - 0; Other Eukaryotes - 4 (source: NCBI BLink).	12,8794	11,5265	9,1201	11,2
ATCG00190.1	RNA polymerase subunit beta	9,9744	14,4139	8,9289	11,1
AT3G04260.1	plastid transcriptionally active 3	10,598	11,4017	11,0924	11,0
AT3G62870.1	Ribosomal protein L7Ae/L30e/S12e/Gadd45 family protein	9,6978	9,8048	13,2873	10,9
AT1G74730.1	Protein of unknown function (DUF1118)	10,3223	11,004	11,0271	10,8
AT3G49010.4	breast basic conserved 1	12,2525	9,9242	10,1725	10,8
AT3G56910.1	plastid-specific 50S ribosomal protein 5	10,7409	11,1161	10,4113	10,8
AT1G33120.1	Ribosomal protein L6 family	11,7427	9,379	11,0724	10,7
AT1G70600.1	Ribosomal protein L18e/L15 superfamily protein	11,2974	10,7808	10,0698	10,7
AT5G18380.2	Ribosomal protein S5 domain 2-like superfamily protein	10,2057	9,6852	12,0924	10,7
AT1G74050.1	Ribosomal protein L6 family protein	10,1928	10,0584	11,2335	10,5
ATCG01130.1	Ycf1 protein	10,5441	10,069	10,6888	10,4
AT5G64940.1	ABC2 homolog 13	10,9287	9,4891	10,7841	10,4
AT3G09630.2	Ribosomal protein L4/L1 family	6,7599	11,988	12,3371	10,4
AT5G22880.1	histone B2	11,6068	9,7975	9,6248	10,3
AT5G38420.1	Ribulose bisphosphate carboxylase (small chain) family protein	4,8127	10,234	15,8424	10,3
AT1G01080.1	RNA-binding (RRM/RBD/RNP motifs) family protein	11,2289	9,4622	9,9862	10,2

AT1G14320.1	Ribosomal protein L16p/L10e family protein	16,2018	13,4525	0,985	10,2
P00489	-	10	10	10	10,0
AT1G67090.1	ribulose bisphosphate carboxylase small chain 1A	8,7238	8,4558	12,3915	9,9
AT2G44120.1	Ribosomal protein L30/L7 family protein	14,1614	0,8436	14,4628	9,8
AT3G48500.1	Nucleic acid-binding, OB-fold-like protein	10,1341	8,9843	10,0292	9,7
AT4G27090.1	Ribosomal protein L14	10,3597	8,7821	9,601	9,6
AT5G10360.1	Ribosomal protein S6e	10,3783	8,3143	9,999	9,6
AT2G34480.1	Ribosomal protein L18ae/LX family protein	10,7038	9,0167	8,762	9,5
ATCG00780.1	ribosomal protein L14	8,501	9,8117	9,9916	9,4
AT5G02450.1	Ribosomal protein L36e family protein	10,0521	9,1343	9,0483	9,4
AT5G28060.1	Ribosomal protein S24e family protein	9,3335	9,7804	8,5655	9,2
AT5G26000.2	thioglucoside glucohydrolase 1	9,125	8,9734	8,7506	8,9
AT2G41840.1	Ribosomal protein S5 family protein	8,564	8,2727	9,8959	8,9
AT1G26880.1	Ribosomal protein L34e superfamily protein	9,447	8,3398	8,8871	8,9
AT1G74850.1	plastid transcriptionally active 2	9,0012	8,8472	8,5597	8,8
AT5G51100.1	Fe superoxide dismutase 2	9,7702	8,5464	8,0564	8,8
AT1G07660.1	Histone superfamily protein	9,4103	8,1964	8,4147	8,7
AT1G43170.1	ribosomal protein 1	10,2512	7,5924	8,1039	8,6
AT2G18020.1	Ribosomal protein L2 family	9,7094	7,5677	8,5392	8,6
AT5G02870.2	Ribosomal protein L4/L1 family	14,4228	5,7896	5,5414	8,6
AT3G10690.1	DNA GYRASE A	8,4786	8,3842	8,1137	8,3
AT3G09200.1	Ribosomal protein L10 family protein	12,2178	0	12,5334	8,3
AT5G45775.1	Ribosomal L5P family protein	0	12,852	11,5925	8,1
AT3G05590.1	ribosomal protein L18	8,5303	8,3391	7,2577	8,0
AT3G02080.1	Ribosomal protein S19e family protein	10,1288	7,3697	6,4378	8,0
AT3G11250.1	Ribosomal protein L10 family protein	0	11,3707	12,4476	7,9
AT5G16130.1	Ribosomal protein S7e family protein	8,4325	7,1518	7,7059	7,8
AT1G42970.1	glyceraldehyde-3-phosphate dehydrogenase B subunit	7,7476	7,6354	7,8918	7,8
AT3G53430.1	Ribosomal protein L11 family protein	7,698	7,5595	7,892	7,7
AT3G54090.1	fructokinase-like 1	7,5994	7,1203	7,7692	7,5

AT1G63680.1	acid-amino acid ligases;ligases;ATP binding;ATP binding;ligases	8,2009	6,637	7,6153	7,5
AT3G13120.1	Ribosomal protein S10p/S20e family protein	9,6832	5,8276	6,8958	7,5
ATCG00840.1	ribosomal protein L23.1	7,4457	7,6009	7,1923	7,4
AT3G57150.1	homologue of NAP57	10,8933	10,1279	1,1591	7,4
AT3G03960.1	TCP-1/cpn60 chaperonin family protein	7,3986	6,1489	8,2814	7,3
AT4G39640.1	gamma-glutamyl transpeptidase 1	6,302	9,5568	5,9646	7,3
AT4G18100.1	Ribosomal protein L32e	7,7337	6,779	7,2787	7,3
AT2G09990.1	Ribosomal protein S5 domain 2-like superfamily protein	6,9814	6,4885	8,2507	7,2
AT4G20130.1	plastid transcriptionally active 14	8,0032	6,5263	7,0842	7,2
AT4G15000.1	Ribosomal L27e protein family	7,7069	6,6796	7,0066	7,1
AT5G54640.1	Histone superfamily protein	0,4934	5,1751	15,5368	7,1
AT1G77940.1	Ribosomal protein L7Ae/L30e/S12e/Gadd45 family protein	7,4203	6,9012	6,7243	7,0
AT1G52300.1	Zinc-binding ribosomal protein family protein	9,1502	5,79	6,05	7,0
AT5G49910.1	chloroplast heat shock protein 70-2	7,9344	6,6241	6,3269	7,0
AT3G47470.1	light-harvesting chlorophyll-protein complex I subunit A4	7,7305	6,3966	6,6025	6,9
ATCG01020.1	ribosomal protein L32	8,33	6,1931	6,1104	6,9
AT1G35680.1	Ribosomal protein L21	7,484	6,1164	6,8578	6,8
AT5G22640.1	MORN (Membrane Occupation and Recognition Nexus) repeat-containing protein	6,5671	6,6929	7,1606	6,8
AT2G36160.1	Ribosomal protein S11 family protein	1,8881	9,0687	9,3938	6,8
AT2G15290.1	translocon at inner membrane of chloroplasts 21	4,3467	7,8603	8,0515	6,8
AT1G07920.1	GTP binding Elongation factor Tu family protein	7,735	6,0947	6,3864	6,7
AT5G61170.1	Ribosomal protein S19e family protein	7,2261	6,1409	6,8165	6,7
AT5G23900.1	Ribosomal protein L13e family protein	7,3225	6,6834	6,0205	6,7
AT1G15820.1	light harvesting complex photosystem II subunit 6	6,8698	6,3396	6,6527	6,6

AT2G43630.1	FUNCTIONS IN: molecular_function unknown; INVOLVED IN: biological_process unknown; LOCATED IN: chloroplast thylakoid membrane, chloroplast, nucleus, chloroplast envelope; EXPRESSED IN: 22 plant structures; EXPRESSED DURING: 13 growth stages; BEST Arabidopsis thaliana protein match is: glycine-rich protein (TAIR:AT3G59640.2); Has 67 Blast hits to 67 proteins in 20 species: Archae - 0; Bacteria - 4; Metazoa - 9; Fungi - 1; Plants - 49; Viruses - 2; Other Eukaryotes - 2 (source: NCBI BLink).	9,3084	5,1911	5,2166	6,6
AT2G30620.2	winged-helix DNA-binding transcription factor family protein	7,1891	6,1818	6,264	6,5
AT1G75350.1	Ribosomal protein L31	7,0699	5,9515	6,442	6,5
AT1G58983.1	Ribosomal protein S5 family protein	7,6708	3,5322	8,2125	6,5
AT2G27720.1	60S acidic ribosomal protein family	6,5224	6,3872	6,4706	6,5
AT3G47520.1	malate dehydrogenase	6,4808	6,1213	6,6276	6,4
AT2G05220.1	Ribosomal S17 family protein	7,2063	4,8863	6,9306	6,3
AT1G17220.1	Translation initiation factor 2, small GTP-binding protein	5,9334	6,5411	6,515	6,3
AT3G25520.1	ribosomal protein L5	6,9987	5,885	5,8746	6,3
AT1G04270.2	cytosolic ribosomal protein S15	6,8833	5,4042	6,4459	6,2
AT3G12800.1	short-chain dehydrogenase-reductase B	6,8108	5,9913	5,5765	6,1
AT2G43950.1	chloroplast outer envelope protein 37	5,7592	6,236	6,3803	6,1
AT2G27710.1	60S acidic ribosomal protein family	7,1915	3,607	7,5597	6,1
AT1G69200.1	fructokinase-like 2	6,9766	5,4988	5,7181	6,1
AT3G22230.1	Ribosomal L27e protein family	6,533	5,7611	5,8675	6,1
AT4G34670.1	Ribosomal protein S3Ae	6,1315	6,7412	5,1876	6,0
AT1G08360.1	Ribosomal protein L1p/L10e family	6,4149	5,5641	5,9874	6,0

AT1G70070.1	DEAD/DEAH box helicase, putative	5,7872	6,0481	5,9569	5,9
AT5G38430.1	Ribulose bisphosphate carboxylase (small chain) family protein	5,2569	5,5146	6,9793	5,9
AT1G79040.1	photosystem II subunit R	3,5131	2,2815	11,8305	5,9
AT1G06760.1	winged-helix DNA-binding transcription factor family protein	6,0806	5,6786	5,7314	5,8
AT1G61580.1	R-protein L3 B	6,1209	5,4697	5,5526	5,7
AT2G27530.1	Ribosomal protein L1p/L10e family	6,154	4,9516	5,9889	5,7
AT1G72150.1	PATELLIN 1	5,4999	5,7358	5,7974	5,7
AT5G65250.1	unknown protein; FUNCTIONS IN: molecular_function unknown; INVOLVED IN: biological_process unknown; LOCATED IN: chloroplast; EXPRESSED IN: 22 plant structures; EXPRESSED DURING: 13 growth stages; Has 30201 Blast hits to 17322 proteins in 780 species: Archae - 12; Bacteria - 1396; Metazoa - 17338; Fungi - 3422; Plants - 5037; Viruses - 0; Other Eukaryotes - 2996 (source: NCBI BLink).	5,9694	4,6124	6,3692	5,7
AT5G01530.1	light harvesting complex photosystem II	5,8047	5,48	5,4859	5,6
AT1G78630.1	Ribosomal protein L13 family protein	6,1205	4,7048	5,7411	5,5
AT5G48760.1	Ribosomal protein L13 family protein	0,8405	0,9469	14,776	5,5
AT1G30380.1	photosystem I subunit K	5,3826	4,1055	6,9188	5,5
AT3G18420.1	Protein prenylyltransferase superfamily protein	2,5704	3,0539	10,377	5,3
AT4G27440.1	protochlorophyllide oxidoreductase B	5,5349	5,1253	5,3189	5,3
AT3G02560.1	Ribosomal protein S7e family protein	8,6654	2,649	4,3866	5,2
AT2G05620.1	proton gradient regulation 5	3,3761	6,5305	5,7768	5,2
AT3G26650.1	glyceraldehyde 3-phosphate dehydrogenase A subunit	5,6589	4,9823	4,6786	5,1
AT2G28830.1	PLANT U-BOX 12	8,1469	1,3893	5,7446	5,1
ATCG00280.1	photosystem II reaction center protein C	6,3462	4,3815	4,451	5,1
AT4G24280.1	chloroplast heat shock protein 70-1	4,597	4,7249	4,8741	4,7

AT1G52740.1	histone H2A protein 9	8,2778	0,4153	5,3789	4,7
AT2G24230.1	Leucine-rich repeat protein kinase family protein	7,6013	6,3257	0	4,6
ATCG00660.1	ribosomal protein L20	5,1229	3,6885	5,1015	4,6
AT3G13580.3	Ribosomal protein L30/L7 family protein	2,425	9,3151	2,0199	4,6
AT4G23940.1	FtsH extracellular protease family	3,0328	6,939	3,6864	4,6
AT5G24314.1	plastid transcriptionally active7	5,3195	4,0541	4,2617	4,5
AT1G02780.1	Ribosomal protein L19e family protein	5,1615	4,2374	4,1649	4,5
AT1G61520.2	photosystem I light harvesting complex gene 3	7,7471	5,7984	0	4,5
AT4G31780.2	monogalactosyl diacylglycerol synthase 1	5,3219	4,0784	4,1251	4,5
AT1G09590.1	Translation protein SH3-like family protein	4,7571	4,414	4,32	4,5
AT2G31610.1	Ribosomal protein S3 family protein	4,2414	4,4167	4,5392	4,4
AT3G51140.1	Protein of unknown function (DUF3353)	4,1348	4,9097	4,0885	4,4
AT1G31410.1	putrescine-binding periplasmic protein-related	1,5757	9,2094	2,3292	4,4
AT5G13510.1	Ribosomal protein L10 family protein	4,8801	3,9648	4,2196	4,4
AT2G28000.1	chaperonin-60alpha	4,3519	4,4935	3,9229	4,3
AT1G79560.1	FTSH protease 12	3,3603	4,7347	4,6254	4,2
AT3G53740.1	Ribosomal protein L36e family protein	1,9092	9,1343	1,6293	4,2
AT5G63420.1	RNA-metabolising metallo-beta-lactamase family protein	4,0392	4,0826	4,0423	4,1
AT3G54890.4	photosystem I light harvesting complex gene 1	3,7214	4,3306	4,1002	4,1
AT2G20450.1	Ribosomal protein L14	4,0942	4,042	3,9559	4,0
AT2G39390.1	Ribosomal L29 family protein	3,3699	4,0806	4,3813	3,9
AT4G09730.1	RH39	1,9792	4,8338	5,0164	3,9
AT3G56340.1	Ribosomal protein S26e family protein	3,7997	4,0398	3,8892	3,9
AT5G23310.1	Fe superoxide dismutase 3	4,0696	3,6867	3,9267	3,9
AT3G60245.1	Zinc-binding ribosomal protein family protein	3,9721	3,7336	3,912	3,9
AT1G41880.1	Ribosomal protein L35Ae family protein	3,7528	3,8213	3,9601	3,8
AT4G01037.1	Ubiquitin carboxyl-terminal hydrolase family protein	3,873	3,7339	3,9249	3,8
AT1G34430.1	2-oxoacid dehydrogenases acyltransferase family protein	5,0919	5,1173	1,0977	3,8

AT2G38140.1	plastid-specific ribosomal protein 4	4,0267	3,7695	3,4484	3,7
AT5G39850.1	Ribosomal protein S4	1,1019	6,442	3,3612	3,6
ATCG00470.1	ATP synthase epsilon chain	4,1388	3,6973	2,7898	3,5
AT4G30720.1	FAD/NAD(P)-binding oxidoreductase family protein	1,8426	0,9775	7,801	3,5
AT4G03280.2	photosynthetic electron transfer C	3,6525	3,387	3,4955	3,5
AT5G43745.1	Protein of unknown function (DUF1012)	5,2023	2,6567	2,6628	3,5
AT2G26540.1	uroporphyrinogen-III synthase family protein	3,6383	3,4231	3,451	3,5
AT4G39040.2	RNA-binding CRS1 / YhbY (CRM) domain protein	3,3109	3,7809	3,37	3,5
AT5G59500.1	protein C-terminal S-isoprenylcysteine carboxyl O-methyltransferases	8,8445	0,8164	0,7843	3,5
AT2G37190.1	Ribosomal protein L11 family protein	4,078	3,2994	3,0546	3,5
AT4G00100.1	ribosomal protein S13A	3,5012	3,4459	3,4355	3,5
AT1G23410.1	Ribosomal protein S27a / Ubiquitin family protein	3,5077	3,4921	3,3078	3,4
AT1G21600.1	plastid transcriptionally active 6	3,7219	3,1608	3,3387	3,4
AT3G53870.1	Ribosomal protein S3 family protein	3,6836	2,8112	3,6488	3,4
AT2G39730.1	rubisco activase	5,7894	4,2978	0	3,4
AT4G37510.1	Ribonuclease III family protein	4,1955	2,4435	3,2568	3,3
AT1G68830.1	STT7 homolog STN7	3,4821	3,1849	3,2043	3,3
AT4G02990.1	Mitochondrial transcription termination factor family protein	3,6575	3,0399	3,156	3,3
AT3G23390.1	Zinc-binding ribosomal protein family protein	3,9281	3,6729	2,2042	3,3
AT3G06510.1	Glycosyl hydrolase superfamily protein	3,6415	3,0547	3,0379	3,2
AT2G32650.1	RmlC-like cupins superfamily protein	3,4657	3,0344	3,1971	3,2
AT3G60770.1	Ribosomal protein S13/S15	3,135	3,0896	3,4445	3,2
ATCG00680.1	photosystem II reaction center protein B	3,784	2,9886	2,8765	3,2
AT3G12580.1	heat shock protein 70	4,5539	1,5197	3,5727	3,2
AT3G61070.1	peroxin 11E	4,9607	4,6785	0	3,2
AT2G47610.1	Ribosomal protein L7Ae/L30e/S12e/Gadd45 family protein	4,3823	2,1716	2,9223	3,2
AT4G36130.1	Ribosomal protein L2 family	3,3576	3,2722	2,7512	3,1
AT5G57290.2	60S acidic ribosomal protein family	2,9975	3,2344	3,1274	3,1

AT5G64580.1	AAA-type ATPase family protein	2,9517	3,093	3,1265	3,1
AT3G12390.1	Nascent polypeptide-associated complex (NAC), alpha subunit family protein	3,125	2,5043	3,5204	3,0
AT1G44575.1	Chlorophyll A-B binding family protein	3,9578	2,5521	2,5594	3,0
AT3G16290.1	AAA-type ATPase family protein	4,341	2,4045	2,2805	3,0
AT1G76360.1	Protein kinase superfamily protein	1,589	2,1628	5,1688	3,0
AT1G01100.1	60S acidic ribosomal protein family	2,9904	2,9803	2,7704	2,9
AT1G08520.1	ALBINA 1	2,8799	2,8833	2,8951	2,9
AT3G04650.1	FAD/NAD(P)-binding oxidoreductase family protein	2,8351	2,8437	2,941	2,9
AT3G28900.1	Ribosomal protein L34e superfamily protein	0	4,9453	3,6605	2,9
AT1G48830.1	Ribosomal protein S7e family protein	2,6425	2,815	3,1025	2,9
AT5G35530.1	Ribosomal protein S3 family protein	2,6202	3,9534	1,8413	2,8
AT3G04340.1	FtsH extracellular protease family	2,5869	2,8551	2,963	2,8
AT3G18740.1	Ribosomal protein L7Ae/L30e/S12e/Gadd45 family protein	0,74	6,9012	0,7493	2,8
ATCG00020.1	photosystem II reaction center protein A	3,759	2,3183	2,3039	2,8
AT4G34620.1	small subunit ribosomal protein 16	2,8677	2,7273	2,7201	2,8
AT3G25860.1	2-oxoacid dehydrogenases acyltransferase family protein	2,6744	2,6674	2,7798	2,7
AT3G53020.1	Ribosomal protein L24e family protein	3,9395	0	4,0985	2,7
AT2G43460.1	Ribosomal L38e protein family	3,0142	2,1202	2,9009	2,7
AT3G59780.1	Rhodanese/Cell cycle control phosphatase superfamily protein	3,6656	2,0427	2,222	2,6
AT2G19870.1	tRNA/rRNA methyltransferase (SpoU) family protein	2,858	2,4284	2,6324	2,6
AT3G04760.1	Pentatricopeptide repeat (PPR-like) superfamily protein	0	0,7609	7,1548	2,6
ATCG00270.1	photosystem II reaction center protein D	2,751	2,5281	2,6339	2,6
AT1G16720.1	high chlorophyll fluorescence phenotype 173	2,861	2,4982	2,4501	2,6

AT5G62270.1	BEST Arabidopsis thaliana protein match is: mucin-related (TAIR:AT2G02880.1); Has 35333 Blast hits to 34131 proteins in 2444 species: Archae - 798; Bacteria - 22429; Metazoa - 974; Fungi - 991; Plants - 531; Viruses - 0; Other Eukaryotes - 9610 (source: NCBI BLink).	3,9917	3,3409	0,4646	2,6
AT2G21580.2	Ribosomal protein S25 family protein	2,883	2,2572	2,4291	2,5
AT1G02150.1	Tetratricopeptide repeat (TPR)-like superfamily protein	2,9116	2,3961	2,2393	2,5
AT1G10590.1	Nucleic acid-binding, OB-fold-like protein	2,8352	1,5971	3,0617	2,5
AT5G15520.1	Ribosomal protein S19e family protein	2,7536	2,1573	2,5781	2,5
AT4G13170.1	Ribosomal protein L13 family protein	0	3,9147	3,5438	2,5
AT3G12780.1	phosphoglycerate kinase 1	2,3572	2,5201	2,5358	2,5
AT2G24820.1	translocon at the inner envelope membrane of chloroplasts 55-II	2,5855	2,4715	2,3394	2,5
AT4G09650.1	ATP synthase delta-subunit gene	2,9219	2,2596	2,2098	2,5
AT5G13850.1	nascent polypeptide-associated complex subunit alpha-like protein 3	2,2085	3,1821	1,912	2,4
AT4G40030.1	Histone superfamily protein	0	3,4948	3,7083	2,4
AT5G26780.1	serine hydroxymethyltransferase 2	0,5799	5,8453	0,6938	2,4
AT1G17880.1	basic transcription factor 3	2,7893	2,0387	2,2052	2,3
AT3G61260.1	Remorin family protein	2,6423	2,1838	2,1868	2,3
AT4G24190.1	Chaperone protein htpG family protein	2,256	2,1852	2,5254	2,3
ATCG00540.1	photosynthetic electron transfer A	2,4546	2,1814	2,2965	2,3
AT1G20340.1	Cupredoxin superfamily protein	2,2167	2,2814	2,4236	2,3
AT2G19740.1	Ribosomal protein L31e family protein	3,8736	0	2,9155	2,3
AT1G80410.1	tetratricopeptide repeat (TPR)-containing protein	1,3078	3,3123	2,15	2,3
AT3G02530.1	TCP-1/cpn60 chaperonin family protein	2,4263	2,5582	1,7351	2,2
AT4G10480.1	Nascent polypeptide-associated complex (NAC), alpha subunit family protein	2,1031	2,1892	2,283	2,2

AT3G46740.1	translocon at the outer envelope membrane of chloroplasts 75-III	2,277	2,1421	2,1107	2,2
AT1G74470.1	Pyridine nucleotide-disulphide oxidoreductase family protein	1,5927	3,2249	1,7115	2,2
ATCG00130.1	ATPase, F0 complex, subunit B/B', bacterial/chloroplast	2,9988	1,724	1,7811	2,2
AT1G16790.1	ribosomal protein-related	2,3286	2,0945	2,0718	2,2
AT5G46290.2	3-ketoacyl-acyl carrier protein synthase I	2	2,2761	2,1805	2,2
AT5G46420.1	16S rRNA processing protein RimM family	2,4398	1,8648	2,1283	2,1
AT2G37400.1	Tetratricopeptide repeat (TPR)-like superfamily protein	2,1615	2,2164	2,0476	2,1
AT3G10090.1	Nucleic acid-binding, OB-fold-like protein	2,0814	1,9593	2,3769	2,1
AT2G05100.1	photosystem II light harvesting complex gene 2.1	2,768	1,3328	2,3142	2,1
AT3G09680.1	Ribosomal protein S12/S23 family protein	0	2,9534	3,4073	2,1
AT3G20230.1	Ribosomal L18p/L5e family protein	2,0387	2,3272	1,9592	2,1
AT2G45820.1	Remorin family protein	2,1803	2,0435	2,0907	2,1
AT3G53560.1	Tetratricopeptide repeat (TPR)-like superfamily protein	1,9964	2,0678	2,184	2,1
AT1G03630.2	protochlorophyllide oxidoreductase C	2,2329	1,9769	1,9773	2,1
AT5G08050.1	Protein of unknown function (DUF1118)	2,1816	1,9909	2,0112	2,1
AT3G57180.1	P-loop containing nucleoside triphosphate hydrolases superfamily protein	2,1886	1,9164	1,9685	2,0
AT1G01790.1	K+ efflux antiporter 1	2,0146	2,0617	1,9955	2,0
AT3G46780.1	plastid transcriptionally active 16	1,8313	1,8769	2,326	2,0
AT4G28750.1	Photosystem I reaction centre subunit IV / PsaE protein	3,1895	2,8382	0	2,0
AT3G50820.1	photosystem II subunit O-2	1,3311	1,2491	3,4461	2,0
AT1G56110.1	homolog of nucleolar protein NOP56	1,9593	2,0051	2,0226	2,0
AT3G14600.1	Ribosomal protein L18ae/LX family protein	2,5429	1,6907	1,7117	2,0
AT5G50920.1	CLPC homologue 1	1,9433	1,9153	2,0385	2,0
AT5G02500.2	heat shock cognate protein 70-1	0	2,9987	2,8767	2,0

AT3G09210.1	plastid transcriptionally active 13	2,8784	1,4794	1,5049	2,0
AT3G49470.1	nascent polypeptide-associated complex subunit alpha-like protein 2	3,6503	1,0186	1,1204	1,9
AT4G32260.1	ATPase, F0 complex, subunit B/B', bacterial/chloroplast	2,0875	1,8017	1,8982	1,9
AT5G66190.1	ferredoxin-NADP(+)-oxidoreductase 1	2,0761	1,8352	1,8298	1,9
AT5G54180.1	plastid transcriptionally active 15	1,1497	1,0301	3,5042	1,9
AT3G04400.2	Ribosomal protein L14p/L23e family protein	1,9027	1,8492	1,8519	1,9
AT5G53860.1	embryo defective 2737	2,2575	1,6328	1,6547	1,8
AT5G61780.1	TUDOR-SN protein 2	0,5716	4,0927	0,8502	1,8
AT5G46430.1	Ribosomal protein L32e	1,9731	1,6905	1,8389	1,8
AT4G21280.1	photosystem II subunit QA	1,6742	2,028	1,7942	1,8
AT2G45740.1	peroxin 11D	1,8032	1,7613	1,9108	1,8
AT4G16390.1	pentatricopeptide (PPR) repeat-containing protein	1,8607	1,7293	1,8428	1,8
AT5G04130.1	DNA GYRASE B2	2,0035	2,1483	1,2007	1,8
AT4G35250.1	NAD(P)-binding Rossmann-fold superfamily protein	2,0025	1,5671	1,5974	1,7
AT3G11940.1	ribosomal protein 5A	1,8459	1,498	1,8008	1,7
AT3G02450.1	cell division protein ftsH, putative	0	3,2247	1,8989	1,7
AT5G18660.1	NAD(P)-binding Rossmann-fold superfamily protein	0,9965	3,2576	0,8555	1,7
AT2G24060.1	Translation initiation factor 3 protein	1,9116	1,575	1,6037	1,7
AT1G55490.1	chaperonin 60 beta	1,6026	1,7254	1,6538	1,7
AT4G05180.1	photosystem II subunit Q-2	1,9096	1,9924	1,0165	1,6
AT3G10270.1	DNA GYRASE B1	1,4858	1,6466	1,7435	1,6
AT1G73230.1	Nascent polypeptide-associated complex NAC	1,7703	1,3886	1,7074	1,6
AT5G10910.1	mraW methylase family protein	1,0344	3,0432	0,7691	1,6
ATCG00040.1	maturase K	0	2,4181	2,373	1,6
AT1G48350.1	Ribosomal L18p/L5e family protein	1,2812	1,7905	1,7001	1,6
AT3G20050.1	T-complex protein 1 alpha subunit	1,0413	3,1033	0,6161	1,6
AT4G29410.1	Ribosomal L28e protein family	1,5187	1,8528	1,373	1,6
AT4G30690.1	Translation initiation factor 3 protein	1,8138	0,8228	2,0051	1,5
ATCG00340.1	Photosystem I, PsaA/PsaB protein	1,4953	1,5732	1,5576	1,5
AT1G29250.1	Alba DNA/RNA-binding protein	1,9616	1,4232	1,2093	1,5

AT3G17170.1	Translation elongation factor EF1B/ribosomal protein S6 family protein	1,6318	1,5158	1,4027	1,5
AT5G07030.1	Eukaryotic aspartyl protease family protein	1,7319	1,4096	1,3731	1,5
AT5G04590.1	sulfite reductase	1,484	0	2,9161	1,5
AT2G25830.1	YebC-related	0,8374	2,4448	1,111	1,5
AT5G53170.1	FTSH protease 11	0,9832	2,4871	0,8899	1,5
AT5G64040.1	photosystem I reaction center subunit PSI-N, chloroplast, putative / PSI-N, putative (PSAN)	1,5625	1,4125	1,3677	1,4
AT5G08540.1	unknown protein; FUNCTIONS IN: molecular_function unknown; INVOLVED IN: biological_process unknown; LOCATED IN: chloroplast thylakoid membrane, chloroplast, chloroplast envelope; EXPRESSED IN: 24 plant structures; EXPRESSED DURING: 13 growth stages; Has 30201 Blast hits to 17322 proteins in 780 species: Archae - 12; Bacteria - 1396; Metazoa - 17338; Fungi - 3422; Plants - 5037; Viruses - 0; Other Eukaryotes - 2996 (source: NCBI BLink).	1,4922	1,2937	1,4541	1,4
AT1G56070.1	Ribosomal protein S5/Elongation factor G/III/V family protein	1,0556	2,7271	0,4319	1,4
AT2G24420.1	DNA repair ATPase-related	1,3866	1,3445	1,4807	1,4

AT1G54520.1	unknown protein; FUNCTIONS IN: molecular_function unknown; INVOLVED IN: biological_process unknown; LOCATED IN: chloroplast; EXPRESSED IN: 24 plant structures; EXPRESSED DURING: 15 growth stages; CONTAINS InterPro DOMAIN/s: Protein of unknown function DUF1517 (InterPro:IPR010903); Has 276 Blast hits to 275 proteins in 83 species: Archae - 0; Bacteria - 108; Metazoa - 6; Fungi - 0; Plants - 113; Viruses - 0; Other Eukaryotes - 49 (source: NCBI BLink).	1,4522	1,3191	1,4352	1,4
AT3G08940.2	light harvesting complex photosystem II	1,375	1,2293	1,5879	1,4
AT4G28210.1	embryo defective 1923	1,2855	1,4671	1,4099	1,4
AT3G18390.1	CRS1 / YhbY (CRM) domain-containing protein	1,2918	1,7383	1,0842	1,4
AT1G48610.1	AT hook motif-containing protein	1,6111	1,1117	1,3834	1,4
AT1G29965.1	Ribosomal protein L18ae/LX family protein	1,6421	1,2764	1,1856	1,4
AT1G10522.1	unknown protein; FUNCTIONS IN: molecular_function unknown; INVOLVED IN: biological_process unknown; LOCATED IN: chloroplast; EXPRESSED IN: 23 plant structures; EXPRESSED DURING: 13 growth stages; Has 24 Blast hits to 24 proteins in 8 species: Archae - 0; Bacteria - 0; Metazoa - 0; Fungi - 0; Plants - 24; Viruses - 0; Other Eukaryotes - 0 (source: NCBI BLink).	2,5562	0	1,541	1,4
AT3G01500.1	carbonic anhydrase 1	0	1,9757	2,1004	1,4
AT4G25630.1	fibrillarin 2	2,1597	0	1,9152	1,4
AT5G46580.1	pentatricopeptide (PPR) repeat-containing protein	1,406	1,2827	1,3731	1,4
AT3G21200.1	proton gradient regulation 7	1,0728	0,8209	2,1544	1,3

AT1G09795.1	ATP phosphoribosyl transferase 2	1,0868	2,5522	0,3333	1,3
AT1G80480.1	plastid transcriptionally active 17	0,9421	0,6449	2,355	1,3
AT2G46110.1	ketopantoate hydroxymethyltransferase 1	1,3783	1,4028	1,1538	1,3
AT2G04270.1	RNAse E/G-like	1,1706	1,5011	1,2511	1,3
AT1G67730.1	beta-ketoacyl reductase 1	1,5744	1,1691	1,1623	1,3
AT2G33430.1	differentiation and greening-like 1	0,9801	0,7831	2,04	1,3
AT5G60670.1	Ribosomal protein L11 family protein	1,1505	1,1651	1,4834	1,3
AT1G03130.1	photosystem I subunit D-2	1,4656	1,034	1,2798	1,3
AT2G23070.1	Protein kinase superfamily protein	1,1983	1,2604	1,2482	1,2
AT2G40800.1	unknown protein; BEST Arabidopsis thaliana protein match is: unknown protein (TAIR:AT3G56430.1); Has 43 Blast hits to 43 proteins in 15 species: Archae - 0; Bacteria - 0; Metazoa - 0; Fungi - 0; Plants - 41; Viruses - 0; Other Eukaryotes - 2 (source: NCBI BLink).	1,3499	1,0975	1,0638	1,2
AT5G22830.1	magnesium (Mg) transporter 10	1,1958	1,116	1,1712	1,2
AT2G37600.1	Ribosomal protein L36e family protein	0,1573	3,3048	0	1,2
AT5G08180.1	Ribosomal protein L7Ae/L30e/S12e/Gadd45 family protein	1,1039	1,0929	1,2136	1,1
AT5G14460.1	Pseudouridine synthase family protein	0,7456	1,2062	1,4515	1,1
AT5G08650.1	Small GTP-binding protein	1,1532	1,0856	1,1423	1,1
AT3G23300.1	S-adenosyl-L-methionine-dependent methyltransferases superfamily protein	1,8332	0	1,5479	1,1
AT5G17170.1	rubredoxin family protein	0	1,4855	1,8821	1,1
ATCG00720.1	photosynthetic electron transfer B	1,06	1,0529	1,1856	1,1
AT3G26060.1	Thioredoxin superfamily protein	1,2781	0,9701	1,0395	1,1
AT3G48960.1	Ribosomal protein L13e family protein	1,8661	0	1,4179	1,1
AT5G47320.1	ribosomal protein S19	0,5027	2,1653	0,5928	1,1
AT1G12900.3	glyceraldehyde 3-phosphate dehydrogenase A subunit 2	1,8945	1,3275	0	1,1
AT3G12080.1	GTP-binding family protein	1,0458	1,0716	1,0924	1,1

AT1G76010.1	Alba DNA/RNA-binding protein	0,8838	1,0097	1,2884	1,1
AT5G19690.1	staurosporin and temperature sensitive 3-like A	1,195	0,9834	0,9898	1,1
AT5G13650.1	elongation factor family protein	1,7389	0,7849	0,6369	1,1
AT1G04170.1	eukaryotic translation initiation factor 2 gamma subunit	1,2586	0,9609	0,9282	1,0
AT4G39960.1	Molecular chaperone Hsp40/DnaJ family protein	1,0941	0,89	1,0833	1,0
AT4G38970.1	fructose-bisphosphate aldolase 2	0,8828	1,541	0,621	1,0
AT1G30360.1	Early-responsive to dehydration stress protein (ERD4)	0,9893	0,9383	1,1095	1,0
AT1G33040.1	nascent polypeptide-associated complex subunit alpha-like protein 5	1,2864	0,9528	0,7961	1,0
AT3G16950.1	lipoamide dehydrogenase 1	0,5207	1,4742	1,032	1,0
AT1G15140.1	FAD/NAD(P)-binding oxidoreductase	1,1836	0,8995	0,9355	1,0
AT4G27500.1	proton pump interactor 1	1,0787	0,9142	0,9872	1,0
AT5G16715.1	ATP binding;valine-tRNA ligases;aminoacyl-tRNA ligases;nucleotide binding;ATP binding;aminoacyl-tRNA ligases	2,0545	0	0,8824	1,0

AT3G49720.1	unknown protein; FUNCTIONS IN: molecular_function unknown; INVOLVED IN: biological_process unknown; LOCATED IN: chloroplast thylakoid membrane, Golgi apparatus, plasma membrane, membrane; EXPRESSED IN: 25 plant structures; EXPRESSED DURING: 15 growth stages; BEST Arabidopsis thaliana protein match is: unknown protein (TAIR:AT5G65810.1); Has 64 Blast hits to 64 proteins in 11 species: Archae - 0; Bacteria - 0; Metazoa - 0; Fungi - 0; Plants - 64; Viruses - 0; Other Eukaryotes - 0 (source: NCBI BLink).	0,9184	0,9885	1,0184	1,0
AT5G39740.1	ribosomal protein L5 B	1,0991	0,9607	0,8492	1,0
AT1G54780.1	thylakoid lumen 18.3 kDa protein	0	2,5219	0,3696	1,0
AT3G20440.1	Alpha amylase family protein	1,1373	1	0,6996	0,9
AT1G58290.1	Glutamyl-tRNA reductase family protein	1,1009	0,8478	0,8803	0,9
AT2G47470.2	thioredoxin family protein	0,5632	0,4757	1,7864	0,9
AT5G03900.1	Iron-sulphur cluster biosynthesis family protein	0,9098	0,971	0,9417	0,9
AT2G38270.1	CAX-interacting protein 2	1,6046	0,605	0,6121	0,9
AT3G27180.1	S-adenosyl-L-methionine-dependent methyltransferases superfamily protein	1,0849	0,831	0,8964	0,9
AT5G42080.1	dynamin-like protein	0	1,4121	1,3918	0,9
AT3G05060.1	NOP56-like pre RNA processing ribonucleoprotein	0,9986	0,8578	0,8975	0,9
AT1G80030.1	Molecular chaperone Hsp40/DnaJ family protein	0,9119	0,8903	0,9021	0,9
AT4G16155.1	dihydrolipoyl dehydrogenases	0,6077	1,4974	0,5518	0,9
AT5G58870.1	FTSH protease 9	0,7892	0,2853	1,5502	0,9

AT3G56430.1	unknown protein; FUNCTIONS IN: molecular_function unknown; INVOLVED IN: biological_process unknown; LOCATED IN: mitochondrion, vacuole; EXPRESSED IN: 22 plant structures; EXPRESSED DURING: 13 growth stages; BEST Arabidopsis thaliana protein match is: unknown protein (TAIR:AT2G40800.1); Has 3121 Blast hits to 1477 proteins in 196 species: Archae - 12; Bacteria - 170; Metazoa - 996; Fungi - 324; Plants - 132; Viruses - 59; Other Eukaryotes - 1428 (source: NCBI BLink).	0,8165	0,8098	0,9796	0,9
AT5G42020.1	Heat shock protein 70 (Hsp 70) family protein	1,3786	0	1,2139	0,9
AT1G06680.2	photosystem II subunit P-1	0,7959	0,9143	0,8573	0,9
AT5G30510.1	ribosomal protein S1	0,9013	0,8657	0,792	0,9
AT4G02510.1	translocon at the outer envelope membrane of chloroplasts 159	1,3854	1,1658	0	0,9
AT2G31810.1	ACT domain-containing small subunit of acetolactate synthase protein	0,9442	0,829	0,7773	0,9
AT1G59990.1	DEA(D/H)-box RNA helicase family protein	0,7553	0,9737	0,8106	0,8
AT3G11630.1	Thioredoxin superfamily protein	1,3866	0	1,136	0,8
AT3G12930.1	Lojap-related protein	0,8752	0,7998	0,8148	0,8
AT2G04520.1	Nucleic acid-binding, OB-fold-like protein	0,7931	0,9741	0,7144	0,8
AT1G54270.2	eif4a-2	0,8429	0,755	0,7774	0,8
AT5G66570.1	PS II oxygen-evolving complex 1	0,8798	0,9189	0,571	0,8
AT3G56940.1	dicarboxylate diiron protein, putative (Crd1)	0,6842	0,8391	0,8337	0,8
AT1G59610.1	dynamin-like 3	0,3493	0,3626	1,6151	0,8
AT1G06950.1	translocon at the inner envelope membrane of chloroplasts 110	0,4361	1,0378	0,8471	0,8
AT5G24850.1	cryptochrome 3	0,8954	0,5432	0,8716	0,8
AT1G10290.1	dynamin-like protein 6	1,8475	0,4102	0	0,8
AT5G23120.1	photosystem II stability/assembly factor, chloroplast (HCF136)	0,7014	0,7499	0,7537	0,7

AT5G46630.1	Clathrin adaptor complexes medium subunit family protein	0,8128	0,6597	0,7312	0,7
AT3G11830.1	TCP-1/cpn60 chaperonin family protein	0,775	0,7116	0,6862	0,7
AT5G11480.1	P-loop containing nucleoside triphosphate hydrolases superfamily protein	0,7722	0,6919	0,7077	0,7
AT5G14800.1	pyrroline-5- carboxylate (P5C) reductase	0,9597	0,5958	0,5854	0,7
AT1G26910.1	Ribosomal protein L16p/L10e family protein	0	1,1428	0,988	0,7
AT5G06290.1	2-cysteine peroxiredoxin B	0,2787	1,5058	0,3298	0,7
AT5G08670.1	ATP synthase alpha/beta family protein	0,5421	0,7599	0,7715	0,7
AT4G15790.1	unknown protein; INVOLVED IN: biological_process unknown; LOCATED IN: chloroplast; EXPRESSED IN: 21 plant structures; EXPRESSED DURING: 13 growth stages; Has 411 Blast hits to 380 proteins in 121 species: Archae - 4; Bacteria - 107; Metazoa - 85; Fungi - 29; Plants - 59; Viruses - 0; Other Eukaryotes - 127 (source: NCBI BLink).	0,9368	0	1,1068	0,7
AT4G00810.1	60S acidic ribosomal protein family	0,8469	0,5369	0,657	0,7
AT1G75780.1	tubulin beta-1 chain	0,9184	0,6058	0,5012	0,7
AT3G20320.1	trigalactosyldiacylglycerol2	0,6271	0,6654	0,709	0,7
AT5G24490.1	30S ribosomal protein, putative	1,1127	0,8743	0	0,7
AT1G56190.2	Phosphoglycerate kinase family protein	0,5639	0,6888	0,7342	0,7
AT4G21150.2	ribophorin II (RPN2) family protein	0,6566	0,6603	0,6167	0,6
AT3G18080.1	B-S glucosidase 44	0,8265	0,5247	0,5443	0,6
AT2G22360.1	DNAJ heat shock family protein	0,7288	0,5383	0,6261	0,6
AT1G20020.2	ferredoxin-NADP(+)-oxidoreductase 2	0,8619	0,9973	0	0,6
AT5G20920.1	eukaryotic translation initiation factor 2 beta subunit	0,798	0	1,0301	0,6
AT4G25050.1	acyl carrier protein 4	0,6517	0,4191	0,7206	0,6
AT3G03920.1	H/ACA ribonucleoprotein complex, subunit Gar1/Naf1 protein	0,6383	0,563	0,5892	0,6

AT3G52180.1	dual specificity protein phosphatase (DsPTP1) family protein	0,558	0,5678	0,6644	0,6
AT1G66520.1	formyltransferase, putative	0,5933	0,6308	0,5348	0,6
AT4G01050.1	thylakoid rhodanese-like	1,0061	0,7478	0	0,6
AT4G02230.1	Ribosomal protein L19e family protein	1,1143	0	0,6254	0,6
AT3G63410.1	S-adenosyl-L-methionine-dependent methyltransferases superfamily protein	0,6022	0,5361	0,5834	0,6
AT5G67510.1	Translation protein SH3-like family protein	0,4946	0,5483	0,6703	0,6
AT5G66680.1	dolichyl-diphosphooligosaccharide-protein glycosyltransferase 48kDa subunit family protein	0,517	0,5029	0,6138	0,5
AT5G47700.1	60S acidic ribosomal protein family	0,7383	0,4359	0,4326	0,5
AT1G76405.2	unknown protein; FUNCTIONS IN: molecular_function unknown; INVOLVED IN: biological_process unknown; LOCATED IN: chloroplast envelope; EXPRESSED IN: 21 plant structures; EXPRESSED DURING: 13 growth stages; BEST Arabidopsis thaliana protein match is: unknown protein (TAIR:AT1G20816.1); Has 52 Blast hits to 52 proteins in 19 species: Archae - 0; Bacteria - 0; Metazoa - 0; Fungi - 0; Plants - 50; Viruses - 0; Other Eukaryotes - 2 (source: NCBI BLink).	1	0,5981	0	0,5
AT5G01920.1	Protein kinase superfamily protein	0	0,6824	0,9099	0,5
AT5G12250.1	beta-6 tubulin	0,8327	0	0,2246	0,4
AT2G25080.1	glutathione peroxidase 1	0,7768	0	0,5946	0,5
AT5G54270.1	light-harvesting chlorophyll B-binding protein 3	0,709	0	0,6439	0,5
AT5G14910.1	Heavy metal transport/detoxification superfamily protein	0,6882	0,6232	0	0,4

AT5G19370.1	rhodanese-like domain-containing protein / PPIC-type PPIASE domain-containing protein	0,6613	0,4097	0,4175	0,5
AT1G31817.1	Ribosomal L18p/L5e family protein	0,649	0,3677	0,4524	0,5
AT1G49510.1	embryo defective 1273	0,6451	0,6018	0	0,4
AT3G18035.1	winged-helix DNA-binding transcription factor family protein	0,6174	0,81	0	0,5
AT5G58330.3	lactate/malate dehydrogenase family protein	0,5872	0	0,6156	0,4
AT4G11175.1	Nucleic acid-binding, OB-fold-like protein	0,57	0	0,3497	0,3
AT5G45930.1	magnesium chelatase i2	0,5699	0,4141	0,4386	0,5
AT4G14960.1	Tubulin/FtsZ family protein	0,5635	0	0,5984	0,4
AT3G61530.1	Phosphoenolpyruvate carboxylase family protein	0,5599	0,6037	0	0,4
AT2G24020.1	Uncharacterised BCR, YbaB family COG0718	0,5475	0	0,4409	0,3
AT1G71500.1	Rieske (2Fe-2S) domain-containing protein	0,5226	0	0,5693	0,4
ATCG01110.1	NAD(P)H dehydrogenase subunit H	0,4844	0,3507	0,3819	0,4
AT4G16180.2	unknown protein; FUNCTIONS IN: molecular_function unknown; INVOLVED IN: biological_process unknown; LOCATED IN: plasma membrane; BEST Arabidopsis thaliana protein match is: unknown protein (TAIR:AT3G28720.1); Has 5 Blast hits to 5 proteins in 3 species: Archae - 0; Bacteria - 0; Metazoa - 0; Fungi - 0; Plants - 4; Viruses - 0; Other Eukaryotes - 1 (source: NCBI BLink).	0,4777	0	0,4038	0,3
AT2G21660.2	cold, circadian rhythm, and rna binding 2	0,474	0	0,5254	0,3
AT1G04630.1	GRIM-19 protein	0,4739	0,3134	0	0,3
AT5G59240.1	Ribosomal protein S8e family protein	0,4696	0	0,5983	0,4
AT1G26630.1	Eukaryotic translation initiation factor 5A-1 (eIF-5A 1) protein	0,4586	0	0,4579	0,3

AT5G22210.1	unknown protein; FUNCTIONS IN: molecular_function unknown; INVOLVED IN: N-terminal protein myristoylation; LOCATED IN: cellular_component unknown; Has 30201 Blast hits to 17322 proteins in 780 species: Archae - 12; Bacteria - 1396; Metazoa - 17338; Fungi - 3422; Plants - 5037; Viruses - 0; Other Eukaryotes - 2996 (source: NCBI BLink).	0,4583	0	0,603	0,4
AT3G53420.1	plasma membrane intrinsic protein 2A	0,4362	0,405	0	0,3
AT1G12310.1	Calcium-binding EF-hand family protein	0,4324	0,3957	0,3886	0,4
AT1G67700.1	unknown protein; FUNCTIONS IN: molecular_function unknown; INVOLVED IN: biological_process unknown; LOCATED IN: chloroplast, chloroplast envelope; EXPRESSED IN: 22 plant structures; EXPRESSED DURING: 13 growth stages; Has 49 Blast hits to 49 proteins in 20 species: Archae - 0; Bacteria - 0; Metazoa - 0; Fungi - 0; Plants - 44; Viruses - 0; Other Eukaryotes - 5 (source: NCBI BLink).	0,4252	0,4764	0	0,3
AT2G40290.1	Eukaryotic translation initiation factor 2 subunit 1	0,4229	0,4506	0,5744	0,5
AT3G08740.1	elongation factor P (EF-P) family protein	0,4194	0,4491	0,4672	0,4
AT1G47260.1	gamma carbonic anhydrase 2	0,3908	0,4265	0	0,3
AT1G70490.1	Ras-related small GTP-binding family protein	0,389	0	0,237	0,2
AT2G30320.1	Pseudouridine synthase family protein	0,3871	0,3868	0,4458	0,4
AT3G09440.1	Heat shock protein 70 (Hsp 70) family protein	0,3815	0,3578	0,2812	0,3
AT3G07180.3	GPI transamidase component PIG-S-related	0,3801	0,3427	0	0,2
AT3G03630.1	cysteine synthase 26	0,3696	0,3851	0,4495	0,4

AT2G43750.1	O-acetylserine (thiol) lyase B	0,3652	0	0,5491	0,3
AT1G13440.2	glyceraldehyde-3-phosphate dehydrogenase C2	0,3535	0	0,625	0,3
AT1G33810.1	unknown protein; FUNCTIONS IN: molecular_function unknown; INVOLVED IN: biological_process unknown; LOCATED IN: chloroplast thylakoid membrane, chloroplast, chloroplast envelope; EXPRESSED IN: 22 plant structures; EXPRESSED DURING: 13 growth stages; Has 39 Blast hits to 39 proteins in 18 species: Archae - 0; Bacteria - 0; Metazoa - 0; Fungi - 0; Plants - 39; Viruses - 0; Other Eukaryotes - 0 (source: NCBI BLink).	0,3482	0	0,3609	0,2
AT1G19690.1	NAD(P)-binding Rossmann-fold superfamily protein	0,3444	0,2668	0	0,2
AT4G34555.1	Ribosomal protein S25 family protein	0,3256	0,2385	0	0,2
AT1G64880.1	Ribosomal protein S5 family protein	0,3248	0	0,3107	0,2
AT2G07640.1	NAD(P)-binding Rossmann-fold superfamily protein	0	0,8634	0,0772	0,3
AT3G02650.1	Tetratricopeptide repeat (TPR)-like superfamily protein	0	0,6033	0,6071	0,4
AT2G45710.1	Zinc-binding ribosomal protein family protein	0	0,55	0,6056	0,4
AT3G16780.1	Ribosomal protein L19e family protein	0	0,5401	0,7664	0,4
AT1G73940.1	unknown protein; BEST Arabidopsis thaliana protein match is: unknown protein (TAIR:AT5G49410.2); Has 54 Blast hits to 54 proteins in 11 species: Archae - 0; Bacteria - 0; Metazoa - 0; Fungi - 0; Plants - 54; Viruses - 0; Other Eukaryotes - 0 (source: NCBI BLink).	0	0,4526	0,5365	0,3
AT5G17990.1	tryptophan biosynthesis 1	0	0,4155	0,3914	0,3
AT4G35000.1	ascorbate peroxidase 3	0	0,3816	0,4917	0,3
AT3G18780.1	actin 2	0	0,374	0,2882	0,2

AT4G18480.1	P-loop containing nucleoside triphosphate hydrolases superfamily protein	0	0,3629	0,3643	0,2
AT2G28900.1	outer plastid envelope protein 16-1	0	0,3382	0,3888	0,2
AT3G48870.2	Clp ATPase	0	0,2625	0,1488	0,1
AT5G65840.1	Thioredoxin superfamily protein	0	0,2445	1,0233	0,4

Supplemental Table S5: List of peptides phosphorylated by recombinant pCKII and the isolated native plastid casein kinases from *Arabidopsis thaliana* (A.t.) and *Sinapis alba* (S.a.). The phosphorylation category was determined by calculating the grayscale intensity median of all spot pixels after background subtraction. These intensity values were averaged over all 9 peptide spots and classified into 4 categories. The categorries are defined as 3 - strong(>50% segregation of background intensity [BI]); 2- medium(>20% - <50% BI); 1 - weak(>10% - <20% BI); 0 – none(<10% BI).

Peptide	Identifier	Annotation	Phosphorylation category		
			recombinant pCKII	native enzyme	
				HepSeph- A.t.	HepSeph- S.a.
AFKSLGLSDHDEYDL	AT5G26742.1	emb1138 | DEAD box RNA	3	1	1
	AT5G26742.2	emb1138 | DEAD box RNA			
DASEGDVSEGDESEG	AT4G24770.1	RBP31, ATRBP31, CP31,	2	1	1
DEEEGNVSDRGDEDE	AT4G12610.1	RAP74, ATRAP74	1	1	0
	AT4G12610.2	RAP74, ATRAP74			
DNEVEPVTDDDNDRA	AT5G19390.4	Rho GTPase activation	1	0	0
	AT5G19390.3	Rho GTPase activation			
	AT5G19390.1	Rho GTPase activation			
	AT5G19390.2	Rho GTPase activation			
DNGGDVIDADFTDSN	AT5G49910.1	CPHSC70-2EAT SHOCK	1	1	0
DVSEGDESEGDVSEG	AT4G24770.1	RBP31, ATRBP31, CP31,	1	0	0
FFEGGFGSDDDPTSP	AT1G64510.1	Translation elongation factor	3	2	1
FVGGEETKSGGEEAE	AT5G53170.1	FTSH11	1	0	0
GDGTSDSDSDPDPPK	AT3G09050.1	unknown protein	2	0	0
GFNRLDDSAEFFTSD	AT2G36390.1	SBE2.1, BE3	1	0	0
GKKLSELSDDEDFDE	AT3G48500.1	PDE312, PTAC10	2	2	2
	AT3G48500.2	PDE312, PTAC10			
GLSDHDEYDLDGDNN	AT5G26742.1	emb1138 | DEAD box RNA	2	0	0
	AT5G26742.2	emb1138 | DEAD box RNA			
GSDKGILSDVELLKE	AT5G13630.1	GUN5, CCH, CHLH, CCH1,	1	0	0
	AT5G13630.2	GUN5, CCH, CHLH, CCH1,			
GSESEEETEEMIFGS	AT4G02510.1	TOC159, TOC86, PPI2,	3	2	2
IDGQIVTDSDEDVDT	AT4G02510.1	TOC159, TOC86, PPI2,	1	1	0
KAEKDEVSDDEATIE	AT1G58200.1	MSL3	1	2	0
	AT1G58200.2	MSL3			
KDDDEDDQSSDGHED	AT3G52230.1	unknown protein	1	0	0
KGFVADDSDIESPRD	AT5G19390.2	Rho GTPase activation	1	0	0
	AT5G19390.1	Rho GTPase activation			

	AT5G19390.4	Rho GTPase activation			
	AT5G19390.3	Rho GTPase activation			
KYEGKKLSELSDDED	AT3G48500.1	PDE312, PTAC10	2	3	2
	AT3G48500.2	PDE312, PTAC10			
LSEEAFKSLGLSDHD	AT5G26742.1	emb1138 | DEAD box RNA	2	0	0
	AT5G26742.2	emb1138 | DEAD box RNA			
LSKSGDGTSDSDSDP	AT3G09050.1	unknown protein	2	0	0
LSYLPDLSDVELAKE	AT5G38420.1	Ribulose bisphosphate	2	0	0
	AT5G38410.2	Ribulose bisphosphate			
	AT5G38410.1	Ribulose bisphosphate			
	AT5G38410.3	Ribulose bisphosphate			
NSSVEEETEEEVEED	AT5G08540.1	unknown protein	1	0	0
PDFKIRATDIDDEWG	AT4G04020.1	FIB	1	0	0
PFDSDDESDNKHTLN	AT5G61210.1	SNAP33, ATSNAP33,	3	2	2
QSESEEGSDDEEEEA	AT2G28800.4	ALB3	2	2	2
	AT2G28800.1	ALB3			
RGLAYDTSDDQQDIT	AT2G39730.1	RCA	1	0	0
	AT2G39730.2	RCA			
	AT2G39730.3	RCA			
RWRGLAYDTSDDQQD	AT2G39730.1	RCA	1	1	0
	AT2G39730.2	RCA			
	AT2G39730.3	RCA			
SKPNPFDSDDESDNK	AT5G61210.1	SNAP33, ATSNAP33,	2	2	1
TDEWGEKSGPELEES	AT2G35490.1	Plastid-lipid associated	1	0	0
VEESQSESEEGSDDE	AT2G28800.4	ALB3	2	2	1
	AT2G28800.1	ALB3			
VLAESESSAFEDQCG	AT5G14740.5	CA2, CA18, BETA CA2	1	0	0
	AT5G14740.3	CA2, CA18, BETA CA2			
	AT5G14740.4	CA2, CA18, BETA CA2			
	AT5G14740.1	CA2, CA18, BETA CA2			
	AT5G14740.2	CA2, CA18, BETA CA2			
VTRVDGSESEEETEE	AT4G02510.1	TOC159, TOC86, PPI2,	1	1	0
